# Engineering Iridium–Ruthenium Dual‐Atomic Active Sites on Redox‐Active Covalent Organic Frameworks for Boosted Overall Water Splitting

**DOI:** 10.1002/smll.202510427

**Published:** 2026-01-08

**Authors:** Lei Ran, Yifan Xu, Yue Zhang, Jinsong Zhou, Mingzi Sun, Yingguang Zhang, Bei Ran, Chengxu Zhang, Jue Hu, Bolong Huang, Michael K. H. Leung

**Affiliations:** ^1^ School of Energy and Environment City University of Hong Kong Hong Kong China; ^2^ Faculty of Metallurgical and Energy Engineering Kunming University of Science and Technology Kunming China; ^3^ Department of Chemistry City University of Hong Kong Hong Kong China; ^4^ Department of Applied Biology and Chemical Technology The Hong Kong Polytechnic University Hong Kong China

**Keywords:** active sites separation mechanism, covalent organic frameworks, dual‐atom catalysts, intramolecular compensating electronic modulations, overall water splitting

## Abstract

The achievement of conductive bifunctional covalent organic frameworks (COFs) for overall water splitting remains challenging due to the lack of multifunctional active sites. Herein, the atomically dispersed electroactive iridium‐ruthenium dual‐active sites are anchored on donor‐acceptor‐based redox‐active COF (Ace and TAPT, IrRu DAS/AT‐COF) as a pyrolysis‐free electrocatalyst for alkaline water electrolysis application. The as‐synthesized IrRu DAS/AT‐COF exhibits robust bifunctional activities and stability for hydrogen evolution reaction (HER) and oxygen evolution reaction (OER) in 1 m KOH, surpassing the benchmarks and most recent noble‐metal‐based catalysts. Operando spectroscopy and theoretical calculations unveil the multiple active sites separation mechanism on IrRu DAS/AT‐COF, where the giant multifunctional active‐site synergistic enhancement (MASE) effect was triggered by the intramolecular compensating electronic modulations between the Ir_SA_ and Ru_SA_ sites. This setting can balance the competitive effects of the following elementary steps and simultaneously accelerate them into the local environment of catalytic units, including (i) the improved conductivity and H_2_O adsorption, (ii) decreased H_2_O dissociation energy barrier, (iii) optimal adsorption of H/O intermediates. This work provides new insights into the design of multi‐site catalytic local environments in bifunctional COFs electrocatalysts for water electrolysis.

## Graphical abstract

Atomically dispersed IrRu dual‐atomic sites are anchored on donor‐acceptor‐based redox‐active COF for overall water electrolysis, and the active sites separation mechanism induced by giant multifunctional active‐site synergistic enhancement (MASE) effect was explored by the operando spectroscopy and DFT calculations.

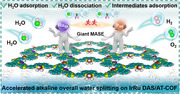

## 1 Introduction

Alkaline water electrolysis is essential to fulfill our increasing demand for green energy supply [1, 2]. Nonetheless, the slow reaction kinetics lead to high overpotentials for the hydrogen evolution reaction (HER) and oxygen evolution reaction (OER). Recently, considerable efforts have been made to develop low‐cost non‐noble metal‐based electrocatalysts [3, 4, 5]. Nevertheless, the low efficiency and poor stability greatly hinder their further applications. So far, noble metal‐based catalysts are still regarded as the most efficient electrocatalysts for water electrolysis application owing to their optimal adsorption toward various active intermediates species [6]. However, the high cost, resource scarcity, and inferior bifunctionality lead to expensive costs for water splitting, which largely limit the widespread application of alkaline water electrolysis. Therefore, enhancing the formation and exposure of the active sites is essential to reduce metal usage and improving metal mass activity while maintaining the high bifunctional catalytic performance [7].

Recently, covalent organic frameworks (COFs) received much attention due to their multiple inherent remarkable features, including large surface area, well‐defined porosity, structural periodicity, and incredible structure stability [8, 9]. The appropriate building units and functional organic groups endow COFs with a highly *π*–*π* conjugation structure and adjustable electronic characteristics. Thus, COFs have been widely employed for gas storage, separation, sensing, photocatalysis, and biomedical applications [10, 11]. The design and development of redox‐active COFs have received recent attention due to their potential for electrocatalysis applications [12]. However, the electroactive COFs prepared by integration of redox‐active organic units are still at a primitive stage. On the one hand, in alkaline media, the active intermediates are generated by breaking a strong covalent bond in H_2_O molecules. However, the pristine COFs still suffer from poor electrochemical performance due to insufficient active sites and sluggish catalytic kinetics. On the other hand, due to the different electrochemical reactions involve quite different intermediates [13], most of COFs‐based electrocatalysts reported so far can only catalyze a single chemical reaction, resulting in their application as a bifunctional electrocatalyst for overall water splitting is still very challenging [14, 15].

Single‐atom catalysts (SACs) represent a promising catalyst due to the well‐defined active centers and maximum atom utilization [16, 17]. Ruthenium (Ru), as one of the platinum‐group metals, has great potential in the development of efficient electrocatalysts candidate for water splitting application [18, 19]. Currently, various Ru‐based SACs have been developed for alkaline water electrocatalysis, such as Ru SAs@PN [20], RuCu‐CAT [21], R‐NiRu [22], RuCo‐CAT [23], etc. However, the Ru SACs with a single catalytic site are intrinsically limited by the simplicity of their active sites, it is typically difficult to break the scaling relationship of key intermediates (OH^*^/H^*^) on isolated Ru sites in alkaline water electrocatalysis, which leads to the competitive adsorption and thus severely limits its catalytic performance and bifunctional characteristics. Moreover, the stability of Ru‐based electrocatalyst is still an obstacle limiting their practical application, and its tendency to dissolve at high potentials will lead to reduced catalytic activity or even deactivation [24, 25].

To achieve efficient alkaline water electrocatalysis, a desired bifunctional electrocatalyst needs to be able to simultaneously support the following key events, including (i) the efficient H_2_O adsorption (Δ*E*_H2O_) [26], (ii) the low H_2_O dissociation energy barrier (ΔG_H2O_) [27], and (iii) the optimal adsorption of key intermediates (Δ*G*_int_) [28], which is still challenging. However, incorporating multiple active sites that could accelerate various elementary steps into a local environment is anticipated to achieve this multiple demands, particularly for complex reactions involving multiple intermediates like HER and OER. For instance, in the recently reported catalysts such as Ru‐W/WO_2_ [29], PtRu/CNT@MO_2‐x_(M = Sn, Ce) [30], and InCu/PCN [31], the binding energy of multiple intermediates can be regulated simultaneously by precisely designing the active moieties, thus largely improving catalytic activity. Moreover, recent studies have shown that the introduction of iridium (Ir) species can greatly enhance corrosion resistance and improve electrochemical stability. For example, combining Ir into layered double hydroxide (such as NiV‐LDH [32], NiCo‐LDH [33]), metal organic frameworks (such as Ni‐NDC [34]), and metal nanoclusters (such as Mo [35], Ni [36]) has been shown to significantly improve the electrochemical activity and stability simultaneously. Therefore, judiciously chosen organic building blocks can provide a donor‐acceptor‐based redox‐active platform that can anchor atomically dispersed electroactive noble‐metal ions (Ir, Ru) in the local environment of the COFs networks to build multifunctional active sites. However, redox‐active COF‐based dual single‐atom systems that enable overall water splitting remain scarcely demonstrated, and direct, operando insights into multifunctional active‐site synergistic enhancement (MASE) in COF‐based dual single‐atom catalysts under realistic conditions are rare. We tackle these gaps by co‐anchoring Ir and Ru single atoms in a COF, followed by an integrated interrogation that couples systematic experiments with high‐resolution electron microscopy, operando spectroscopic probes, and theory analysis.

In this work, atomically dispersed iridium‐ruthenium dual‐metals anchored on a donor‐acceptor‐based redox‐active COFs (IrRu DAS/AT‐COF) were designed and fabricated by a pyrolysis‐free post‐modification approach as a bifunctional electrocatalyst for efficient and stable overall water splitting application. The IrRu DAS/AT‐COF with ultralow metal loading delivers ultralow overpotentials of 39.3 mV at 10 mA cm^−2^ for HER and 251.2 mV at 10 mA cm^−2^ for OER in 1 m KOH, outperforming benchmarks and most other noble‐metal based catalysts. Then we explored in depth the catalytic site separation mechanism triggered by giant MASE on conductive IrRu DAS/AT‐COFs networks, which simultaneously optimizes the elementary steps for obtaining ideal alkaline water electrolysis performance. From the operando spectroscopy and density functional theory (DFT) calculation, the compensating electronic modulations between Ir_SA_ and Ru_SA_ sites in the multiple‐site catalyst can not only promote the H_2_O adsorption and activation, but also accelerate the H_2_O dissociation, and optimize the adsorption behavior of H and O‐containing intermediates, thus achieving superior HER and OER with the lowest energy barriers. Moreover, the intramolecular electron transport along the donor‐acceptor frameworks and structural stability largely enhance the electrical conductivity and catalytic stability in alkaline environments.

## 2 Results and Discussion

### 2.1 Fabrication and Characterization of IrRu DAS/AT‐COF

**FIGURE 1:**
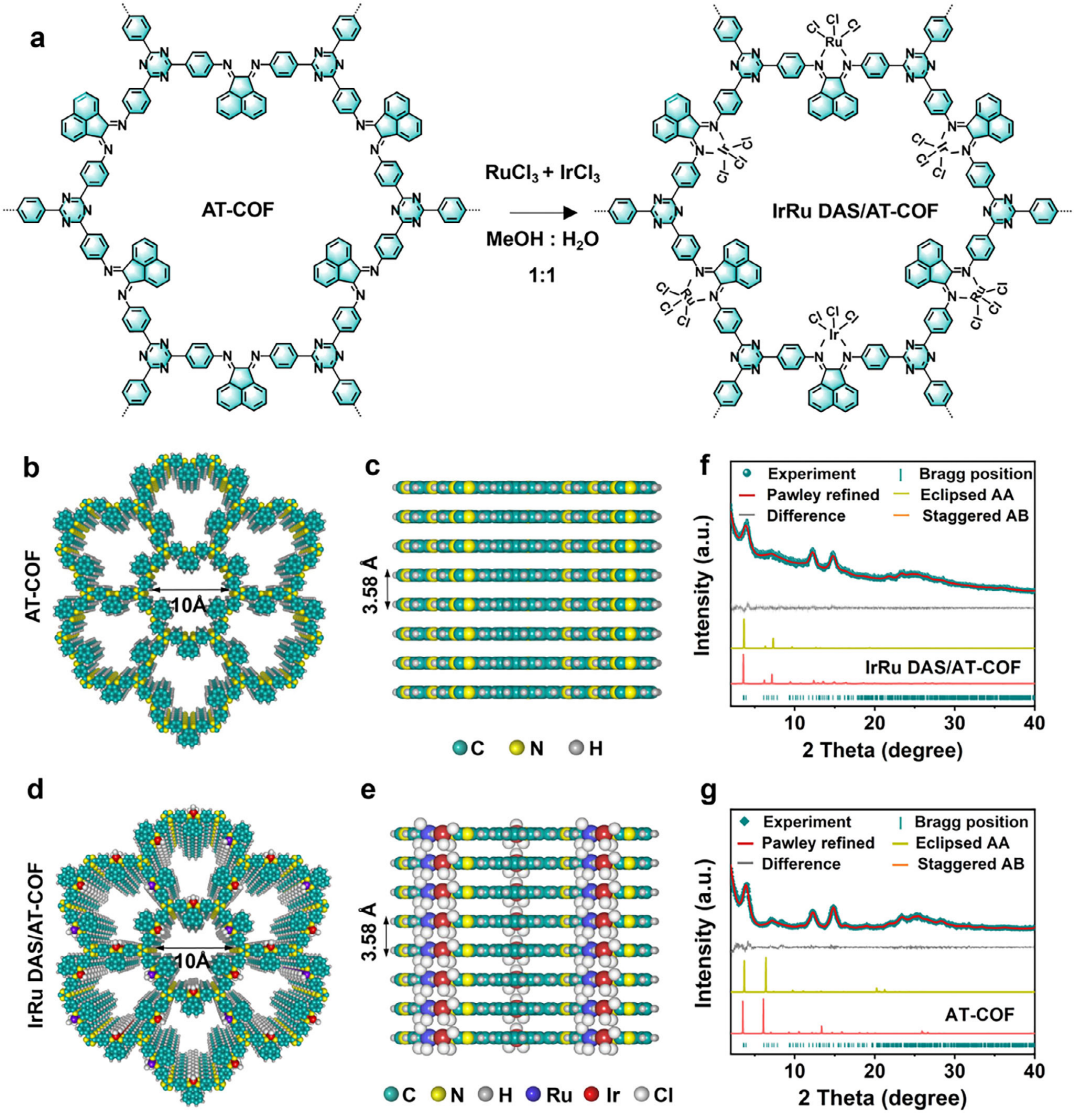
Synthetic diagram and characterization. (a) The synthesis of IrRu DAS/AT‐COF. (b,c) The top and side views of AT‐COF. (d,e) The top and side views of IrRu DAS/AT‐COF. (f,g) The PXRD plots of experiment AT‐COF (green) and IrRu DAS/AT‐COF (green), Pawley‐refined (red), difference (gray), the simulated PXRD plots of eclipsed AB stacked (orange), staggered AA stacked (yellow), and the Bragg positions (cyan).

To achieve the key objective of this advanced model, IrRu dual‐atom metal sites (IrRu DAS) were anchored on the covalent organic framework (AT‐COF) matrix with a nitrogen‐metal‐chlorine bridge (M─N─Cl, M = Ir and Ru), realizing the superior water splitting properties (Figure 1a). First, AT‐COF was acquired by the aggregation of ace‐naphthenequinone and 4,4’4″‐(1,3,5‐triazine‐2,4,6‐triyl)trianiline monomers. Then, the isolated IrRu DAS was introduced to the AT‐COF backbone by the post‐impregnation method, leading to the fabrication of IrRu DAS/AT‐COF (Figures S1 and S2). The planar configuration of Ace and TAPT building units yielded the high *π*–*π* connections of the neighboring layers, resulting in highly crystalline AT‐COF (Figure 1b,c) and IrRu DAS/AT‐COF (Figure 1d,e) [37]. From the powder X‐ray diffraction (PXRD) patterns (Figure 1f,g), the clear and obvious diffraction peaks observed confirm high crystallinity. The similar PXRD patterns between AT‐COF and IrRu DAS/AT‐COF demonstrate that the crystalline structure is maintained after incorporating IrRu DAS. Specifically, the diffraction peaks at 4.0°, 7.1°, 12.1°, and 14.7° are indexed to (100), (110), (130), and (400) planes, respectively, while the diffraction peak around 25.1° corresponding to (001) facet refers to the *π*–*π* stacked connections of the neighboring layers. All these diffraction peaks exhibit the hexagonal layered framework following the *P*‐*6* space group. Pawley refinement of experimental PXRD profiles disclose the unit cell parameters of IrRu DAS/AT‐COF (Table S1) and AT‐COF (Table S2), i.e., α = 90°, β = 90°, and γ = 120°, and a = 28.7705 Å, b = 28.7705 Å, and c = 3.5503 Å. The PXRD profiles simulated by the AA stacked mode align well with experimental results, whereas the AB stacked mode does not match. The different plots indicate that the R‐factors of the weighted‐profile (*R*_wp_ = 3.52%) and unweighted‐profile (*R*_p_ = 2.77%). The Ir SAS/AT‐COF and Ru SAS/AT‐COF were obtained by a similar method (Figures S3–S6).

**FIGURE 2:**
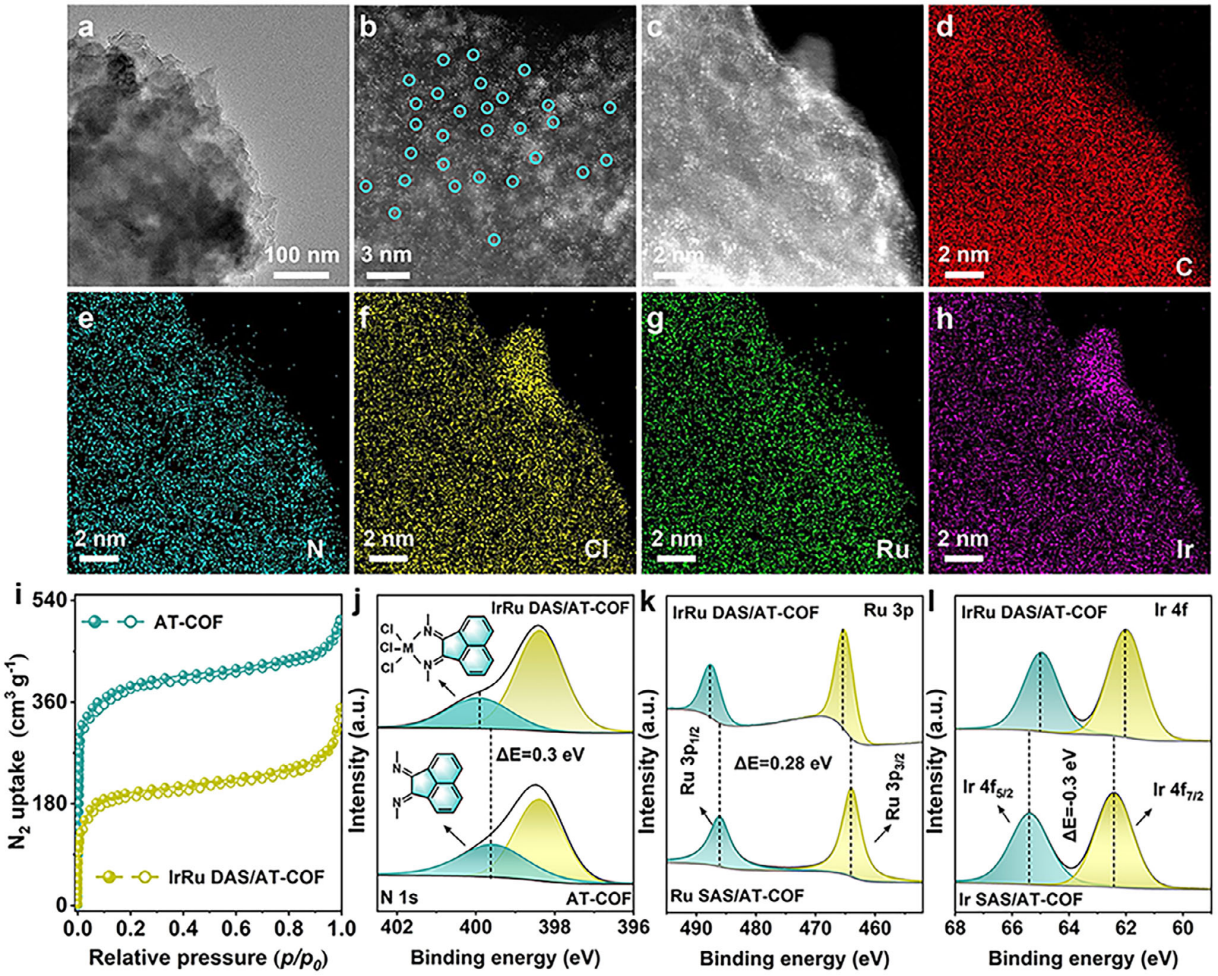
Morphology and microstructure characterization. (a) TEM image of IrRu DAS/AT‐COF. (b) Aberration corrected HAADF‐STEM image and (c–h) EDX mappings of IrRu DAS/AT‐COF, IrRu DAS are marked with green circles. (i) N_2_ adsorption‐desorption isotherms of IrRu DAS/AT‐COF and AT‐COF. (j) The N 1s XPS spectra, (k) Ir 4f spectra, and (l) Ru 3p XPS spectra of IrRu DAS/AT‐COF and AT‐COF.

Scanning electron microscopy (SEM) and transmission electron microscopy (TEM) were applied to analyze the surface morphology and nanostructure. The TEM image of IrRu DAS/AT‐COF reveals the abundant pores in COF networks (Figure 2a), consistent with SEM images of IrRu DAS/AT‐COF and AT‐COF (Figures S7 and S8). From the high‐resolution TEM (HRTEM) images, the observed lattice fringes of 0.36 and 0.60 nm are resulted from (001) and (400) facets for the hexagonal structure (Figure S9). These two diffraction rings observed in selected area electron diffraction (SAED) images are consistent with (130) and (100) facets for the hexagonal structure (Figure S10). Moreover, the aberration‐corrected high‐angle annular dark‐field scanning transmission electron microscopy (AC‐HAADF‐STEM) image exhibits obvious bright dots in pairs (Figure 2b), representing the isolated IrRu DAS in IrRu DAS/AT‐COF. The energy‐dispersive X‐ray (EDX) mapping images reveal the uniformly distributed N, C, Ir, Ru, and Cl elements in IrRu DAS/AT‐COF (Figure 2c–h) and C and N elements in AT‐COF (Figure S11), respectively. Moreover, Ir SAS/AT‐COF and Ru SAS/AT‐COF also show a similar microstructure (Figures S12–S17). Through inductively coupled plasma mass spectrometry (ICP‐MS), the loading content of Ru and Ir SAS species in the IrRu DAS/AT‐COF were obtained as 0.56 and 0.52 wt.%, respectively (Table S3).

The nitrogen (N_2_) absorption‐desorption analysis was performed to determine the porous structure of these samples (Table S4). Figure 2i exhibits similar type I curves, and the sharply increased gas uptake under low relative pressure (*P*/*P_0_* <0.1) proves the existence of abundant micropores. The Brunauer–Emmett–Teller (BET) surface areas and pore volumes are obtained for IrRu DAS/AT‐COF (534.23 m^2^ g^−1^ and 0.26 cm^3^ g^−1^) and AT‐COF (946.32 m^2^ g^−1^ and 0.32 cm^3^ g^−1^). Since the Ir or Ru species partially occupy the internal cavities and pores of COF, IrRu DAS/AT‐COF shows a reduced surface area, which has also been reported in other single‐atom catalysts [38, 39]. Through the pore size distribution curves, the pore sizes of IrRu DAS/AT‐COF and AT‐COF are determined as 0.99 nm (Figure S18), matching well with the simulated unit cells (1.0 nm). Despite the introduction of IrRu DAS into the interior cavities of AT‐COF backbone, the permanent open structure is well preserved, providing a high surface area and numerous active centers to promote the electrochemical reaction process. In the Fourier transform infrared spectroscopy (FTIR) spectra, IrRu DAS/AT‐COF, Ru SAS/AT‐COF, Ir SAS/AT‐COF, and AT‐COF have the same peaks (Figure S19), and the observed vibration band around 1400 and 1620 cm^−1^ is derived from ν (─C═N─) in pyridine rings and imine bonds, respectively [40]. The observed peak at 1600 cm^−1^ in Raman spectra results from C═N or C═C bondings in AT‐COF‐based electrocatalysts (Figure S20) [41].

The nuclear magnetic resonance spectroscopy (NMR) spectra of ^13^C and ^1^H exhibit clear signals at 156 ppm, corresponding to C═N chemical bonding (Figure S21), indicating the fabrication of the triazine‐based COFs through the condensation process [42]. Besides, the chemical stability and thermal stability of COFs were explored to realize their practical applications. The chemical stability was evaluated by soaking the catalyst in various solvents (DMF, methanol, acetonitrile, toluene, NaOH, and HCl) for several days. There are no obvious changes in PXRD profiles (Figure S22) after exposure in different environments, indicating the remarkable structural robustness of IrRu DAS/AT‐COF under extreme conditions. The thermo gravimetric analysis (TGA) further indicates that these COFs‐based catalysts possess excellent thermal stability under an air atmosphere (Figure S23). From contact angles (CA) tests (Figure S24), IrRu DAS/AT‐COF has a smaller CA value of 41.2° than AT‐COF (66.8°), indicating the enhanced super‐hydrophilicity can improve the interaction between catalyst and electrolyte [43]. In temperature‐programmed desorption‐H_2_ (H_2_‐TPD) plots, IrRu DAS/AT‐COF shows the lowest H_2_ desorption peak, indicating that H_2_ is easier to escape from the catalyst than other catalysts (Figure S25).

The full range X‐ray photoelectron spectroscopy (XPS) spectra indicate the existence of N, C, Cl, Ir, and Ru species (Figure S26). The N 1s XPS spectra (Figure 2j; Figures S27 and S28) show that the N 1s signal is ascribed to two nitrogen configurations at 399.8 and 398.7 eV [44], corresponding to imine‐N and pyridinic‐N, respectively. Compared with AT‐COF, the imine‐N peak of IrRu DAS/AT‐COF shows a redshift to higher binding energy owing to the coordination of N with metal atoms, and a similar phenomenon was reported in the other N‐metal coordination catalysts [38]. The two binding energies of Ru 3p_1/2_ and Ru 3p_3/2_ at 486.2 and 464.7 eV, respectively, in the Ru 3p XPS spectra prove the presence of Ru^3+^ ions (Figure 2k) [45], while the Ir 4f XPS spectra (Figure 2l) can be deconvoluted to Ir^3+^ state with two binding energies of Ir 4f_5/2_ and Ir 4f_7/2_ at 65.3 and 62.4 eV, respectively [46]. Besides, the Ru 3p and Ir 4f spectra show the positive (0.28 eV) and negative (−0.3 eV) shift to higher and lower binding energy, respectively, indicating the charge transfer from Ru_SA_ to Ir_SA_ sites to realize the optimized electronic structure of IrRu DAS/AT‐COF. The shifted binding energy of Ir and Ru particularly indicates the corresponding shifted d‐band center after forming the IrRu DAS, which regulates the bonding and antibonding states to optimize the binding strength between Ir/Ru sites and active intermediates [43], resulting in the improved water splitting performance in the IrRu DAS/AT‐COF electrocatalyst.

**FIGURE 3:**
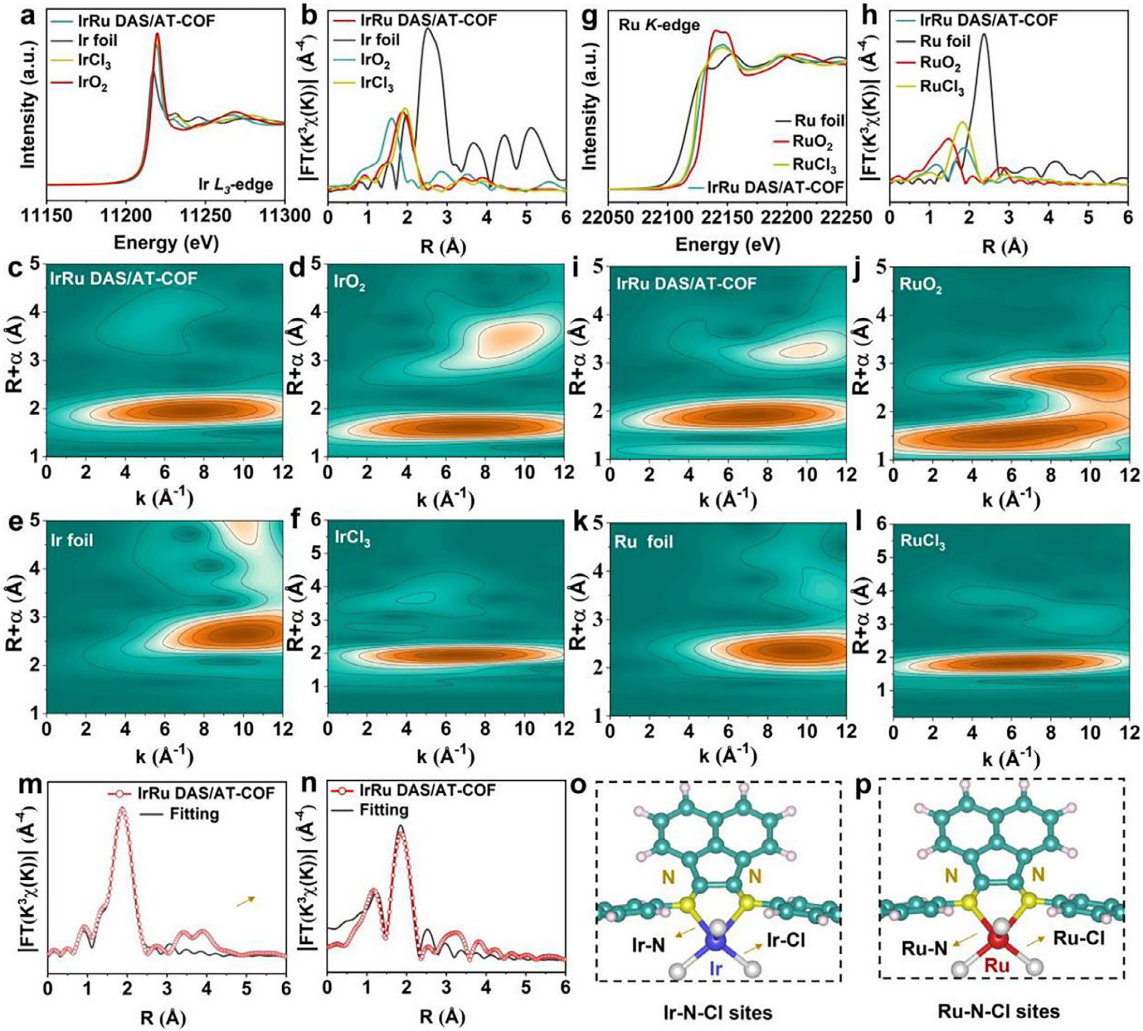
Dual atomic structure analysis of IrRu DAS/AT‐COF by XAFS. (a) Normalized XANES, (b) Fourier‐transformed EXAFS, and (c–f) WT‐EXAFS of IrRu DAS/AT‐COF and references at Ir L_3_‐edge. (g) Normalized XANES, (h) Fourier‐transformed EXAFS, and (i–l) WT‐EXAFS of IrRu DAS/AT‐COF and references at Ru K‐edge. (m) Ir L_3_‐edge and (n) Ru K‐edge EXAFS fitting plots of IrRu DAS/AT‐COF at R space. (o,p) The schematic model and optimized bond length of IrRu DAS/AT‐COF, C (cyan), N (yellow), H (pink), Ir (blue), Ru (red), and Cl (white).

The X‐ray absorption fine structure spectroscopy (XAFS) was employed to probe the local atomic environments of IrRu DAS. Figure 3a shows the Ir L_3_‐edge X‐ray absorption near‐edge structure (XANES) curves. The near‐edge absorption peak of IrRu DAS/AT‐COF is positioned between Ir foil and IrO_2_ and almost coincides with IrCl_3_, revealing that the Ir^3+^ species are formed. In the Fourier‐transformed (FT) extended X‐ray absorption fine structure (EXAFS) plots (Figure 3b), compared with IrO_2_, IrCl_3,_ and Ir foil, IrRu DAS/AT‐COF displays two main peaks at 1.87 and 1.5 Å, which are attributed to Ir‐N/Cl coordinations [47]. Moreover, IrRu DAS/AT‐COF reveals no peak at 2.5 Å corresponding to Ir–Ir, which indicates the atomically dispersed Ir atoms in IrRu DAS/AT‐COF [48]. Figure 3c–f presents wavelet transformation (WT) curves of Ir L_3_‐edge‐weighted EXAFS. The WT maximum of IrRu DAS/AT‐COF at 7.5 Å^−1^ is apparently different from IrO_2_ and Ir foil, suggesting that Ir atoms are atomically dispersed on IrRu DAS/AT‐COF. Similarly, the near‐edge absorption peak in Ru K‐edge XANES spectra, which lies between Ru foil and RuO_2_ and almost coincides with RuCl_3_, indicates the existence of Ru^3+^ (Figure 3g); and the obviously observed major peak and shoulder peak in FT‐EXAFS curve are attributed to atomic coordination of Ru with N/Cl atoms (Figure 3h) [49]. The characteristics of the maxima in the WT of EXAFS (Figure 3i–l) differentiate from Ru foil and RuO_2_ but are similar to RuCl_3_, indicating the atomically dispersed Ru atoms on IrRu DAS/AT‐COF [50]. EXAFS fitting curves were conducted to confirm the coordinated modes of metal species (Figure 3m,n; Figure S29 and Table S5). The Ir atoms are fixed to the COF backbone at the atomic level, forming bonds with two N atoms at an atomic distance of 2.12 Å (Ir─N) and three Cl atoms at an atomic distance of 2.38 Å (Ir─Cl). Meanwhile, the Ru spectra could be nicely fitted by two scattering paths of Ru─N (2.09 Å) and Ru─Cl (2.35 Å) with coordinated numbers of 2 and 3, respectively. The density functional theory (DFT) was used to simulate the coordination environment of IrRu DAS/AT‐COF. The presented local atomic configuration shows that the bond lengths of Ir/Ru─N (2.10 Å) and Ir/Ru─Cl (2.36 Å) are obtained by optimizing Ir/Ru─N‐coordinated COF skeleton (Figure 3o,p), where the N/Cl atoms can modulate and stabilize the coordination environment of IrRu DAS, in accordance with EXAFS results [49].

### 2.2 Giant MASE for Overall Water Splitting Using IrRu DAS/AT‐COF

**FIGURE 4:**
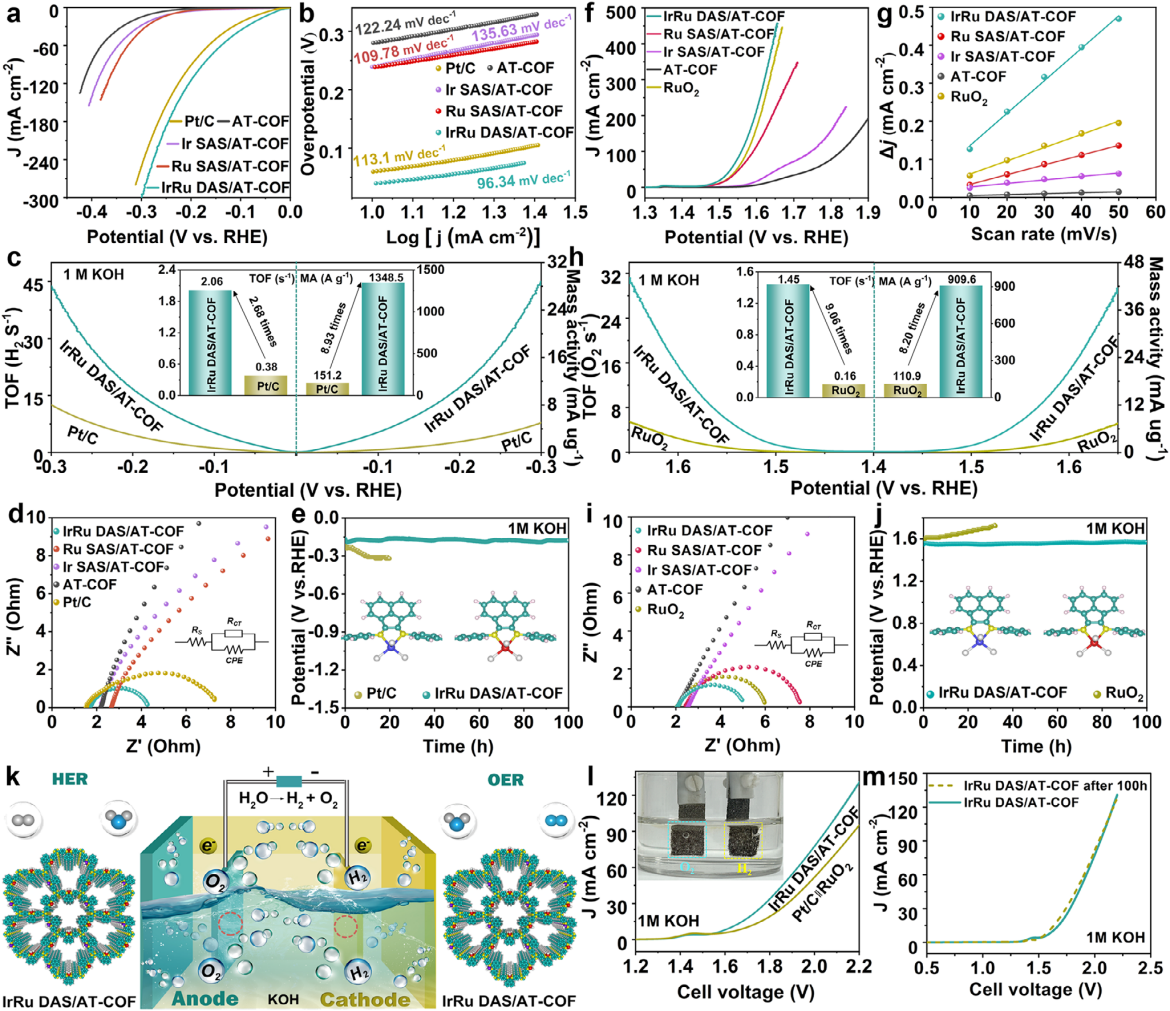
Giant MASE of IrRu DAS/AT‐COF for water splitting. (a) LSV curves, (b) Tafel plots, (c) TOF and mass activity curves, and (d) EIS plots for various electrocatalysts for HER. (e) The long‐term plots of IrRu DAS/AT‐COF and Pt/C at 100 mA cm^−2^ for the 100 h HER test. (f) LSV curves, (g) C_dl_ plots, (h) TOF and mass activity curves, (i) EIS plots of various electrocatalysts for OER. (j) The chronopotentiometry curves of IrRu DAS/AT‐COF at 100 mA cm^−2^ for a 100 h long‐term OER test. (k) The scheme of IrRu DAS/AT‐COF||IrRu DAS/AT‐COF for overall water splitting. (l) LSV curves of IrRu DAS/AT‐COF‖IrRu DAS/AT‐COF and Pt/C||RuO_2_ for overall water splitting in 1 m KOH. (m) LSV curves of IrRu DAS/AT‐COF‖IrRu DAS/AT‐COF before and after 100 h.

The electrocatalytic HER activity of these catalysts was first tested under 1.0 m KOH. IrRu DAS/AT‐COF achieves a considerably low overpotential (η_10_) of 39.3 mV at 10 mA cm^−2^ compared with Ru SAS/AT‐COF (237.9 mV), Ir SAS/AT‐COF (240.2 mV), AT‐COF (280.0 mV), and Pt/C (59.6 mV) (Figure 4a; Figure S30a). Such a performance surpasses most recent noble‐metal‐based HER catalysts (Figure S30b and Table S6). Besides, IrRu DAS/AT‐COF displays a lower Tafel slope of 96.3 mV dec^−1^ than Ru SAS/AT‐COF (109.8 mV dec^−1^), Ir SAS/AT‐COF (135.6 mV dec^−1^), AT‐COF (122.2 mV dec^−1^), and Pt/C (125.9 mV dec^−1^), revealing the giant MASE on IrRu DAS for higher HER kinetics (Figure 4b). With reference to the Tafel slope, the HER process on IrRu DAS/ AT‐COF is dominated by the Volmer‐Heyrovsky pathway (H_2_O + M─H^*^ + e^−^ ⇌ M + H_2_ + OH^−^), where the water dissociation represents the rate‐determining step and the DAS species can expedite the formation of H^*^ from H_2_O [51]. Thus, the giant MASE of DAS can largely enhance the H_2_O adsorption and reduce the water dissociation energy barrier, thus accelerate the H^*^ production [52], while the cleavage of the HO─H bond is kinetically hindered for SAS/AT‐COF and AT‐COF. Moreover, the much higher exchange current density (j_0_) of IrRu DAS/AT‐COF further confirms its remarkable HER activity (Figure S31). Furthermore, we conducted comparative electrochemical test of Ir‐SAS/AT‐COF+Ru‐SAS/AT‐COF mixture and IrRu‐DAS/AT‐COF. As shown in Figure S32, the physical mixtures consistently underperformed the IrRu‐DAS/AT‐COF catalyst, therefore intuitively reflecting the giant MASE in the IrRu‐DAS/AT‐COF catalyst.

To gain insight into the excellent performance, the double‐layer capacitance (*C*_dl_) and electrochemical active surface area (ECSA) were investigated [24]. As IrRu DAS/AT‐COF shows much higher *C*_dl_ of 17.23 mF cm^−2^ than Ru SAS/AT‐COF (2.58 mF cm^−2^), Ir SAS/AT‐COF (1.84 mF cm^−2^), AT‐COF (0.29 mF cm^−2^), and Pt/C (7.98 mF cm^−2^), the DACs framework acquires a larger active surface area (Figure S33). The larger geometric current density normalized by ECSA (j_ECSA_) of IrRu DAS/AT‐COF confirms its better intrinsic HER activity (Figure S34). Then turnover frequency (TOF) and mass activity were calculated based on metal loading (Figure 4c) [43, 53]. IrRu DAS/AT‐COF shows a larger TOF of 2.06 H_2_ s^−1^ at η = 50 mV than Pt/C (0.38 H_2_ s^−1^), confirming its prominent catalytic performance. IrRu DAS/AT‐COF also shows an extraordinary mass activity, especially, a much higher mass activity of 1348.5 A g^−1^ at η = 50 mV, about 8.93 times larger than Pt/C (151.2 A g^−1^). This superior performance strongly correlates to the unique DAS structure, which provides modulated electronic structure and electrical conductivity, resulting in enhanced electron migration between the electrocatalyst and intermediates. Moreover, from the electrochemical impedance spectroscopy (EIS) spectra, IrRu DAS/AT‐COF exhibits lower electron transfer resistance (R_ct_) (2.61 Ω) than Ru SAS/AT‐COF (28.51 Ω), Ir SAS/AT‐COF (28.05 Ω), AT‐COF (59.69 Ω), and Pt/C (5.64 Ω), indicating the IrRu DAS can largely boost the electron transport kinetics (Figure 4d) [31, 54]. From the chronopotentiometry test at 100 mA cm^−2^, the HER performance of Pt/C declines significantly while IrRu DAS/AT‐COF exhibits negligible potential decay after 100 h (Figure 4e), indicating its excellent electrochemical stability. Moreover, SEM and TEM images prove that IrRu DAS/AT‐COF can preserve the morphology and nanostructure after stability tests (Figures S36 and S37). AC‐STEM image further proves the presence of IrRu DAS in IrRu DAS/AT‐COF through100 h test (Figure S38). Besides, there are no noticeable changes in both PXRD pattern and Ir 4f and Ru 3p XPS spectra after 100 h (Figures S39 and S40), which originates from the stabilization of SAS metal centers through the coordinated interaction and confinement effect between SAS metal atoms and binding groups [55].

The oxygen evolution reaction (OER) performance was then evaluated for AT‐COF, Ru SAS/AT‐COF, Ir SAS/AT‐COF, and RuO_2_ in 1 m KOH. As shown in Figure 4f and Figure S41a, the IrRu DAS/AT‐COF catalyst exhibits much lower η_10_ (251.2 mV) than Ru SAS/AT‐COF (268.3 mV), Ir SAS/AT‐COF (352.0 mV), AT‐COF (387.8 mV), and commercial RuO_2_ (266.5 mV). Such an OER electrocatalytic activity of IrRu DAS/AT‐COF is superior to most recently reported noble‐metal‐based OER catalysts with high noble‐metal loading (Figure S41b and Table S7). The Tafel slope value was calculated as low as 67.8 mV dec^−1^ for IrRu DAS/AT‐COF, which is lower than those of Ru SAS/AT‐COF (70.3 mV dec^−1^), Ir SAS/AT‐COF (85.7 mV dec^−1^), AT‐COF (133.5 mV mV dec^−1^), and RuO_2_ (68.3 mV dec^−1^), indicating the faster electrocatalytic OER kinetics (Figure S42). In addition, IrRu DAS/AT‐COF reveals the larger C_dl_ value of 8.54 mF cm^−2^ than other catalysts, indicating that DACs have high intrinsic catalytic activity due to more active sites exposed (Figure 4g; Figure S43). Furthermore, IrRu DAS/AT‐COF possesses higher mass activity and TOF values than RuO_2_, with an ultrahigh mass activity of 909.6 A g^−1^ and a TOF value of 1.45 s^−1^ at 1.5 V vs. RHE, suggesting its improved intrinsic activity (Figure 4h). Besides, the smaller R_ct_ of IrRu DAS/AT‐COF (3.0 Ω) also verified its fastest kinetics during the OER process (Figure 4i). Similarly, we compared the OER activities of Ir‐SAS/AT‐COF+Ru‐SAS/AT‐COF mixture and IrRu‐DAS/AT‐COF, the physical mixture shows lower OER activity than IrRu‐DAS/AT‐COF, thus intuitively reflecting the giant MASE in the IrRu‐DAS/AT‐COF (Figure S35). Moreover, the long‐term test at 100 mA cm^−2^ confirms the superior OER stability of IrRu DAS/AT‐COF (Figure 4j). From the XRD and HRTEM results, the well‐preserved morphology and homogeneous distribution of N, C, Ir, Ru, and Cl elements further proves the long‐term electrochemical stability (Figure S44). Inspired by the highly effective and stable HER and OER reaction, the two‐electrode overall water splitting cell was constructed to evaluate the bifunctional catalytic activity for overall water splitting in 1.0 m KOH using the IrRu DAS/AT‐COF catalyst as both cathode and anode (Figure 4k). Surprisingly, the IrRu DAS/AT‐COF||IrRu DAS/AT‐COF cell only needs a low cell voltage of 1.61 V to achieve the current density of 10 mA cm^−2^ to drive the overall water splitting, which is superior to Pt/C||RuO_2_ cell (1.68 V), and enormous bubbles can be observed both on the anode and cathode (Figure 4l). Furthermore, from the chronopotentiometry test and LSV curves after long‐term test, different from the low stability of Pt/C||RuO_2_ (Figure S45), IrRu DAS/AT‐COF||IrRu DAS/AT‐COF cell shows the negligible changes in current densities and voltage attenuation after 100 h (Figure 4m; Figure S46), confirming its excellent electrochemical stability. The superior performance outperforms the most recent reported electrocatalysts, endowing the IrRu DAS/AT‐COF catalyst the great commercial potential toward alkaline overall water splitting application (Figure S47 and Table S8).

### 2.3 Mechanistic Insights into Giant MASE of IrRu DAS/AT‐COF

**FIGURE 5:**
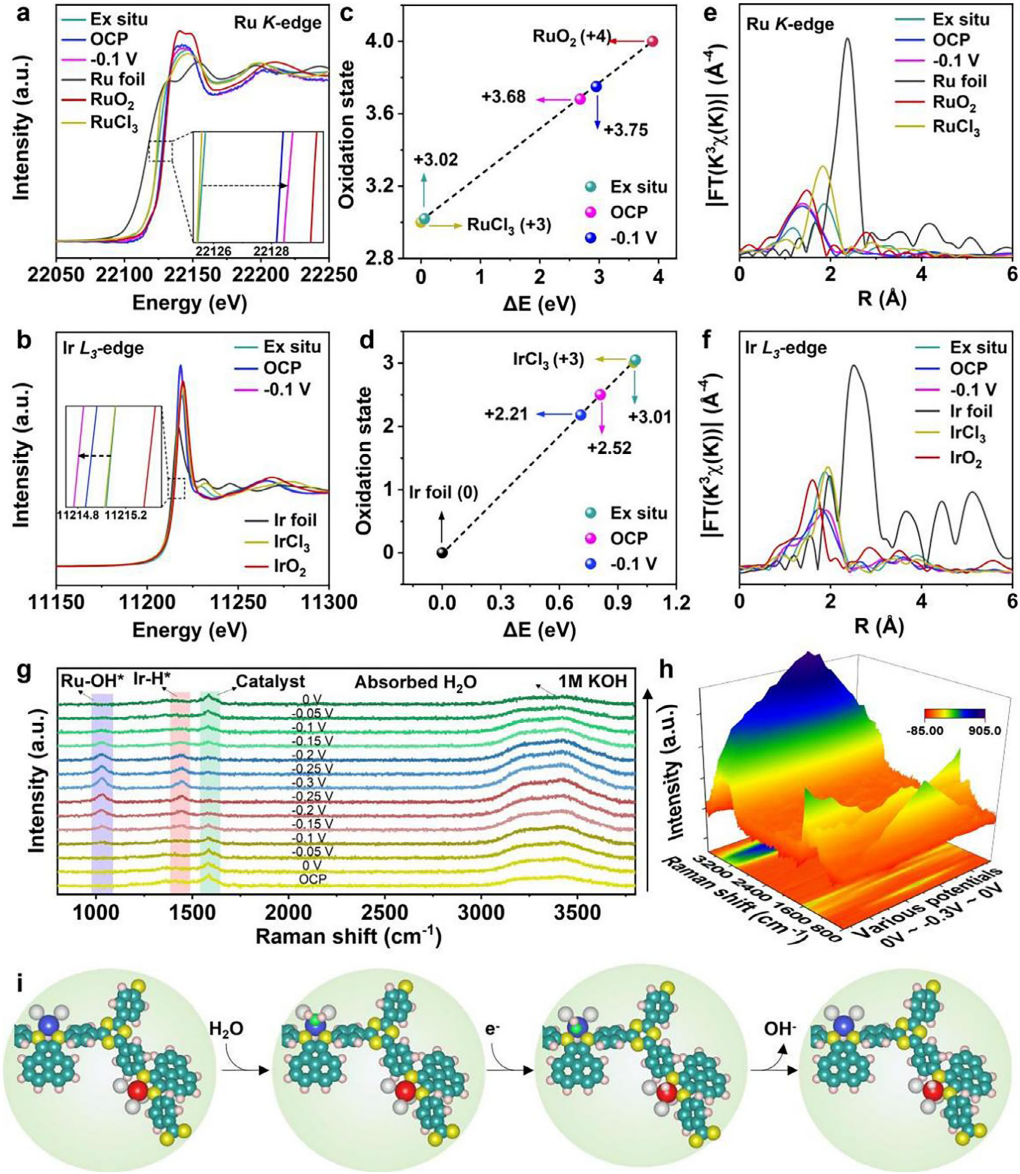
The operando analysis of giant MASE on IrRu DAS/AT‐COF. The operando XANES spectra of IrRu DAS/AT‐COF at (a) Ru K‐edge and (b) Ir L_3_‐edge, respectively. The fitted average oxidation state changes of (c) Ru and (d) Ir from Ru K‐edge and Ir L_3_‐edge XANES spectra at various potentials. Corresponding FT‐EXAFS spectra of (e) Ru K‐edge and (f) Ir L_3_‐edge. (g,h) The operando Raman spectra of IrRu DAS/AT‐COF under various applied potentials. (i) Schematic illustration of the hydrogen evolution mechanism determined by operando XAS and Raman analysis of IrRu DAS/AT‐COF in alkaline media.

To elucidate the origins of the high catalytic activities of IrRu DAS/AT‐COF, the operando XAS studies were performed under alkaline conditions to monitor the dynamic changes in the electronic structure and local atomic environment of IrRu DAS moieties. During the measurements, the working electrode potential increased in steps from the open circuit potential (OCP) to −0.1 V vs. RHE. Figure 5a shows the operando Ru K‐edge XANES spectra of IrRu DAS/AT‐COF recorded at different operating potentials. As the potential increases from OCP to −0.1 V (vs. RHE), the absorption edge of Ru K‐edge XANES spectra shifts toward higher energy, implying an increase in the Ru valence state during the water electrolysis. Meanwhile, the operando XANES spectra at the Ir L_3_‐edge of the IrRu DAS/AT‐COF are also recorded in Figure 5b, in which the distinct shift toward lower energy of the Ir absorption edge indicates the decreased Ir valence state. These results demonstrate that bias‐induced charge redistribution occurs across various metal sites of the COF framework. The Ru_SA_ sites favorably transfer electrons to Ir_SA_ sites through the intramolecular covalent network, which can modulate the state of the adsorbed reactive intermediates, thereby improving the catalytic activity of the IrRu DAS/AT‐COF. On the one hand, since water as a typical polar molecule, consists of two H atoms carrying a positive charge and an oxygen atom carrying a negative charge, its oxygen atom could be easily captured by a positively charged Ru atom in IrRu DAS/AT‐COF, thus completing the adsorption and activation of the water molecule. On the other hand, the increased electron density centered at Ir_SA_ moieties would result in more favorable adsorption of ^*^H_ads_ intermediates during water electrolysis, thus synergistically boosting the electrocatalytic activity of IrRu DAS/AT‐COF. Then, the average oxidation states of metal sites under the working conditions are confirmed by fitting the XANES spectra (Figure 5c,d). The Ru average valence state increases from +3.02 at ex situ state to +3.75 at −0.1 V (vs. RHE), while the Ir oxidation state decreases from +3.01 at ex situ state to +2.21 at −0.1 V (vs. RHE). Moreover, the local atomic structure evolutions of Ru_SA_ and Ir_SA_ sites were explored by EXAFS spectra under operando conditions. Both the EXAFS k^3^χ(k) functions of Ir L_3_‐edge and Ru K‐edge under various potential exhibit different oscillation patterns compared with ex situ spectra (Figure S48). The finding reveals not only the obvious change in electronic structure of metal atoms as explored by the operando XANES but also the rearrangement in local atomic structure at Ru_SA_ and Ir_SA_ sites. The results are further verified by FT‐EXAFS spectra of the Ru K‐edge and Ir L_3_‐edge of the IrRu DAS/AT‐COF. From the corresponding FT‐EXAFS spectra, no peaks of Ir─Ir and Ru─Ru coordinations are detected, indicating that the atomically dispersed Ir and Ru atoms are well preserved (Figure 5e,f). The changed R value and intensity of Ir/Ru─O/N coordination peak also demonstrate the varying coordination environment of Ir/Ru sites, originated from the absorption and activation of H_2_O and formation of hydrogen/oxygen‐containing intermediates [56]. Specifically, for Ru, the major peak in the FT‐EXAFS spectra at 1.5 Å can be assigned to the Ru─N or Ru─O coordination. Under the reduction potentials applied, the Ru─O/N peaks in the working catalyst display an obvious high‐R shift and the increased intensity with an increase of the applied potentials, which suggests the adsorption of more highly electronegative ^*^OH_ads_ on the high‐oxophilic Ru atoms, resulting in the redshift of absorption edge as observed in the XANES spectra. For Ir, as shown in the FT‐EXAFS spectra, the decreased intensity and low‐R shift observed from the Ir─N/O peaks under the reduction potentials corresponded to the reduced Ir valence state shown in operando XANES results, which might be attributed to the interaction of the Ir atom with the spilled ^*^H_ads_ species, resulting in a decrease of the coordination number and change of the atomic environment around Ir atoms. Moreover, under operando conditions, different characteristics of the maxima in the WT of EXAFS with Ir/Ru foil further indicates the atomically dispersed Ir and Ru metal atoms on IrRu DAS/AT‐COF when the water splitting takes place (Figures S49 and S50). The EXAFS curve‐fitting analysis was further performed under operando conditions to confirm the evolution of the local atomic structure of Ru_SA_ and Ir_SA_ in IrRu DAS/AT‐COF. As shown in Figures S51 and S52 and Table S8, the Ir/Ru‐N coordination number decreases from 2.0 to 1.1, indicates Ir/Ru atoms being released from the COF support to chemically adsorb H_2_O and H/O‐containing intermediates, thus forming the new Ru─O coordination. Therefore, the active IrRu DAS sites become freer under the working conditions, and enhance the activity of the exposed metal sites, enhancing the catalytic performance [57]. The slightly changed Ir/Ru─N bond lengths under the working conditions also imply that the DAS has a flexible state under reaction conditions, which are possibly induced by regulating the Ir/Ru─N bond length to chemical adsorption of H_2_O and different intermediates.

The operando Raman spectroscopy was performed to confirm disclose the active centers and intermediates in IrRu DAS/AT‐COF(Figure 5g,h). The main peak at 1600 cm^−1^ at 0 V is the same as the dry catalyst (Figure S20). After exerting and increasing the potential (0 to −0.3 V), two new peaks emerge and intensify simultaneously, which agree well with Ir─^*^H_ads_ stretching vibration and Ru─^*^OH_ads_ stretching vibration, respectively [58, 59]. This result implies that the Ru atoms are the actual active sites for water adsorption and dissociation, while the Ir atoms are the actual active sites for H^*^ adsorption and desorption during water splitting to hydrogen evolution, which is consistent with the above operando XAS results. Interestingly, this Ir─^*^H_ads_ and Ru─^*^OH_ads_ peak disappeared as the potential varies from −0.3 to 0 V, revealing that the metal sites possess distinct reversibility and stability [8]. However, the characteristic peak of IrRu DAS/AT‐COF exhibits a contrary trend, which first disappears as the potential decreases from 0 to −0.3 V, and then reappears as the potential returns to 0 V, proving that active intermediates are extremely easy to adsorb and desorb from highly active metal sites, thereby benefiting the water splitting process. Therefore, the detailed process of alkaline water splitting to hydrogen production through the Volmer‐Heyrovsky pathway is presented (Figure 5i). First, the IrRu DAS/AT‐COF catalyst can expose abundant Ru_SA_ as the initial active site for H_2_O activation and dissociation. Under the working conditions, the metal‐support interaction becomes weak, and the Ru_SA_ sites with modulated electronic structure become freer to form ^*^OH_ads_ and ^*^H_ads_ through the rapid Volmer step, which is consistent with the Tafel analysis. Simultaneously, the generated ^*^H_ads_ can be adsorbed at the nearby empty Ir_SA_ site via the hydrogen spillover effect, since the metallized Ir species with rich electron density are highly favorable for ^*^H_ads_ adsorption and desorption, thus acting as active centers for hydrogen evolution. Eventually, ^*^H_ads_ reacts with another proton formed by the dissociation of the adjacent H_2_O molecule via the Heyrovsky step, and eventually generates H_2_ at the Ir_SA_ site. The above results indicate that the co‐existence of Ir_SA_ and Ru_SA_ triggers the catalytic site separation mechanism, in which the Ru_SA_ species promotes water adsorption‐dissociation, while the Ir_SA_ species act as centers for the hydrogen evolution reaction, thus simultaneously optimizing the adsorption behaviors of various intermediate species in the water splitting reaction. Therefore, the above operando studies provide comprehensive and direct insights into the reaction kinetics taking place on the IrRu DAS/AT‐COF matrix during the alkaline water splitting process, featuring a catalytic site separation mechanism between the binary constituents as illustrated in Figure 5i.

**FIGURE 6:**
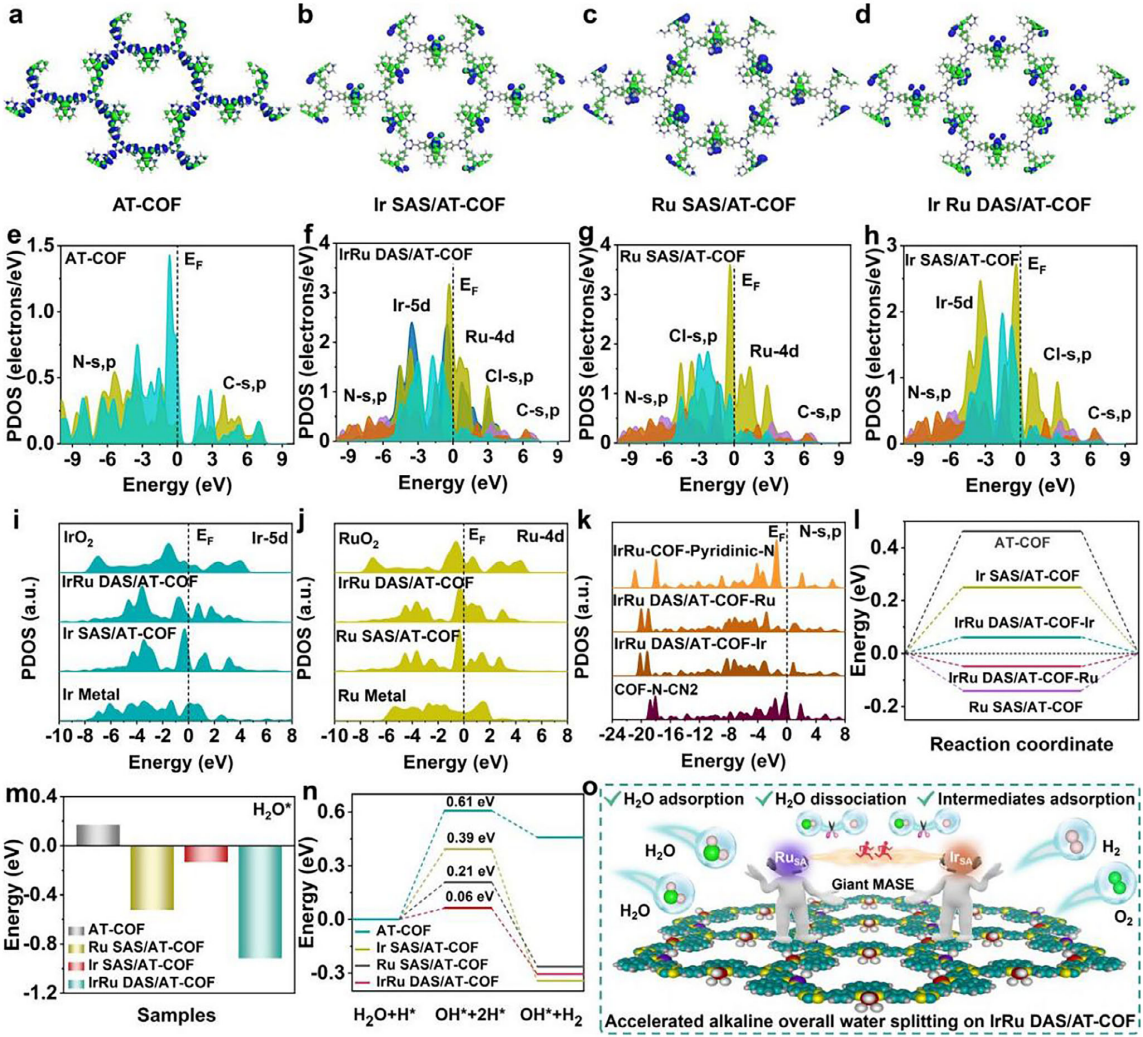
DFT calculation analysis of giant MASE on IrRu DAS/AT‐COF. The electronic distributions of the bonding and anti‐bonding orbitals near E_f_ in (a) AT‐COF, (b) Ir‐SAS/AT‐COF, (c) Ru SAS/AT‐COF, and (d) IrRu DAS/AT‐COF, blue isosurface = bonding orbitals and green isosurface = anti‐bonding orbitals. (e) The PDOS of AT‐COF, (f) IrRu DAS/AT‐COF, (g) Ru SAS/AT‐COF, and (h) Ir SAS/AT‐COF. (i) The PDOS comparison of Ir‐5d, (j) Ru‐4d, and (k) N‐s,p orbitals. (l) The adsorption energies of protons, (m) the adsorption energies of H_2_O, and (n) the reaction energies of alkaline water splitting on AT‐COF, Ir‐SAS/AT‐COF, Ru SAS/AT‐COF, and IrRu DAS/AT‐COF. (o) The proposed mechanism for the accelerated alkaline water splitting on IrRu DAS/AT‐COF.

Then the DFT calculations were further employed to gain insights into the giant MASE of IrRu DAS/AT‐COF for the superior HER and OER performance. Figure 6a–d depicts the electronic distributions of anti‐bonding and bonding orbitals near the Fermi level (E_f_). For AT‐COF, the bonding orbitals are broadly distributed in the lattice of AT‐COF with relatively weak orbital couplings, which cannot provide efficient electron transfer during the catalytic process (Figure 6a). For both Ir SAS/AT‐COF and Ru SAS/AT‐COF, the metal sites have dominated the anti‐bonding orbitals with significantly improved orbital coupling, indicating the single metal sites being the main active sites for catalytic reaction (Figure 6b,c). Similarly, the intense coupling between anti‐bonding and bonding orbitals is observed in both Ir_SA_ and Ru_SA_ sites in the IrRu DAS/AT‐COF (Figure 6d) [60]. The introduction of Ir_SA_ and Ru_SA_ atoms does not affect the structural stability, and the evident rotations of functional groups with similar angles and structures are noted in these catalysts, resulting in their similar structures.

To obtain in‐depth knowledge of the electronic structures, the projected partial density of states (PDOSs) have been compared. For the pristine AT‐COF, the s,p orbitals of N sites mainly contribute to the conduction band minimum (CBM) and valence band maximum (VBM). The evident gap between CBM and VBM largely limits the electron transfer and lowers the electrical conductivity of AT‐COF (Figure 6e). However, the electronic structures of metalized COFs are evidently modulated after the metal sites are introduced. For Ir SAS/AT‐COF, Ir‐5d orbitals exhibit high electron density near E_F_ as the main active sites for electron transfer toward the intermediates (Figure 6f) [61]. Compared with AT‐COF, the s,p orbitals of N sites have downshifted due to the electron transfer between Ir_SA_ and N sites. For Ru SAS/AT‐COF, we also notice the sharp peak of Ru‐4d orbitals located at E_V_‐0.35 eV (E_V_ = 0 eV) with decreased overlapping with Cl‐s,p orbitals (Figure 6g). The bandgap of Ru SAS/AT‐COF further decreases compared to Ir SAS/AT‐COF, leading to improved electroactivity for water splitting. The giant MASE of IrRu DAS helps IrRu DAS/AT‐COF achieve the optimized electronic structures, where the electron density near E_F_ further increases with reduced bandgap, resulting in the highest electroactivity with the lowest charge transfer resistance (Figure 6h) [62]. In addition, the improved orbital coupling among different atoms enables efficient site‐to‐site electron transfer within IrRu DAS/AT‐COF. As a balance, the d‐band center of IrRu DAS/AT‐COF, located between Ir SAS/AT‐COF and Ru SAS/AT‐COF, guarantees its overall electroactivity and alleviates the overbinding effect to achieve the optimal water splitting performance [11]. Obviously, both the Ir‐5d orbitals in Ir SAS/AT‐COF and IrRu DAS/AT‐COF and Ru‐4d orbitals in Ru SAS/AT‐COF and IrRu DAS/AT‐COF are different from Ir/Ru metal and IrO_2_/RuO_2_, representing the different valence states, in agreement with XAFS results. Notably, due to the electronic modulations of Ru_SA_ sites, Ir‐5d orbitals in IrRu DAS/AT‐COF slightly downshift than Ir SAS/AT‐COF (Figure 6i), while Ru‐4d orbitals display a converse trend to Ir‐5d orbitals and upshift toward E_F_ in the IrRu DAS/AT‐COF (Figure 6j). Such compensating electronic modulation demonstrates the long‐range electron transfer from Ru_SA_ sites to Ir_SA_ sites in the IrRu DAS/AT‐COF matrix, optimizing the overall electronic structure [60, 63], in accordance with the above XPS and operando XAS analysis. The formation of Ir─N and Ru─N bonds has resulted in the downshifted N‐s,p orbitals in the IrRu DAS/AT‐COF, and the s,p orbitals of pyridinic N sites locate in deeper positions, which contribute limitedly to the electroactivity (Figure 6k). Besides, the work functions show a descending order of AT‐COF > Ir SAS/AT‐COF > Ru SAS/AT‐COF > IrRu DAS/AT‐COF, indicating that the lowest energy barrier for electron depletion from IrRu DAS/AT‐COF catalyst to active intermediates, and thus the best electrochemical activity (Figure S53) [64].

As for the thermodynamic trends, AT‐COF has the largest adsorption energy of hydrogen of 0.46 eV, leading to poor water splitting performance (Figure 6l). After anchoring the SAS on COF, the adsorption energies of the proton on Ir_SA_ sites in Ir SAS/AT‐COF and Ru_SA_ sites in Ru SAS/AT‐COF decrease to 0.25 and −0.14 eV, respectively. The very weak and over‐strong ^*^H_ads_ adsorption in Ir SAS/AT‐COF and Ru SAS/AT‐COF are consistent with the d‐band center, and both are not conducive to water splitting [60, 61, 65]. Surprisingly, the ^*^H_ads_ adsorption energies on Ru_SA_ and Ir_SA_ sites in IrRu DAS/AT‐COF are optimized to 0.06 and −0.05 eV, respectively, in which Ir_SA_ are much closer to the neutral line of 0 eV, which suggests that the produced ^*^H_ads_ intermediates during the water dissociation process are preferentially adsorbed at Ir sites, in accordance with operando XFAS results. The synergistic coupling of Ru_SA_ and Ir_SA_ sites endows the optimized kinetics in the desorption of ^*^H_ads_ intermediates, which prevents the very weak and over‐strong ^*^H_ads_ adsorption in corresponding SAC catalysts, thus supporting the optimal HER performance of IrRu DAS/AT‐COF. To supply an efficient HER in an alkaline environment, the IrRu DAS/AT‐COF exhibits the strongest H_2_O adsorption, which is conducive to water activation to generate sufficient protons for water splitting (Figure 6m) [11, 66]. IrRu DAS/AT‐COF shows a dramatically low energy barrier of 0.32 eV for water dissociation compared to Ir SAS/AT‐COF (0.90 eV), Ru SAS/AT‐COF (0.76 eV), and AT‐COF (1.0 eV), suggesting the most efficient ^*^H_ads_ generation on IrRu DAS/AT‐COF (Figure S54) [67]. Therefore, the reaction energy changes during the alkaline hydrogen evolution is obtained, where the H_2_O dissociation step represents the rate‐determining step (RDS) (Figure 6n). AT‐COF exhibits the largest energy barrier of 0.61 eV with an energetically unfavored reaction, leading to poor hydrogen evolution activity. However, IrRu DAS/AT‐COF delivers a much lower energy barrier of RDS (0.06 eV), ensuring the best water splitting performance. Meanwhile, although Ir SAS/AT‐COF and Ru SAS/AT‐COF show similar overall reaction energies with IrRu DAS/AT‐COF, their energy barriers of RDS are much higher than that of IrRu DAS/AT‐COF.

Generally, four elementary reaction steps are involved in the OER process, and these include different intermediates (^*^OH, ^*^O, and ^*^OOH, ^*^ denotes the active sites of the catalyst), the Gibbs free energy profiles are calculated to unveil the origin of enhanced OER activity of the IrRu DAS/AT‐COF. As shown in Figure S55, all the endothermic elementary steps indicate the sluggish reaction kinetics of pristine AT‐COF, in which the conversion of ^*^O to ^*^OOH is the potential RDS with a large energy barrier of 2.51 eV. However, the energy barriers for RDS decreased to 2.40 and 2.19 eV for Ru SAS/AT‐COF and Ir SAS/AT‐COF, respectively after anchoring M SAS on the AT‐COF. More surprisingly, after further simultaneous introduction of Ir_SA_ and Ru_SA_ sites, the remarkably decreased energy barrier for RDS (1.75 eV) is obtained for IrRu DAS/AT‐COF compared to other COF‐based electrocatalysts, which agrees well with the experimental findings, validating the favorable influence of giant DMSE on regulating the electron configuration of M SAS, thereby optimizing the reaction energetics and promoting electrochemical OER activity. Accordingly, based on the above operando and DFT results, we determined the giant MASE of IrRu DAS/AT‐COF for alkaline overall water splitting (Figure 6o), which is achieved through compensating electronic modulation behavior when both Ir_SA_ and Ru_SA_ sites are present in the redox‐active AT‐COF. This unique setting not only supplies the highest electroactivity but also balances the d‐band center to simultaneously optimize the adsorption behavior of H_2_O molecules and various H/O‐containing intermediates in the local environment of IrRu DAS/AT‐COF, thus allowing the dramatic thermodynamic and kinetic cyclic response between the two SAS sites. Therefore, this engineering multiple active sites strategy resulting in improved performance for HER and OER with optimized reaction energetics, thus opening a door to a bifunctional COFs‐based electrocatalyst for highly‐efficient and stable overall water splitting application.

## 3 Conclusions

In summary, this work demonstrates an effective strategy to construct multiple active sites in an electroactive COFs bifunctional electrocatalyst to optimize the local electronic environment for the enhanced water splitting performance. The as‐prepared pyrolysis‐free IrRu DAS/AT‐COFs show superb and stable catalytic activities in 1 m KOH compared to the benchmarks and most recent noble‐metal‐based catalysts. Operando spectroscopy and theoretical calculations reveal that the simultaneous enhanced activity and stability are derived from the giant MASE on the IrRu DAS/AT‐COFs frameworks. The synergetic electron redistribution between Ir_SA_ and Ru_SA_ sites can meet the complex demands of simultaneously supporting the following key events into the local environment, including promoting the adsorption and activation of H_2_O, accelerating the dissociation of H_2_O, optimizing the adsorption behavior of H/O‐containing intermediates, thereby synergistically accelerating the HER and OER kinetics. Furthermore, the donor‐acceptor units and multiple sites enhance electron transport ability and structural stability, showing both conductivity and stability in an alkaline electrolyte. This multifunctional catalytic site mechanism not only provides new insights into the design of bifunctional COFs for water electrolysis at the atomic level but also paves avenues for further exploration of electrocatalysts for other energy conversion applications.

## Author Contributions

M.L. conceived and directed the project. L.R. designed the experiments, analyzed the data, and wrote the paper. Y.X. conducted the synthesis and structural characterization of catalysts. Y.Z. and J.H. contributed to electrochemical performance tests and analyzed the data. M.S. and B.H. contributed to DFT theoretical calculations and data analysis. J.Z., Y.Z., B.R., C.Z., K.C., Q.T., and H.L. supported the partial characterizations and analysis of catalysts. All the authors discussed and commented on the manuscript. L.R., Y. X., Y. Z., and J. Z. have contributed equally.

## Conflicts of Interest

The authors declare no conflicts of interest.

## Supporting information




**Supporting file**: smll72280‐sup‐0001‐SuppMat.docm.

## Data Availability

The data supporting this article have been included as part of the Supplementary Information.
