# Supplementary material for: Engineering Iridium–Ruthenium Dual‐Atomic Active Sites on Redox‐Active Covalent Organic Frameworks for Boosted Overall Water Splitting
Source: Small. 2026 Jan 8;22(13):e10427. doi: 10.1002/smll.202510427 (PMC12954382; doi:10.1002/smll.202510427)
Supplement: Supplementary file 1 — Supporting file: smll72280‐sup‐0001‐SuppMat.docm. [file SMLL-22-e10427-s001.docm]

**Supporting information**

**Engineering Iridium-Ruthenium Dual-Atomic** **Active Sites on Redox-Active Covalent Organic Frameworks for Boosted Overall Water Splitting**

*Lei Ran^a†^, Yifan Xu^a†^,* *Yue Zhang^b†^,* *Jinsong Zhou^a†^, Mingzi Sun**^d^, Yingguang Zhang^a^, Bei Ran^a^, Chengxu Zhang^b^, Jue Hu^b^*, Bolong Huang^c^* and Michael K. H. Leung^a,e^**

^a^ Ability R&D Energy Research Centre, School of Energy and Environment, City University of Hong Kong, Hong Kong, China

^b^ Faculty of Metallurgical and Energy Engineering, Kunming University of Science and Technology, Kunming, China

^c^ Department of Chemistry, City University of Hong Kong, Hong Kong, China

^d^ Department of Applied Biology and Chemical Technology, The Hong Kong Polytechnic University, Hong Kong, China

^e^ State Key Laboratory of Marine Pollution, City University of Hong Kong, Hong Kong, China

†The authors have contributed equally.

Email: [mkh.leung@cityu.edu.hk](mailto:mkh.leung@cityu.edu.hk); hujue@kust.edu.cn; bolhuang@cityu.edu.hk

**Table of Contents**

**1. Experimental Section**

**1.1** Chemicals

**1.2** Fabrication of 4,4′4″-(1,3,5-Triazine-2,4,6-Triyl)trianiline Monomers (TAPT)

**1.3** Fabrication of AT-COF

**1.4** Fabrication of IrRu DAS/AT-COF

**1.5** Materials Characterization

**1.6** Electrochemical Measurements

**1.7** In Situ Raman Characterization

**1.8** DFT Calculation

**2.** **Supplementary Figures and Tables**

**Figure S1** The digital photograph of (a) AT-COF and (b) IrRu DAS/AT-COF.

**Figure S2**The [schematic](javascript:;) [diagram](javascript:;) of single framework in (a) AT-COF and (b) IrRu DAS/AT-COF.

**Figure S3** The [schematic](javascript:;) [diagram](javascript:;) of synthesis process for Ir SAS/AT-COF.

**Figure S4** The [schematic](javascript:;) [diagram](javascript:;) of synthesis process for Ru SAS/AT-COF.

**Figure S5** The PXRD pattern and structure of Ir SAS/AT-COF.

**Figure S6** The PXRD pattern and structure of Ru SAS/AT-COF.

**Figure S7** (a) FESEM image, (b) EDS spectrum, and (c-h) elements mapping of IrRu DAS/AT-COF.

**Figure S8** (a) FESEM image, (b) EDS spectrum, and (c,d) elements mapping of AT-COF.

**Figure S9** The HRTEM images of IrRu DAS/AT-COF.

**Figure S10** The SAED pattern of IrRu DAS/AT-COF.

**Figure S11** (a) The TEM image and (b-d) element mappings of AT-COF.

**Figure S12** (a) SEM image, (b) EDS spectrum, and (c-f) elements mapping of Ir SAS/AT-COF.

**Figure S13** (a) The TEM image and (b-f) element mappings of Ir SAS/AT-COF.

**Figure S14** (a) SEM image, (b) EDS spectrum, and (c-f) elements mapping of Ru SAS/AT-COF.

**Figure S15** (a) The TEM image and (b-f) element mappings of Ru SAS/AT-COF.

**Figure S16** (a) AC-HAADF-STEM image, (b-f) elements mapping, (g) EDS spectrum and (h) schematic model of Ir SAS/AT-COF.

**Figure S17** (a) AC-HAADF-STEM image, (b-f) elements mapping, (g) EDS spectrum and (h) schematic model of Ru SAS/AT-COF.

**Figure S18** The pore size distribution curves of the AT-COF and IrRu DAS/ AT-COF.

**Figure S19** The FTIR spectra of IrRu DAS/AT-COF, Ir SAS/AT-COF, Ru SAS/AT-COF and AT-COF.

**Figure S20** The Raman spectra of IrRu DAS/AT-COF and AT-COF.

**Figure S21** (a) ^13^C NMR spectrum and (b) ^1^H NMR spectrum of AT-COF.

**Figure S22** PXRD patterns of IrRu DAS/AT-COF after treated under harsh condition.

**Figure S23** The TGA curves of IrRu DAS/AT-COF, Ir SAS/AT-COF, Ru SAS/AT-COF and AT-COF.

**Figure S24** The contact angle values of (a) IrRu DAS/AT-COF, (b) Ru SAS/AT-COF, (c) Ir SAS/AT-COF and (d) AT-COF.

**Figure S25** The TPD-H_2_ curves of IrRu DAS/AT-COF, Ir SAS/AT-COF, Ru SAS/AT-COF and AT-COF.

**Figure S26** The full range survey XPS spectra of (a) IrRu DAS/AT-COF and (b) AT-COF.

**Figure S27** (a) The full range survey and (b) high-resolution N 1s XPS spectra of Ir SAS/AT-COF.

**Figure S28** (a) The full range survey and (b) high-resolution N 1s XPS spectra of Ru SAS/AT-COF.

**Figure S29** The EXAFS fitting curves of IrRu DAS/AT-COF in k space

**Figure S30** (a) overpotentials curves and (b) the catalytic performance comparison of IrRu DAS/AT-COF with other noble-metal based HER catalysts.

**Figure S31** The exchange current density plots of IrRu DAS/AT-COF, Ir SAS/AT-COF, Ru SAS/AT-COF, AT-COF, and commercial Pt/C in 1.0 M KOH.

**Figure S32** (a-e) The CV curves at various scan rates and (f) C_dl_ plots of IrRu DAS/AT-COF, Ir SAS/AT-COF, Ru SAS/AT-COF, AT-COF, and commercial Pt/C in 1.0 M KOH.

**Figure S33** The geometric current density was normalized by ECSA of IrRu DAS/AT-COF, Ir SAS/AT-COF and Ru SAS/AT-COF in 1.0 M KOH.

**Figure S34** (a) FESEM and (b-h) EDS mapping images of IrRu DAS/AT-COF after 100 h HER test.

**Figure S35** (a) TEM, (b) HRTEM images, (c-h) element mappings and (i) EDS spectrum of IrRu DAS/AT-COF after 100 h HER stability test in 1M KOH.

**Figure S36** (a) AC-HAADF-STEM image, (b-g) elements mapping and (h) EDS spectrum of IrRu DAS/AT-COF.

**Figure S37** The PXRD patterns of IrRu DAS/AT-COF after 100 h HER stability test.

**Figure S38** (a) The full range survey, (b) high-resolution N 1s, (c) high-resolution Ru 3p and (d) high-resolution Ir 4f XPS spectra of IrRu DAS/AT-COF after 100 h HER stability test.

**Figure S39** (a) overpotentials curves and (b) the catalytic performance comparison of IrRu DAS/AT-COF with other noble-metal based OER catalysts.

**Figure S40** The Tafel plots of IrRu DAS/AT-COF, Ir SAS/AT-COF, Ru SAS/AT-COF, AT-COF and RuO_2_ for OER in 1 M KOH.

**Figure S41** The CV curves of (a) IrRu DAS/AT-COF, (b) Ru SAS/AT-COF, (c) Ir SAS/AT-COF, (d) AT-COF, and (e) commercial RuO_2_ in 1.0 M KOH.

**Figure S42** (a) XRD pattern, (b) TEM image, (c-g) element mappings and (h) EDS spectrum of IrRu DAS/AT-COF after 100 h OER stability test in 1 M KOH.

**Figure S43** The LSV curves of Pt/C||RuO_2_ system before and after 20 h overall water splitting test in 1M KOH.

**Figure S44** The long-term plots of IrRu DAS/AT-COF‖IrRu DAS/AT-COF cell at 100 mA cm^-2^.

**Figure S45** The catalytic performance comparison of IrRu DAS/AT-COF with other catalysts for alkaline overall water splitting.

**Figure S46** The Re(k^3^χ(k)) oscillations of (a) Ir L_3_-edge operando EXAFS and (b) Ru *K*-edge operando EXAFS.

**Figure S47** The wavelet transform of k^3^-weighted in-situ EXAFS at Ir L_3_-edge of IrRu DAS/AT-COF at different applied potentials for alkaline HER.

**Figure S48** The wavelet transform of k^3^-weighted in-situ EXAFS at Ru *K*-edge of IrRu DAS/AT-COF at different applied potentials for alkaline HER.

**Figure S49** The R-space curve-fitting of EXAFS spectra at the Ir L_3_-edge of the IrRu DAS/AT-COF (a) under open circuit condition and (c) at -0.1 V vs. RHE, respectively. The corresponding Re(k^3^χ(k)) oscillations of different fitting paths of the IrRu DAS/AT-COF (b) under open circuit condition and (d) at -0.1 V vs. RHE, respectively.

**Figure S50** The R-space curve-fitting of EXAFS spectra at the Ru K-edge of the IrRu DAS/AT-COF (a) under open circuit condition and (c) at -0.1 V vs. RHE, respectively. The corresponding Re(k^3^χ(k)) oscillations of different fitting paths of the IrRu DAS/AT-COF(b) under open circuit condition and (d) at -0.1 V vs. RHE, respectively.

**Figure S51** The work function comparison of IrRu DAS/AT-COF, Ir SAS/AT-COF, Ru SAS/AT-COF and AT-COF.

**Figure S52** The energy costs for water dissociation of IrRu DAS/AT-COF, Ir SAS/AT-COF, Ru SAS/AT-COF and AT-COF.

**Figure S53** Calculated free energy diagram for OER at potential U = 0 V versus RHE of IrRu DAS/AT-COF, Ir SAS/AT-COF, Ru SAS/AT-COF and AT-COF.

**Table S1.** Fractional atomic coordinates for the unit cell of IrRu DAS/AT-COF.

**Table S2.** Fractional atomic coordinates for the unit cell of AT-COF.

**Table S3.** ICP-AES analysis results of different IrRu DAS/AT-COF.

**Table S4**. Textural parameters of IrRu DAS/AT-COF and AT-COF.

**Table S5.** Structural parameters of the catalysts obtained from EXAFS fitting.

**Table S6.** HER performance comparison of the IrRu DAS/AT-COF with other state-of-the-art noble-metal-based electrocatalysts reported previously in 1 M KOH.

**Table S7.** OER performance comparison of the IrRu DAS/AT-COF with other state-of-the-art noble-metal-based electrocatalysts reported previously in 1 M KOH.

**Table S8.** Performance comparison of the IrRu DAS/AT-COF‖IrRu DAS/AT-COF with other previously reported state-of-the-art catalysts for overall water splitting in 1M KOH.

**Table S9.** Structural parameters extracted from the quantitative EXAFS curve-fitting under operando conditions.

**1. Experimental Section**

**1.1 Chemicals**

Trifluoromethanesulfonic acid (CF_3_SO_3_H, 99%), 4-aminobenzonitrile (C_7_H_6_N_2_, 99%), 1,2-acenaphthenequinone (C_12_H_6_O_2_, 98.0%), dioxane (C_4_H_8_O_2_, 99.9%), acetonitrile (C_2_H_3_N, 99.5%), acetic acid (C_2_H_4_O_2_, 98%), tetrahydrofuran (C_4_H_8_O, 99%), N,N-dimethylformamide (C_3_H_7_NO), methanol (CH_4_O, 99%), RuCl_3_·xH_2_O (99%), IrCl_3_·xH_2_O (99%) were purchased from Aladdin Chemistry Co., Ltd. All the materials were of analytical grade and used without further purification, absolute ethanol, [acetone](javascript:;) and deionized water with a resistivity of 18.25 MΩ cm^-1^ were used throughout the experiments.

**1.2 Fabrication of 4,4′4″-(1,3,5-Triazine-2,4,6-Triyl)trianiline Monomers (TAPT)**

1.544 g (13.076 mmol) of 4-aminobenzonitrile was placed in a round-bottom flask on an ice bath at first. Then, 4 mL (44.4 mmol) of trifluoromethanesulfonic acid was added dropwise very slowly to maintain a temperature of 0 °C. After 1 h, the resultant slurry was stirred for 24 h at room temperature in an inert N_2_ atmosphere. After completion of the reaction, 20 mL of distilled water was poured into the mixture, followed by neutralization after adding 2 M NaOH solution until a final pH~7 was obtained. The pH level was monitored experimentally using litmus paper. The resultant yellow solid product was filtered off using simple filtration techniques and washed with distilled water five times to remove additional NaOH.

**1.3 Fabrication of AT-COF**

AT-COF was first synthesized via our previous report.^1^ Typically, TAPT (0.1 mmol) and 1,2-acenaphthenequinone (0.15 mmol) was added in the dioxane/acetonitrile/acetic acid (0.5 mL, 0.5 mL, 0.1 mL) mixture in a 10 mL Pyrex tube. The Pyrex tube was placed into an ultrasonic bath for 15 min and then degassed via three freeze-pump-thaw cycles. Finally, the tube was flame-sealed and heated at 120 °C for 72 hours. The precipitate was collected through filtration, washed with acetone and tetrahydrofuran, and stirred in N,N-dimethylformamide for 3 hours to remove all unreacted monomers. Afterward, the powder was collected and dried at 120 °C under vacuum overnight to produce AT-COF as a dark-red solid in 88% isolated yield.

**1.4 Fabrication of IrRu DAS/AT-COF**

IrRu DAS/AT-COF loaded with dual-metal ions was obtained by adding metal resources into AT-COF/methanol dispersion. Briefly, 50 mg of AT-COF was [impregnat](javascript:;)ed with IrCl_3_·6H_2_O (15 mg) and RuCl_3_·3H_2_O (15 mg) in methanol and stirred overnight under ambient conditions. Finally, the powders were collected, washed, and dried under vacuum overnight to obtain IrRu DAS/AT-COF. M SAS/AT-COF (M=Ir and Ru) can be obtained through the same method except for the metal resource.

**1.5 Materials Characterization**

Powder X-ray diffraction (PXRD) patterns were [teste](D:/%E8%BD%AF%E4%BB%B6/Dict/8.9.3.0/resultui/html/index.html#/javascript:;)d by X-ray diffractometer equipped with graphite monochromatized Cu K_α_ radiation (λ = 1.54178 Å). SEM images were captured by a field-emission scanning electron microscope (SEM, FEI Nova Nano SEM 450). ^1^H-NMR spectra were recorded at 600 MHz frequency using a JEOL Varian Inova 600 MHz spectrometer. ^13^C-NMR spectrum was recorded in the same NMR spectrometer at the frequency of 150 MHz. TEM images were performed on transmission electron microscopy (TEM, FEI TF30) with selected area electron diffraction (SAED). Aberration-corrected HAADF-STEM images were obtained on Titan Cubed Themis Z 60-300 kV electron microscopy with electron energy loss spectroscopy (EELS). FTIR spectra were performed by a Thermo Fisher Nicolet iS5. N_2_ adsorption-desorption curves were obtained by Micromeritics ASAP 2460 surface areas and porosities [profiler](D:/%E8%BD%AF%E4%BB%B6/Dict/8.9.3.0/resultui/html/index.html#/javascript:;). Specific surface area was acquired via Brunauer-Emmett-Teller (BET) approach. X-ray photoelectron spectroscopy (XPS, ESCALAB 250) pattern was applied to explore the elements composition and valence states of materials. ICP-MS measurements were characterized by Agilent 7700s. Thermogravimetric analysis (TGA) was performed on a Netzsch STA 449 F3 Jupiter within a temperature range of 25-800 °C in N_2_ with a heating rate of 10 °C/min. X-ray absorption spectroscopy (XAS) was obtained at BL14W beamline in Shanghai Synchrotron Radiation Facility (SSRF, operated at 3.5 GeV with a stable current of 200 mA).

**1.6 Electrochemical Measurements**

The electrochemical measurements were carried out at room temperature using the CHI760e electrochemical workstation (CH Instruments, Inc.) in a standard three-electrode system with Ag/AgCl (saturated KCl), 1 cm 2 Pt plate, and the synthesized catalysts as the reference, counter, and working electrodes, respectively. The HER performance was tested from acidic to alkaline media at the same conditions and in the 1 M KOH solutions as the electrolyte. The Pt/C and IrRu DAS/AT-COF ink were then prepared by dispersing 5 mg of Pt/C (20 wt%) and IrRu DAS/AT-COF catalyst in a 1 mL mixture of water/ethanol (3:1 v/v) and 5 μL of Nafion (5 wt%), respectively. Then, the catalyst ink was coated onto Ni foam to form Pt/C and IrRu DAS/AT-COF electrodes. The polarization curves were obtained using linear sweep voltammetry (LSVs) with a scan rate of 5 mV s ^-1^. The durability test was run by the chronopotentiometry technique with a fixed current density of 100 mA cm^-2^. The electrochemical surface areas (ECSA) of the catalysts were estimated using electrochemical double-layer capacitance (C_dl_). To estimate the C_dl_, cyclic voltammetry (CV) was carried out in the non-faradaic potential region from -0.724 to -0.824 V vs. Ag/AgCl with various scan rates (5 ~ 50 mV s^-1^). The capacitive current is plotted versus scan rate. Electrochemical impedance spectroscopy (EIS) measurements were performed at the overpotential of 70 mV in tne range from 100000 to 0.1 Hz. All the potentials measured versus Ag/AgCl were calibrated to reversible hydrogen electrode (RHE) according to Nernst equation:

$E_{RHE}=E_{Ag/AgCl}+0.059pH+E_{Ag/AgCl}^{0}$

where *E_Ag/AgCl_* was the applied potential vs.Ag/AgCl (which is the applied bias in this work), and another is the standard electrode potential of the Ag/AgCl at 25℃. All three-electrode electrochemical measurements were conducted with full (100%) iR compensation, whereas the two-electrode measurements were performed without iR compensation.

**Calculation of ECSA**

The ECSA value was estimated by using C_dl_ that was obtained from the linear slope of the plot between scan rate and current density measured by cyclic voltammetry (CV) curves in 1.0 M KOH solution. The specific capacitance for a flat surface is generally found to be in the range of 20 ~ 60 μF cm^-2^. Assume that the specific capacitance of a flat surface is 40 μF cm^-2^ for 1 cm^2^ of real surface area. ECSA was calculated by the following equation:

$ECSA=\frac{Specific capacitance (mF {cm}^{-2})}{60 mF {cm}^{-2} per {cm}^{2}}$

**Calculation of TOF**

The TOF is evaluated using the following equation:

$TOF=\frac{j * A}{2Fm}$

where j is current density (A cm^-2^ = C s^-1^ cm^-2^ ) at a specific overpotential (-0.2 ~ 0.6 V vs. RHE), A is surface area of electrode (cm^2^), the factor 1/2 is related to number of electrons required to generate one molecule, F is Faraday constant (96485.4 C mol^-1^, and m is the number of surfaced active sites (moles) those take part in electrochemical HER.

$m=\frac{QA}{2F}$

Here, the anodic charge (Q_A_, Coulombs) was calculated from the anodic part of CV curves measured in 1 M KOH solution at 50 mV s^-1^.

**1.7 In Situ Raman Characterization**

The in situ Raman spectra were recorded by Renishaw inVia Qontor Raman Spectroscopy (laser wavelength = 532 nm). The electrolytes were prepared with extra care to avoid contaminations from other ions and glassware. The catalyst ink was dropped on a glassy carbon electrode as the working electrode, Pt wire as the counter electrode, and SCE as the reference electrode.

**1.8 DFT Calculation**

In this work, we have used the CASTEP packages to carry out all the DFT calculations on AT-COF, Ir SAS/AT-COF, Ru SAS/AT-COF, and IrRu DAS/AT-COF to reveal their HER performances.^2^ For the functionals, we have selected the generalized gradient approximation (GGA) and Perdew-Burke-Ernzerhof (PBE), which are sufficient to supply accurate descriptions of the exchange-correlation interactions in electrocatalysis.^3-5^ Projector Augmented Wave (PAW) methods were used for the pseudopotentials. The cutoff energy is set to 380 eV, which is generated by default based on the ultrafine quality selection within the ultrasoft pseudopotentials, which are verified by the convergence tests. In this work, we have applied the fine k-point set with 0.04 1/Å separation. Meanwhile, the Broyden-Fletcher-Goldfarb-Shannon (BFGS) algorithm is also applied for all the energy minimizations.^6^ The models of AT-COF, Ir SAS/AT-COF, Ru SAS/AT-COF, and IrRu DAS/AT-COF are constructed based on experimental characterizations.

Considering the model size and calculation efficiency, we constructed one layer of the AT-COF, Ir SAS/AT-COF, Ru SAS/AT-COF, and IrRu DAS/AT-COF structures to investigate surface electroactivity and water splitting performances. A vacuum space of 20 Å was adopted for slab models to allow the full geometry optimizations during the adsorptions of different key intermediates, which also avoids interactions with the periodic cells in the z-axis. We have applied the following convergency criteria for the geometry optimizations including: 1) Hellmann-Feynman forces should not exceed 0.001 eV/Å, and 2) the total energy difference should not be over 5×10^-5^ eV/atom.

**2.** **Supplemental Figures and Tables**


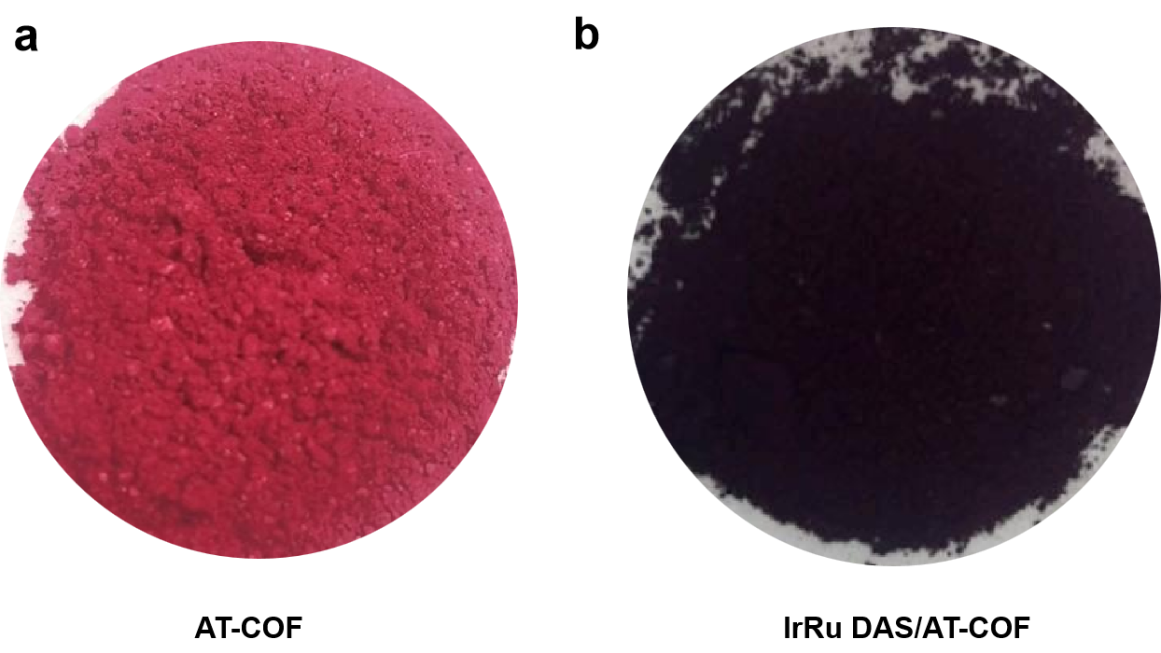


**Figure S1** The digital photograph of (a) AT-COF and (b) IrRu DAS/AT-COF.


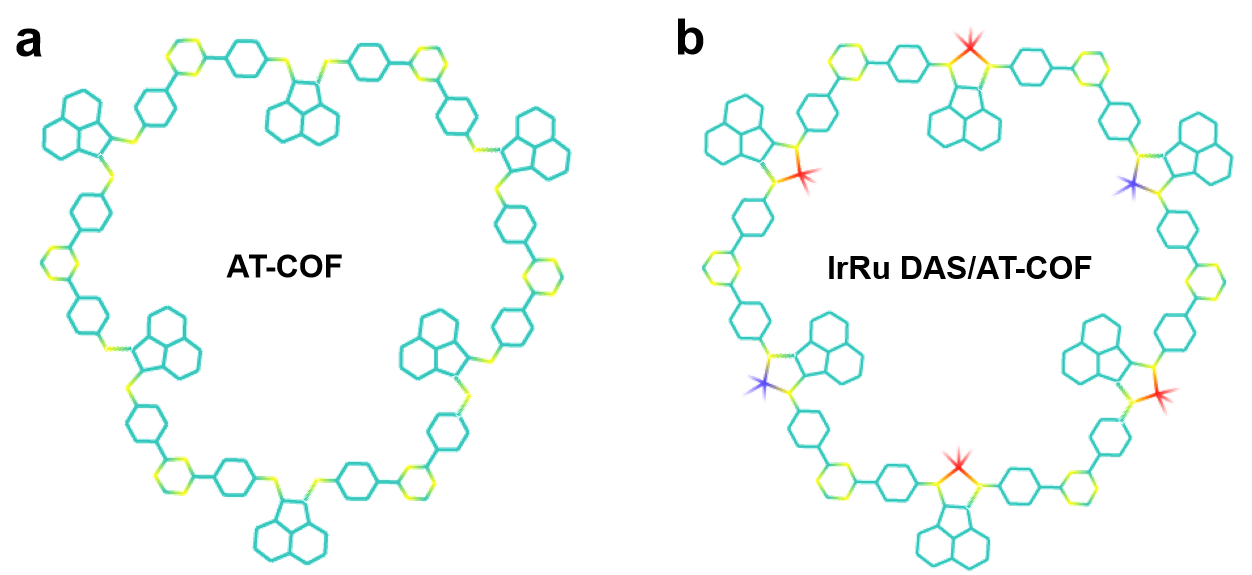


**Figure S2** The [schematic](javascript:;) [diagram](javascript:;) of single framework in (a) AT-COF and (b) IrRu DAS/AT-COF.

**Note:** Notably, the Ir or Ru single atoms (Ir and Ru SAS) can also be anchored on COF using pure Ir or Ru precursors to form Ir SAS/AT-COF (Figure S3) and Ru SAS/AT- COF (Figure S4), which show similar PXRD patterns with IrRu DAS/AT-COF, revealing the same crystalline structure (Figures S5 and S6).


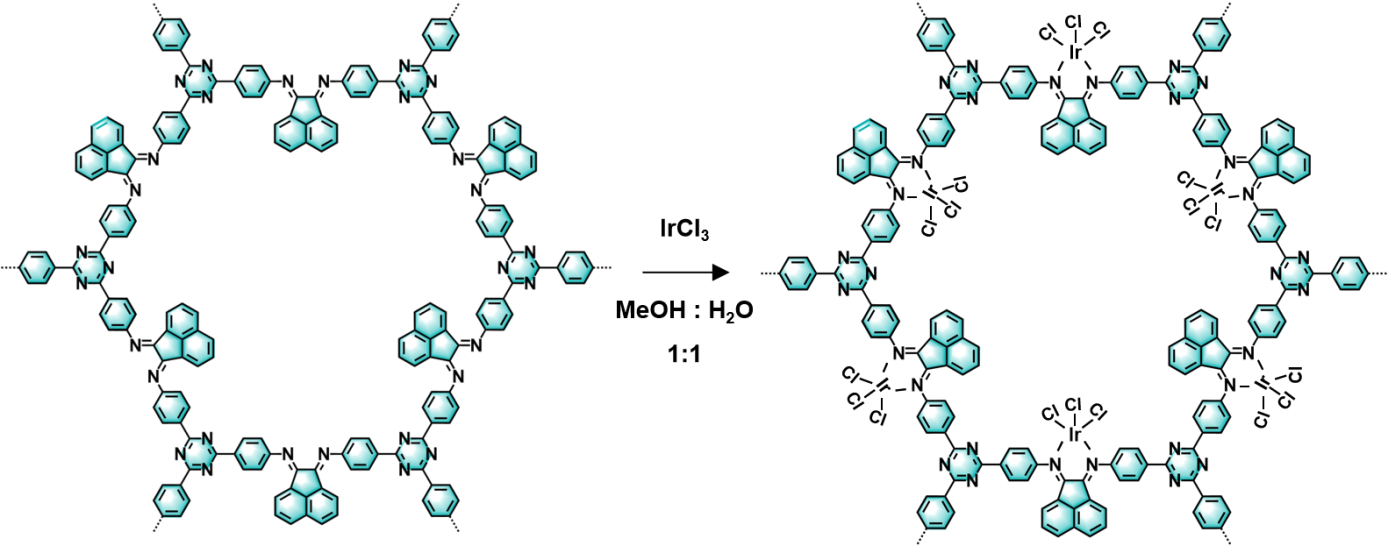


**Figure S3** The [schematic](javascript:;) [diagram](javascript:;) of synthesis process for Ir SAS/AT-COF.


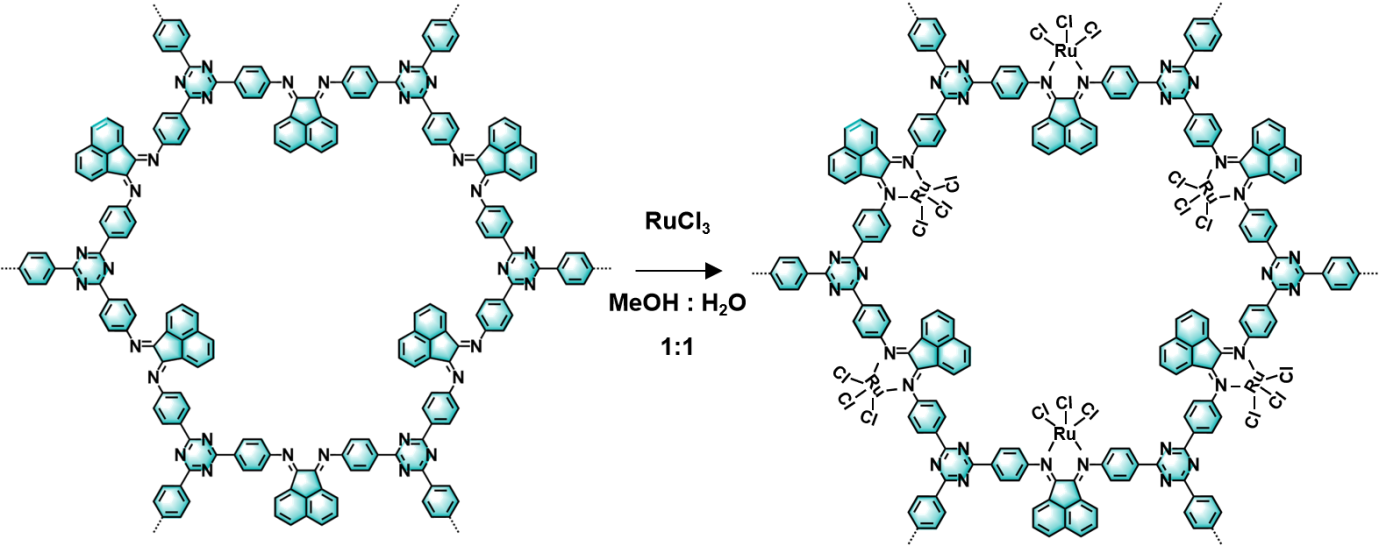


**Figure S4** The [schematic](javascript:;) [diagram](javascript:;) of synthesis process for Ru SAS/AT-COF.


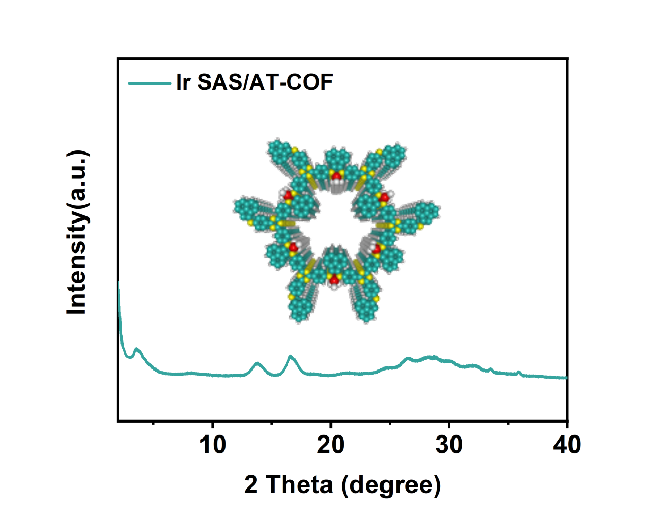


**Figure S5** The PXRD pattern and structure of Ir SAS/AT-COF.


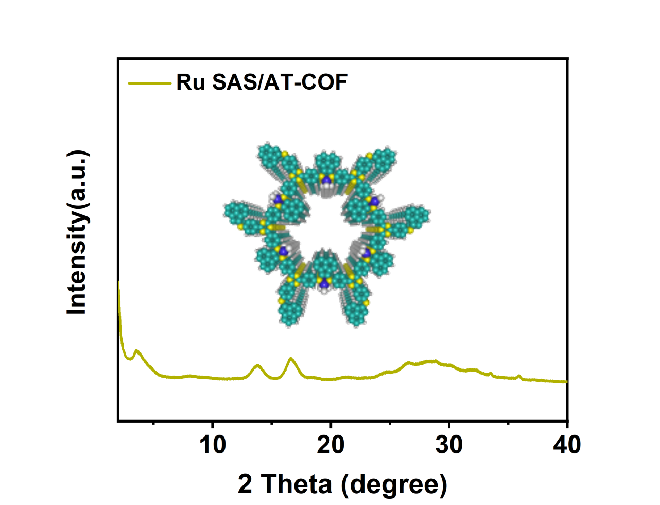


**Figure S6** The PXRD pattern and structure of Ru SAS/AT-COF.


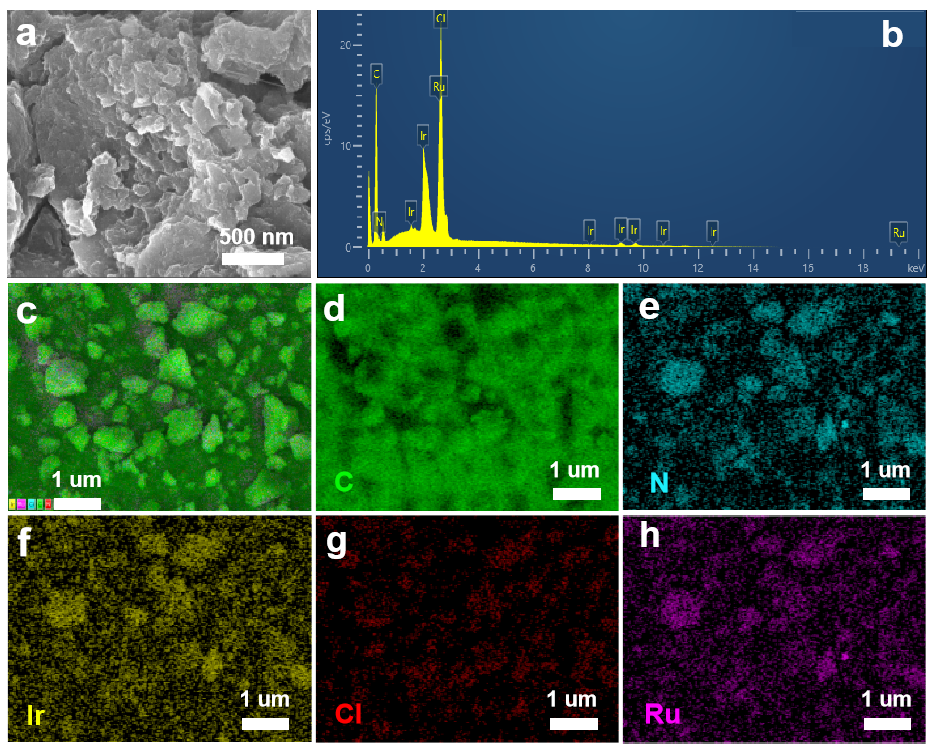


**Figure S7** (a) FESEM image, (b) EDS spectrum, and (c-h) elements mapping of IrRu DAS/AT-COF

.
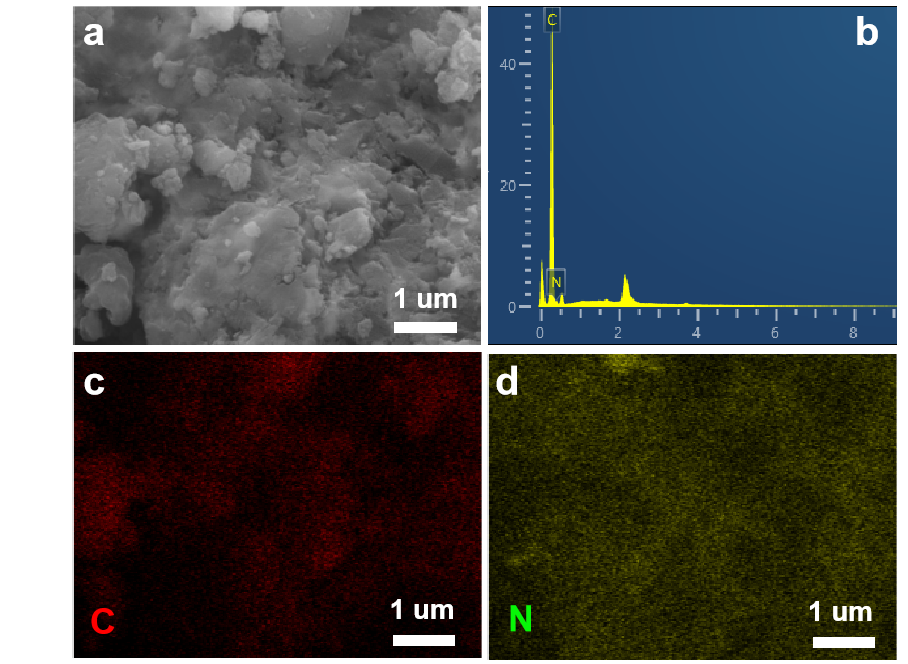


**Figure S8** (a) FESEM image, (b) EDS spectrum, and (c,d) elements mapping of AT-COF.


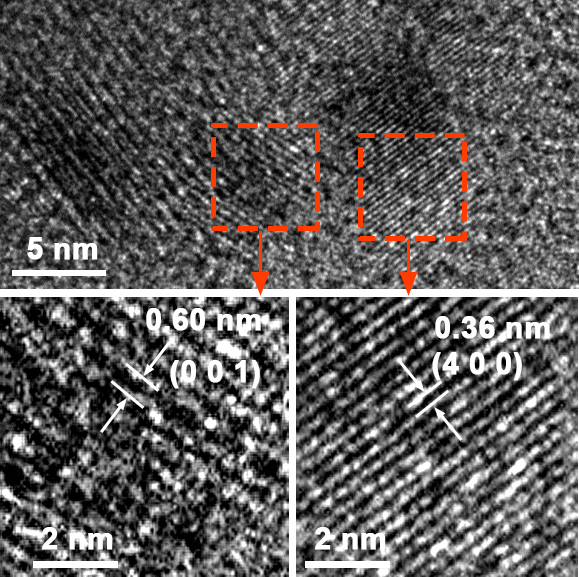


**Figure S9** The HRTEM images of IrRu DAS/AT-COF.


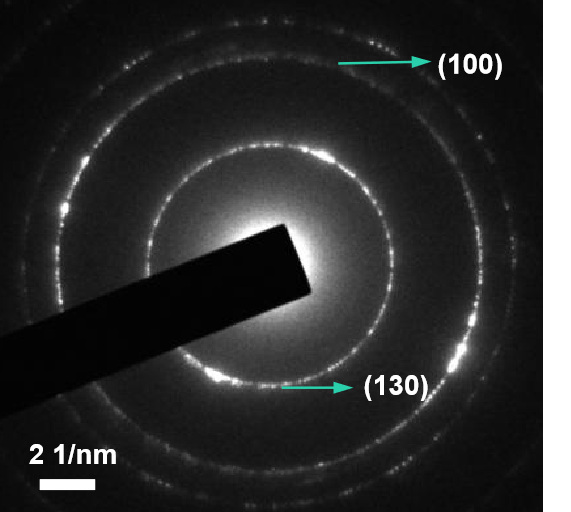


**Figure S10** The SAED pattern of IrRu DAS/AT-COF.


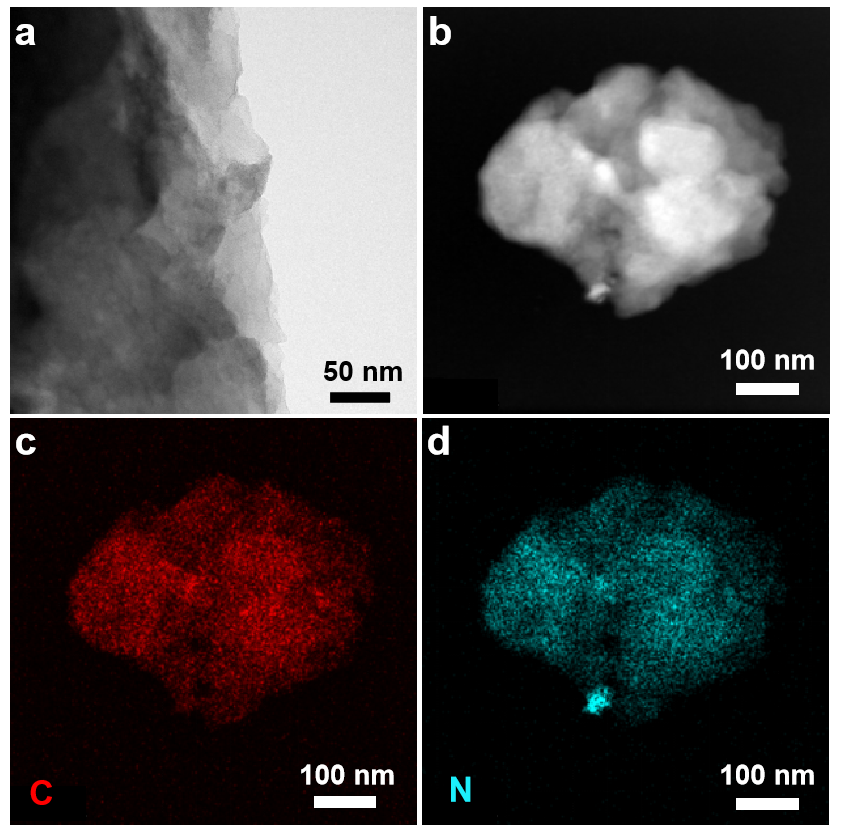


**Figure S11** (a) The TEM image and (b-d) element mappings of AT-COF.

**Note:** For the catalysts with single metal sites, both the Ir SAS/AT-COF (Figures S12 and S13) and Ru SAS/AT-COF (Figures S14 and S15) catalysts show similar surface morphology and uniformly distributed elements. These obvious bright dots in AC-HAADF-STEM also identify the formation of corresponding metal SAS in Ir SAS/AT- COF (Figure S16) and Ru SAS/AT-COF (Figure S17) catalysts.


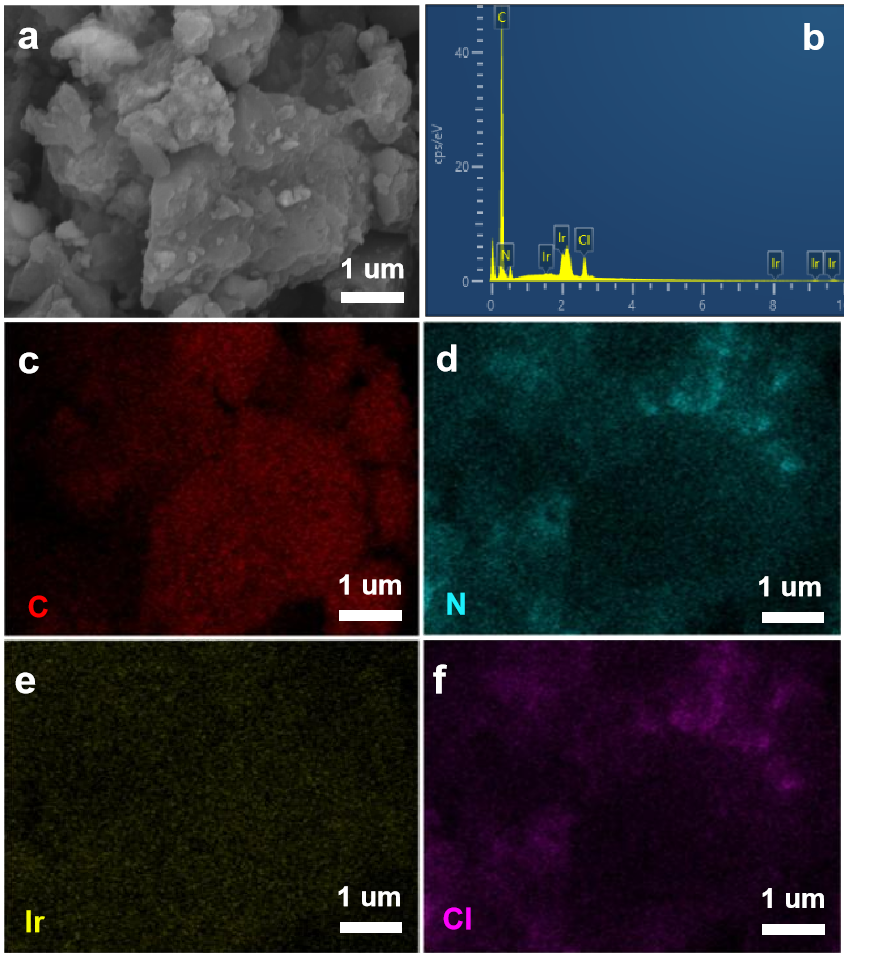


**Figure S12** (a) SEM image, (b) EDS spectrum, and (c-f) elements mapping of Ir SAS/AT-COF.


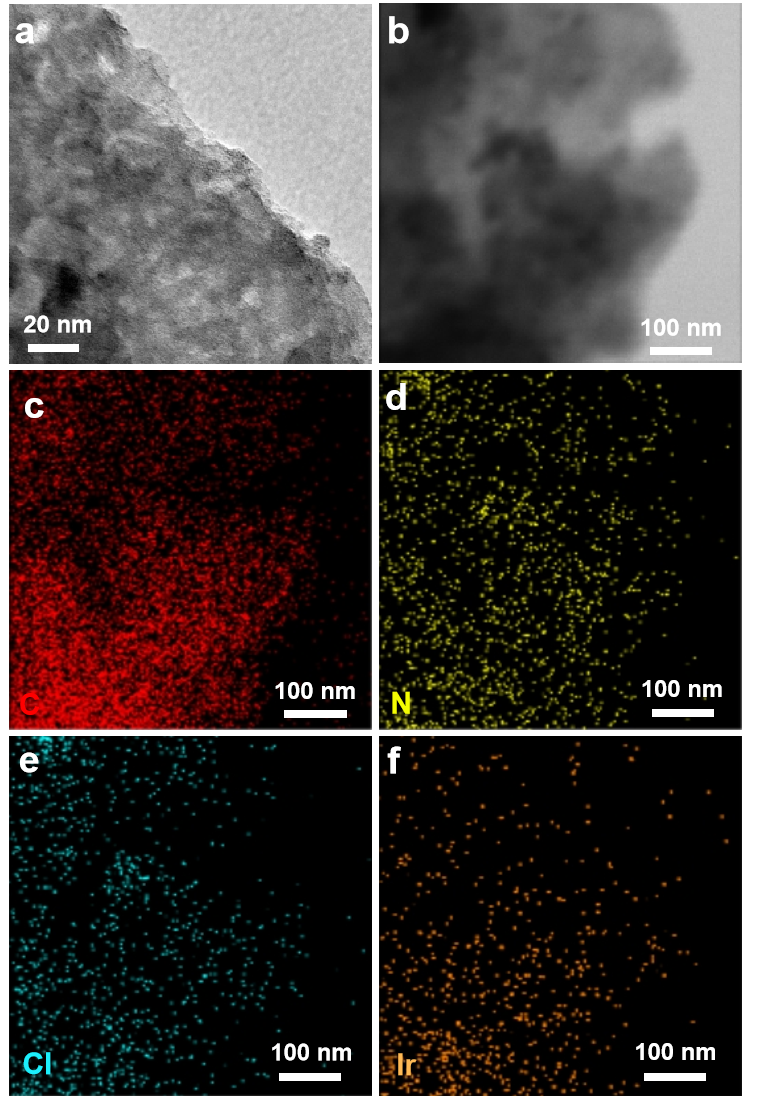


**Figure S13** (a) The TEM image and (b-f) element mappings of Ir SAS/AT-COF.


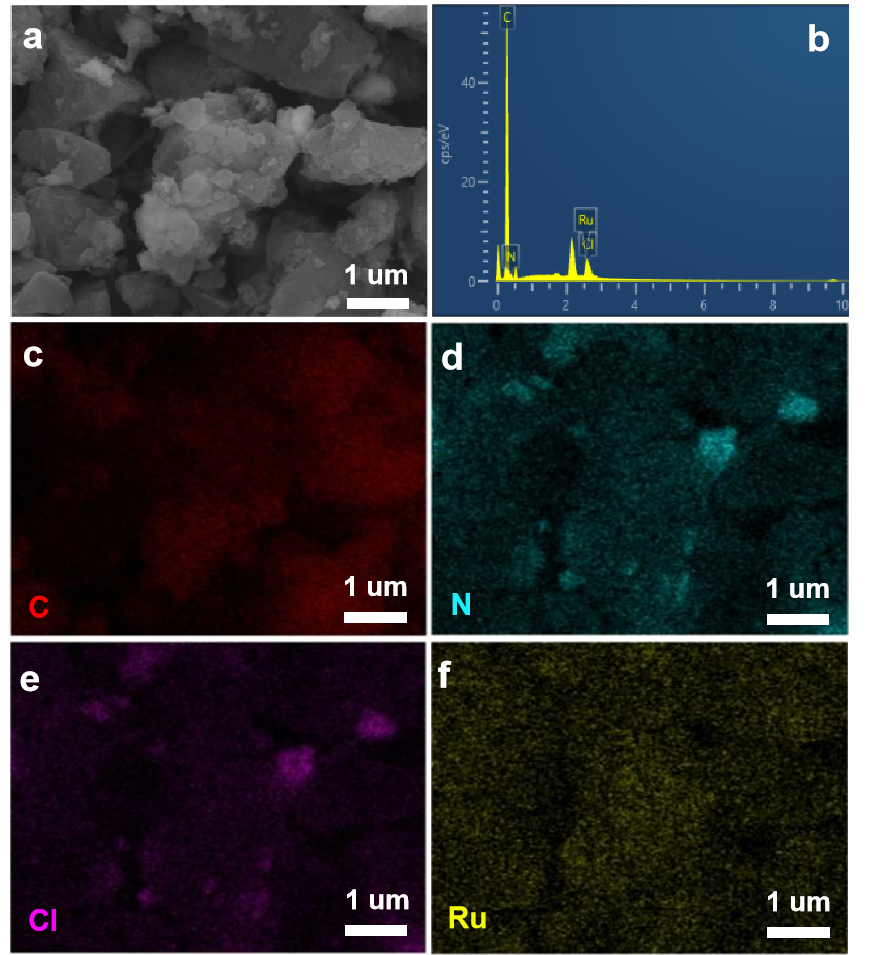


**Figure S14** (a) SEM image, (b) EDS spectrum, and (c-f) elements mapping of Ru SAS/AT-COF.


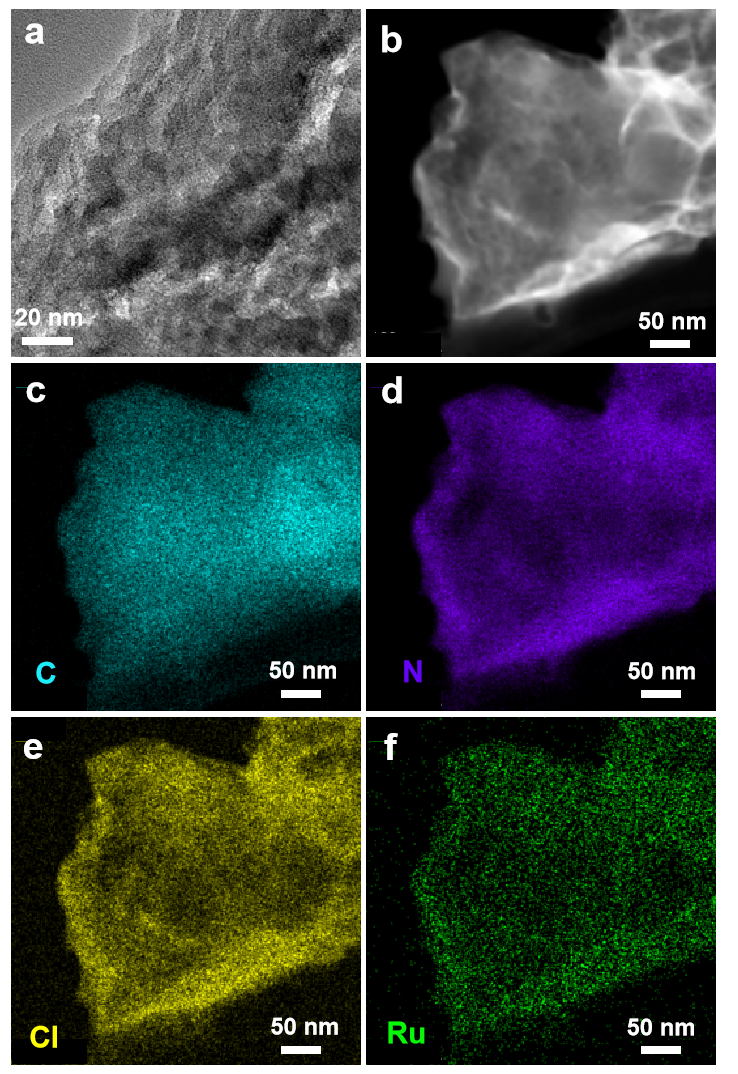


**Figure S15** (a) The TEM image and (b-f) element mappings of Ru SAS/AT-COF.


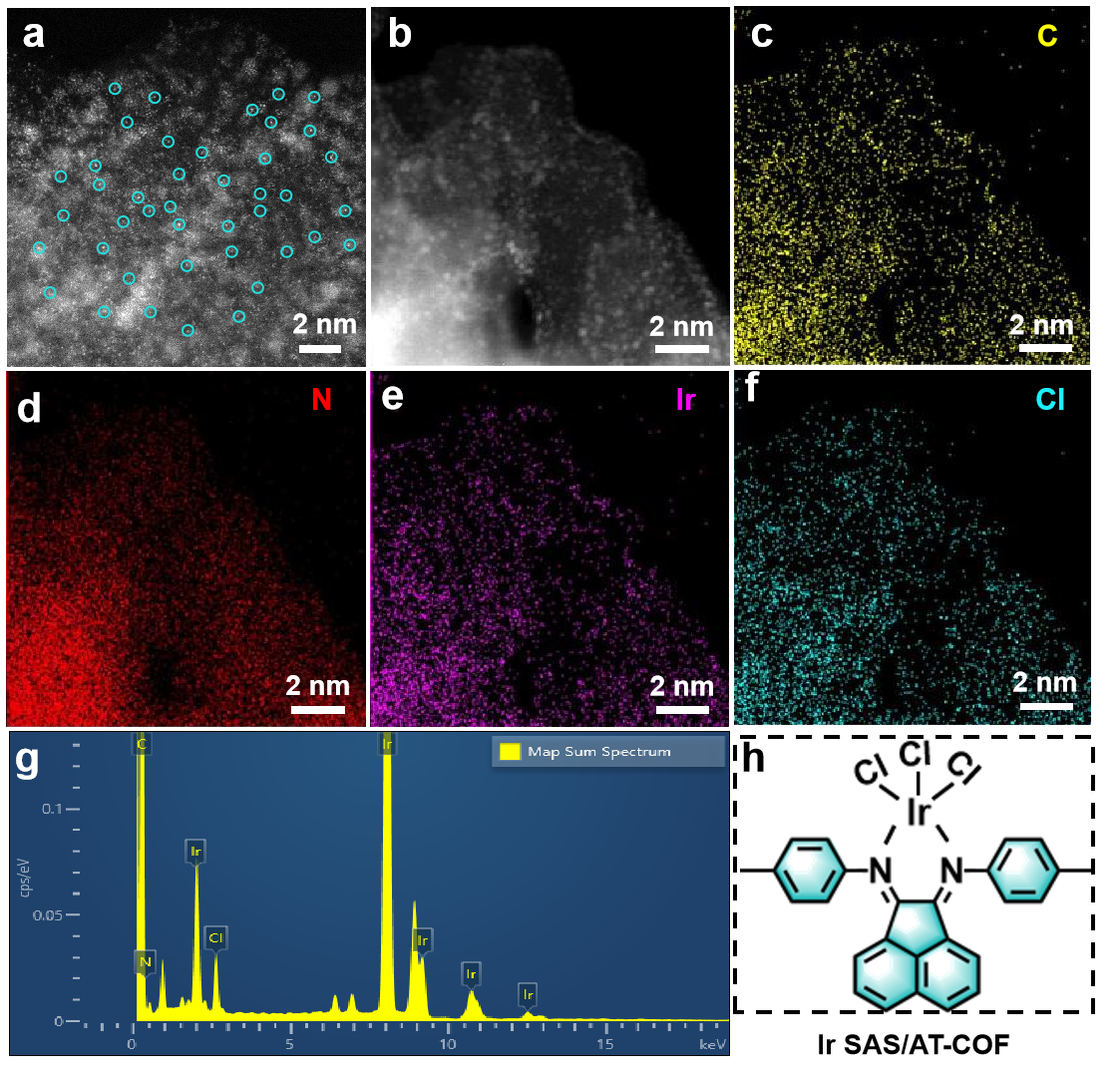


**Figure S16** (a) AC-HAADF-STEM image, (b-f) elements mapping, (g) EDS spectrum and (h) schematic model of Ir SAS/AT-COF.


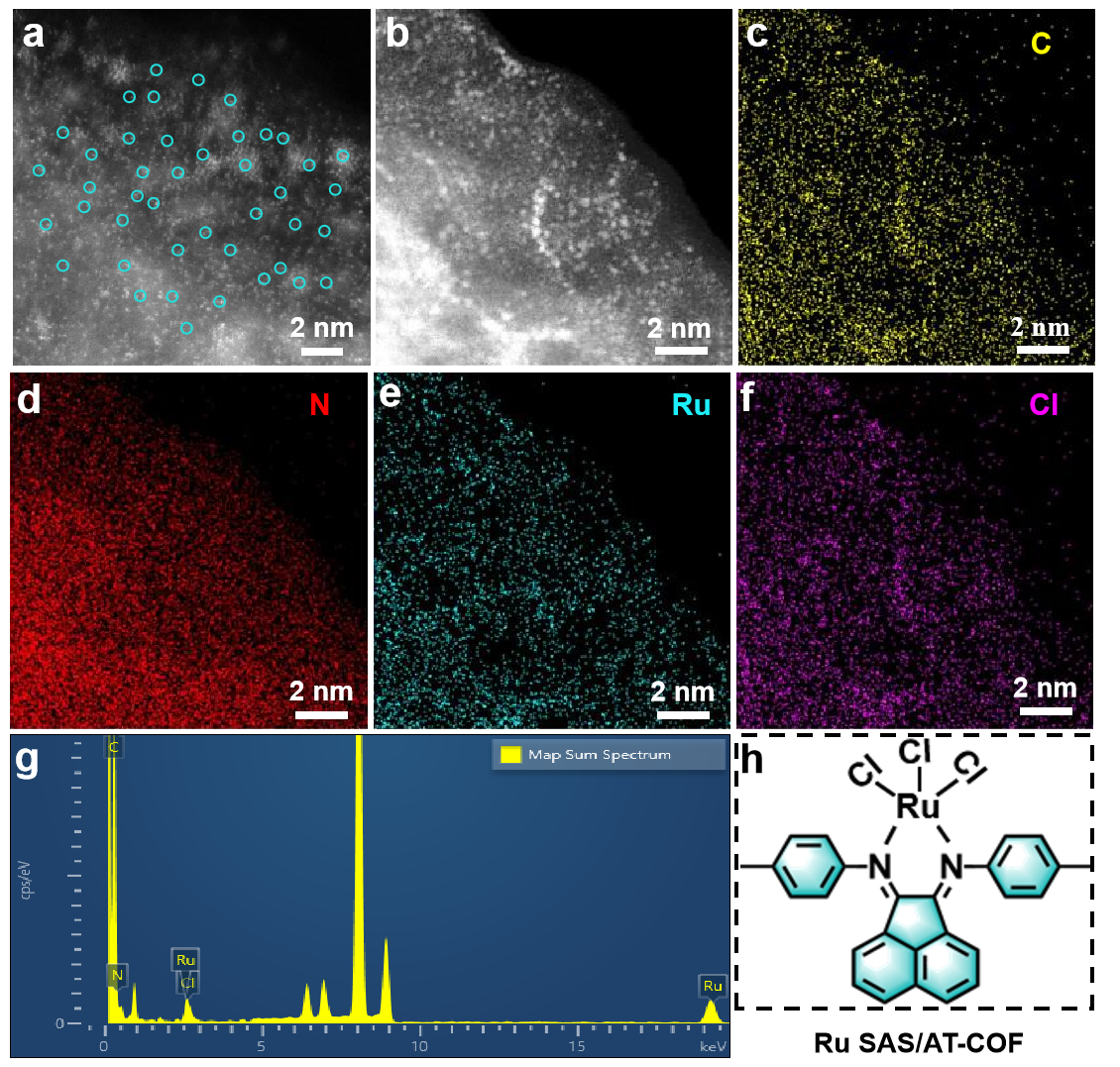


**Figure S17** (a) AC-HAADF-STEM image, (b-f) elements mapping, (g) EDS spectrum and (h) schematic model of Ru SAS/AT-COF.


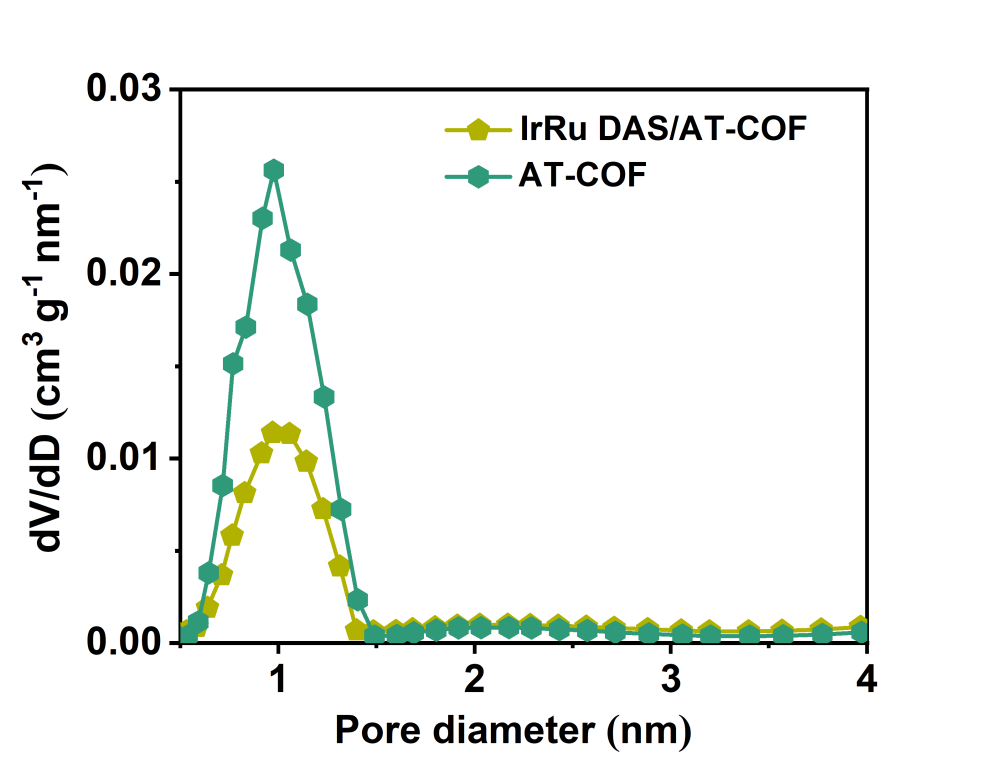


**Figure S18** The pore size distribution curves of the AT-COF and IrRu DAS/ AT-COF.


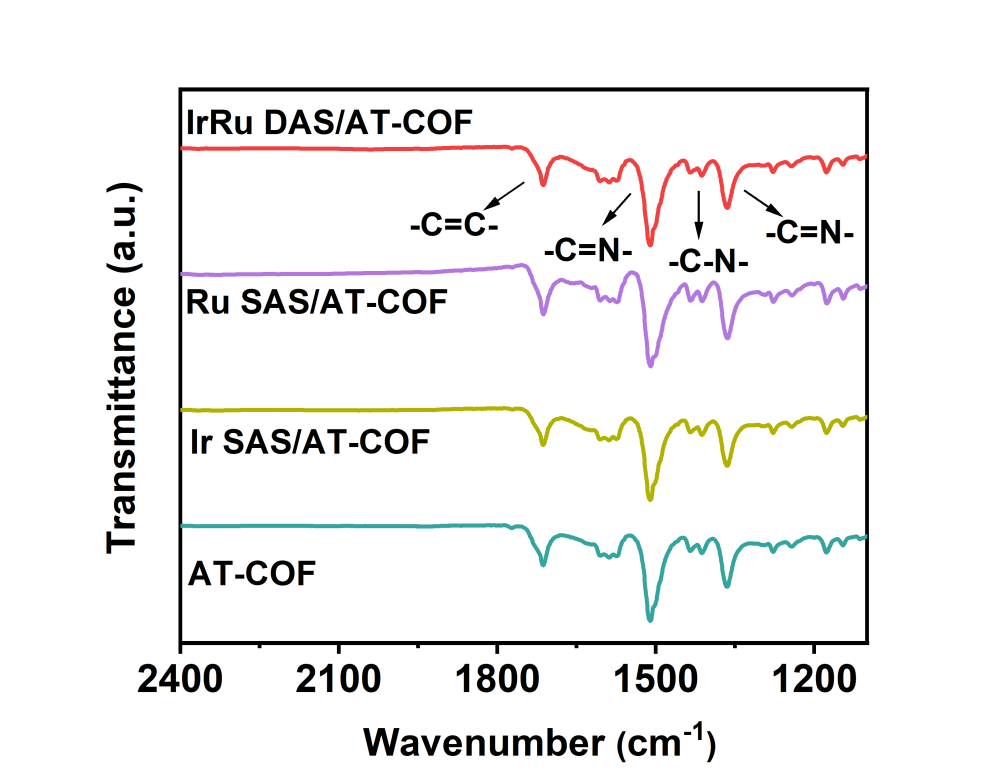


**Figure S19** The FTIR spectra of IrRu DAS/AT-COF, Ir SAS/AT-COF, Ru SAS/AT-COF and AT-COF.


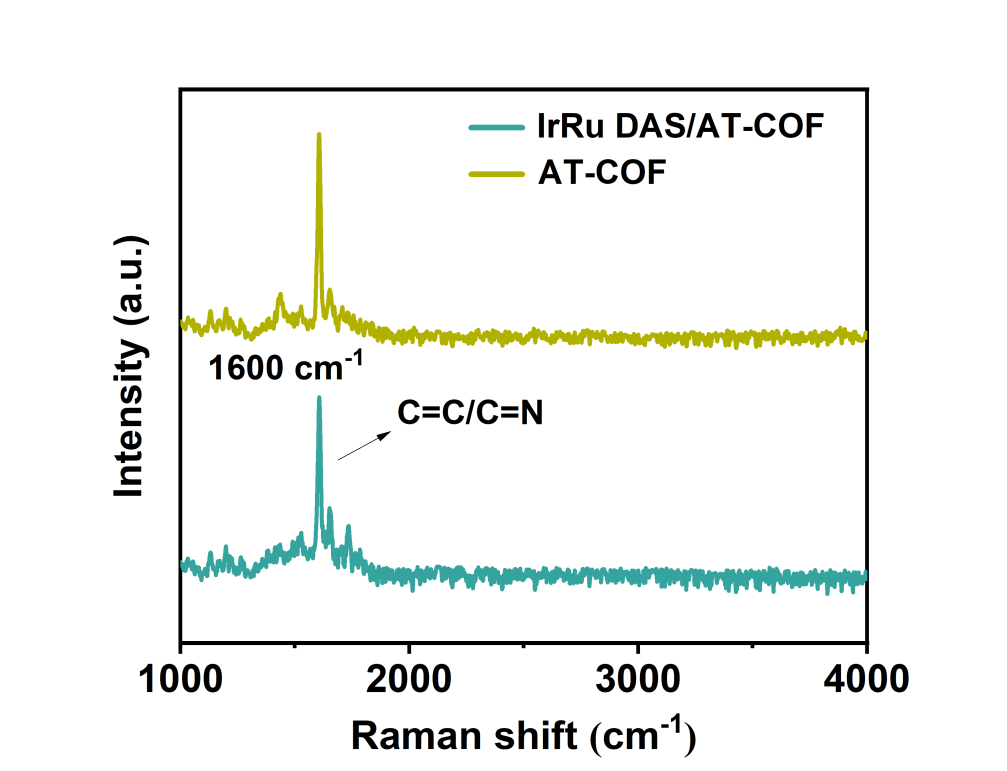


**Figure S20** The Raman spectra of IrRu DAS/AT-COF and AT-COF.


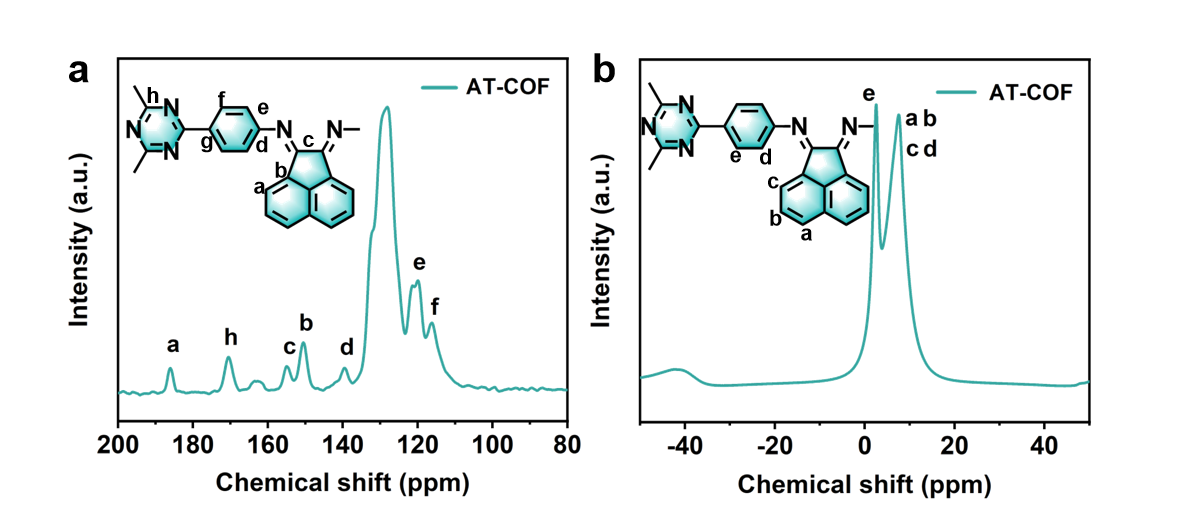


**Figure S21** (a) ^13^C NMR spectrum and (b) ^1^H NMR spectrum of AT-COF.


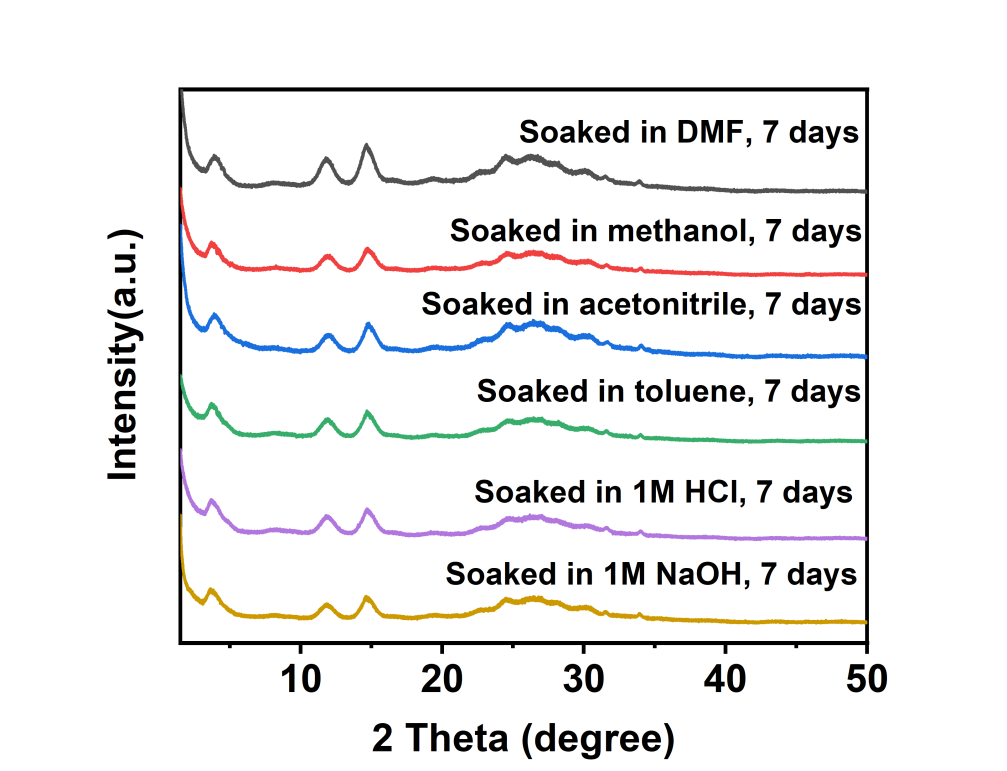


**Figure S22** The PXRD patterns of IrRu DAS/AT-COF after treated under harsh conditions.


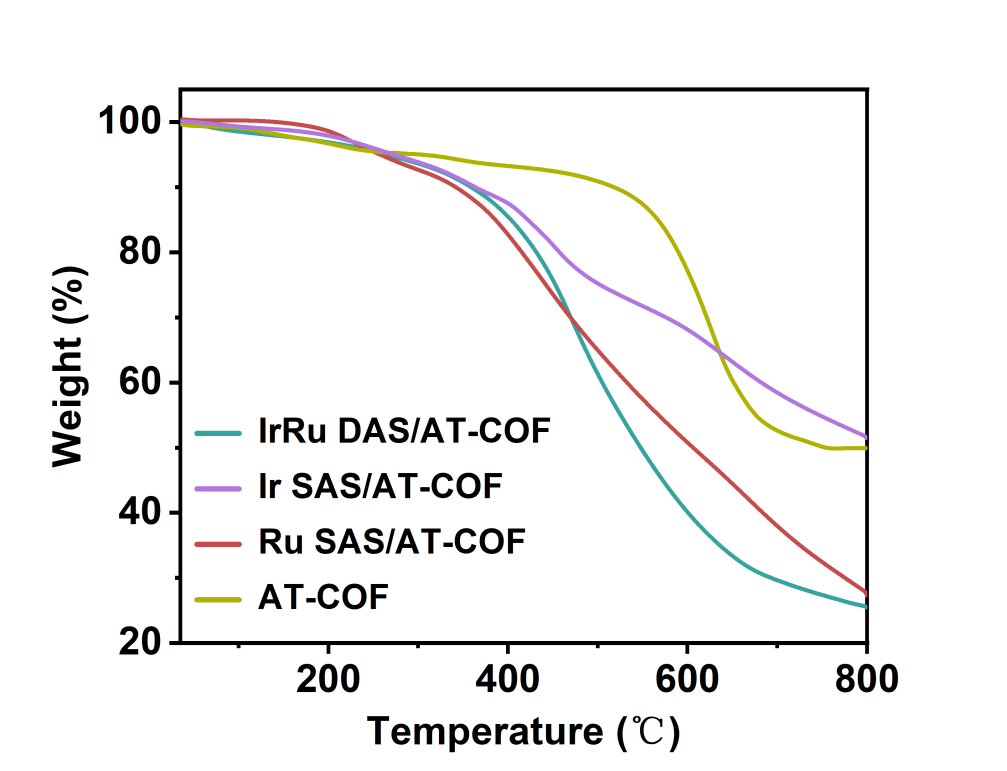


**Figure S23** The TGA curves of IrRu DAS/AT-COF, Ir SAS/AT-COF, Ru SAS/AT-COF and AT-COF.


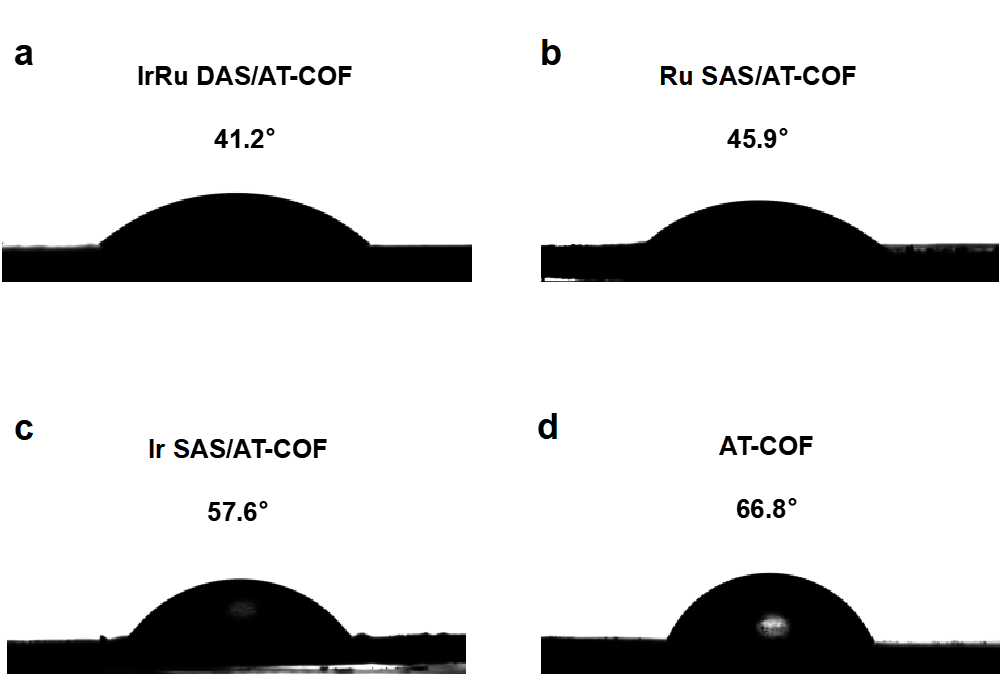


**Figure S24** The contact angle values of (a) IrRu DAS/AT-COF, (b) Ru SAS/AT-COF, (c) Ir SAS/AT-COF and (d) AT-COF.


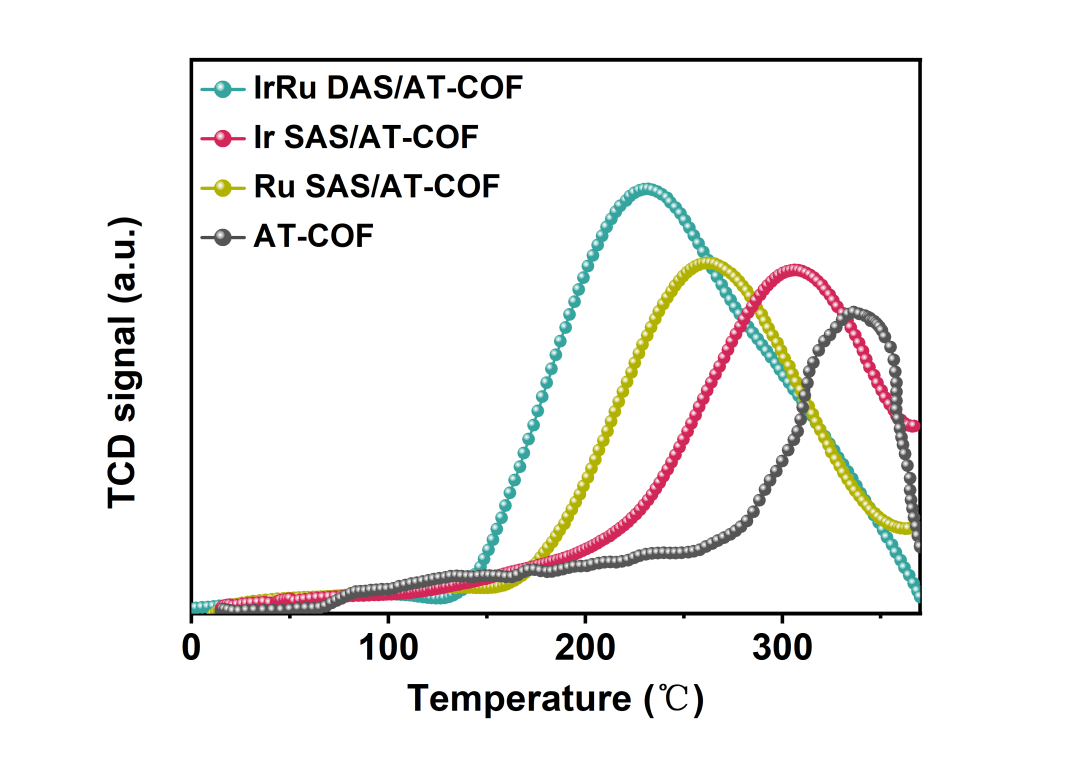


**Figure S25** The TPD-H_2_ curves of IrRu DAS/AT-COF, Ir SAS/AT-COF, Ru SAS/AT-COF and AT-COF.


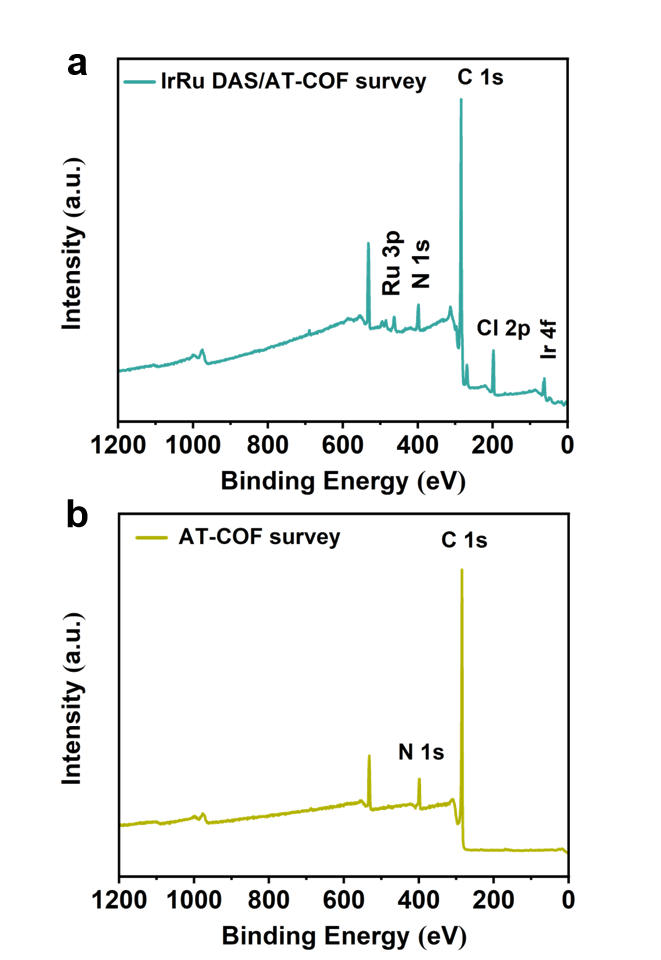


**Figure S26** The full range survey XPS spectra of (a) IrRu DAS/AT-COF and (b) AT-COF.


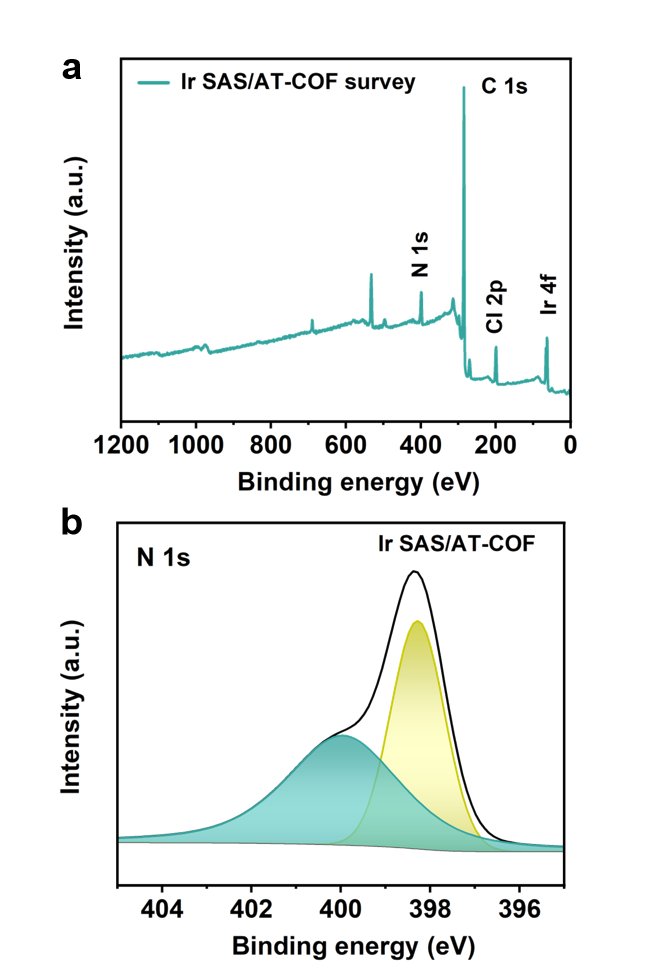


**Figure S27** (a) The full range survey and (b) high-resolution N 1s XPS spectra of Ir SAS/AT-COF.


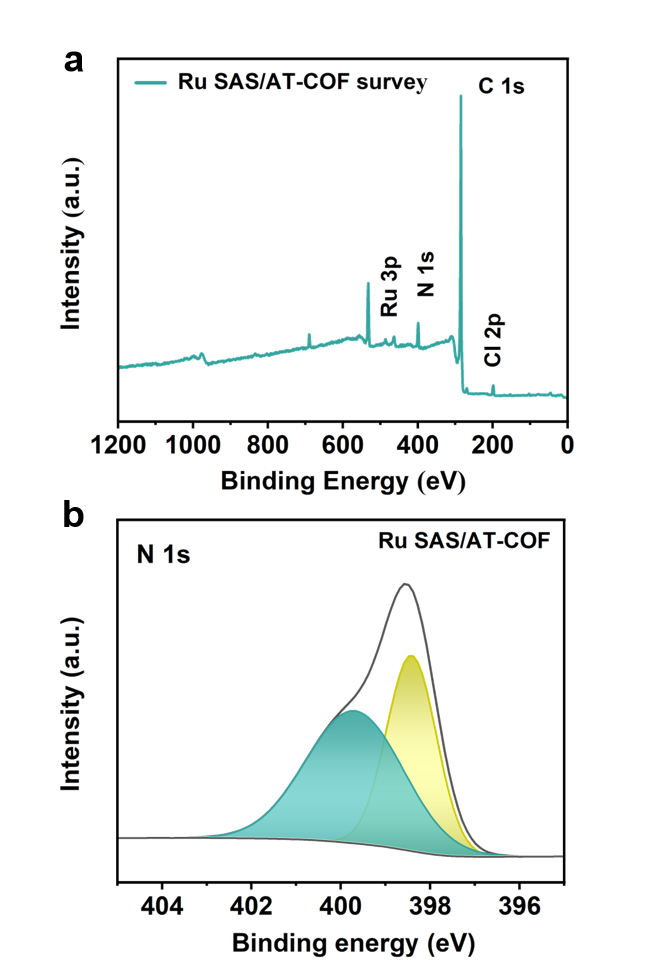


**Figure S28** (a) The full range survey and (b) high-resolution N 1s XPS spectra of Ru SAS/AT-COF.


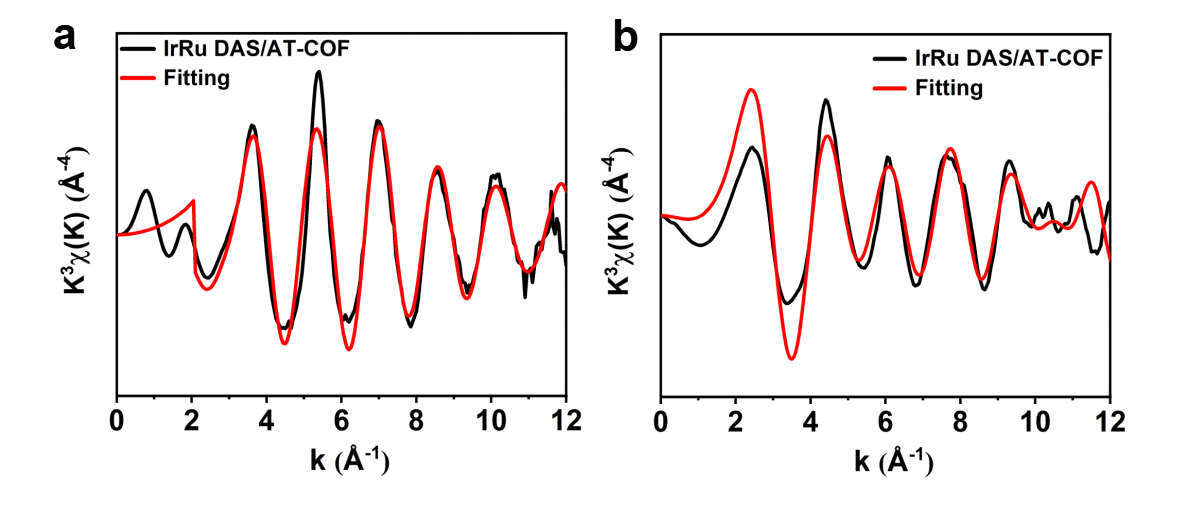
**Figure S29** The EXAFS fitting curves of IrRu DAS/AT-COF in k space.


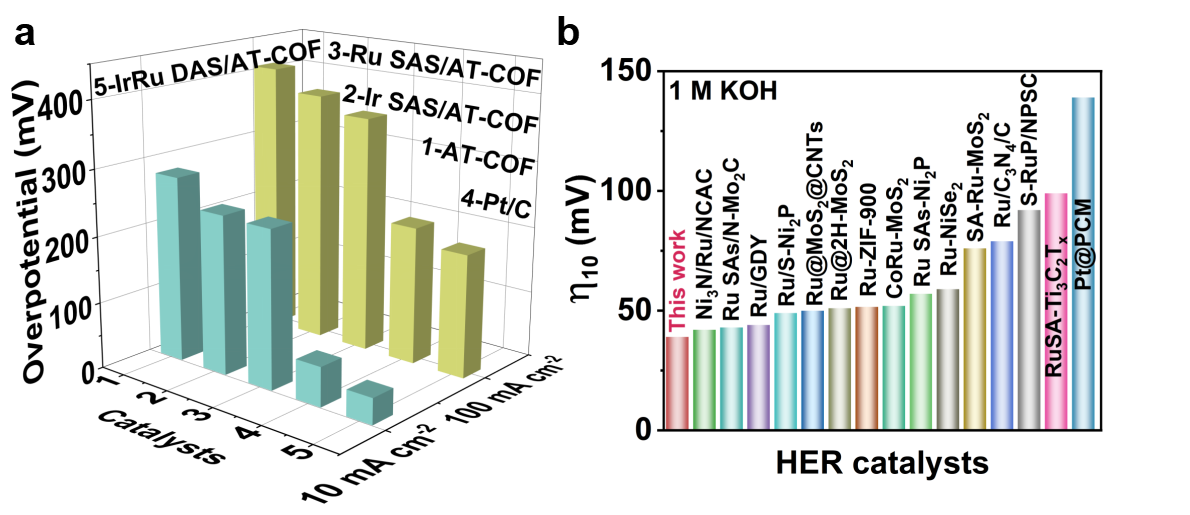


**Figure S30** (a) overpotentials curves and (b) the catalytic performance comparison of IrRu DAS/AT-COF with other noble-metal based HER catalysts.

**Note:** By extrapolating the Tafel plots (Figure S29), the much higher exchange current density (j_0_) of IrRu DAS/AT-COF is calculated to be 3.98 mA cm^−2^ versus that of AT-COF (0.24 mA cm^−2^), further verifying its remarkable HER activity.


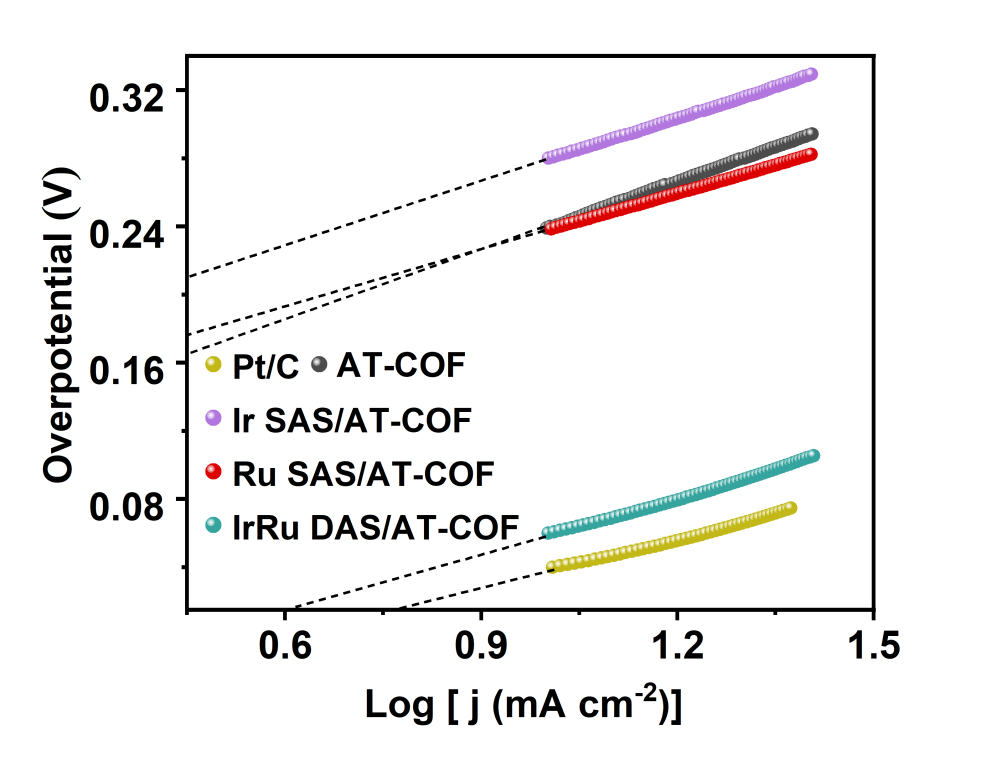


**Figure S31** The exchange current density plots of IrRu DAS/AT-COF, Ir SAS/AT-COF, Ru SAS/AT-COF, AT-COF, and commercial Pt/C in 1.0 M KOH.


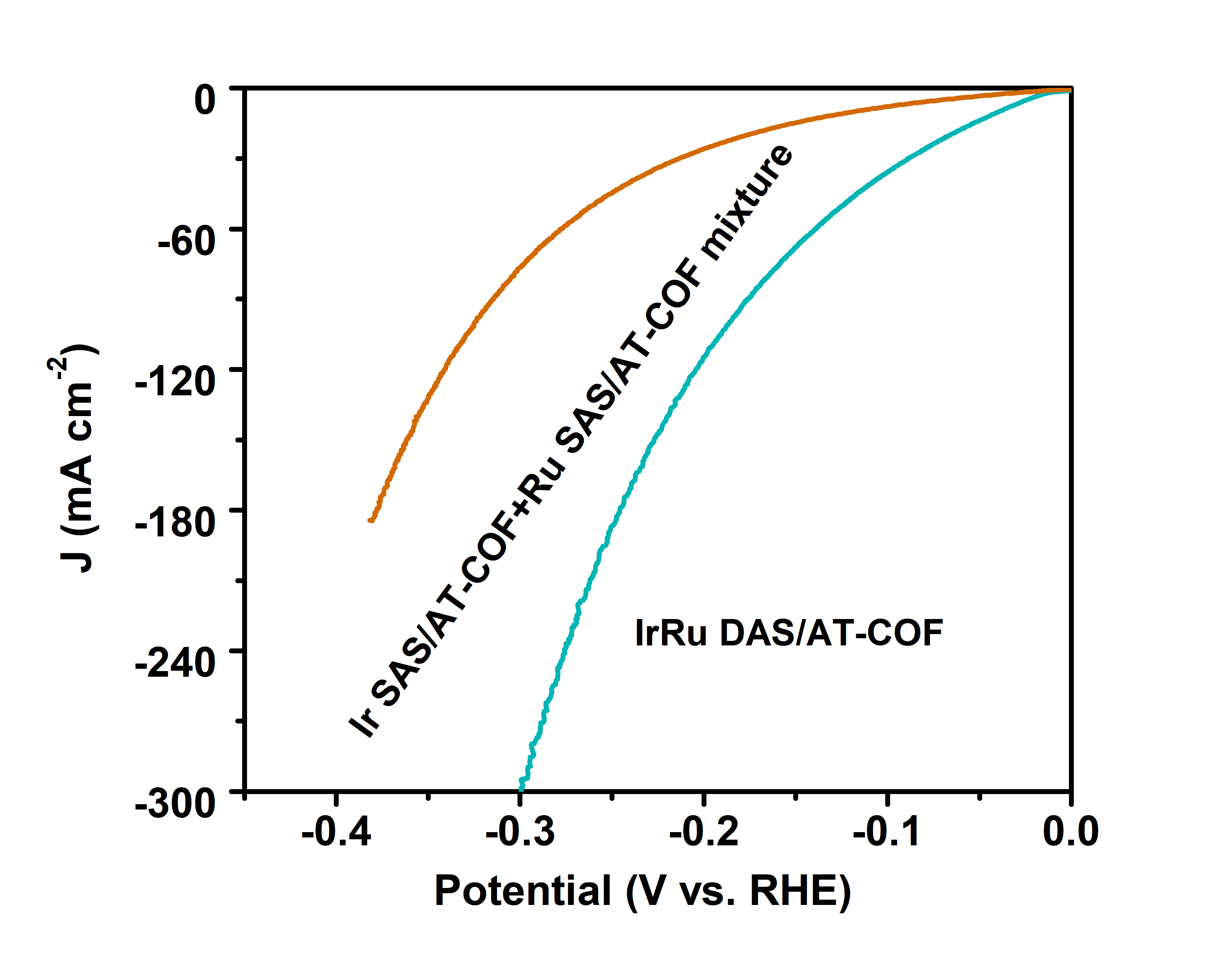


**Figure S32** The comparative LSV curves for HER IrRu DAS/AT-COF and Ir-SAS/AT-COF+Ru-SAS/AT-COF mixture.


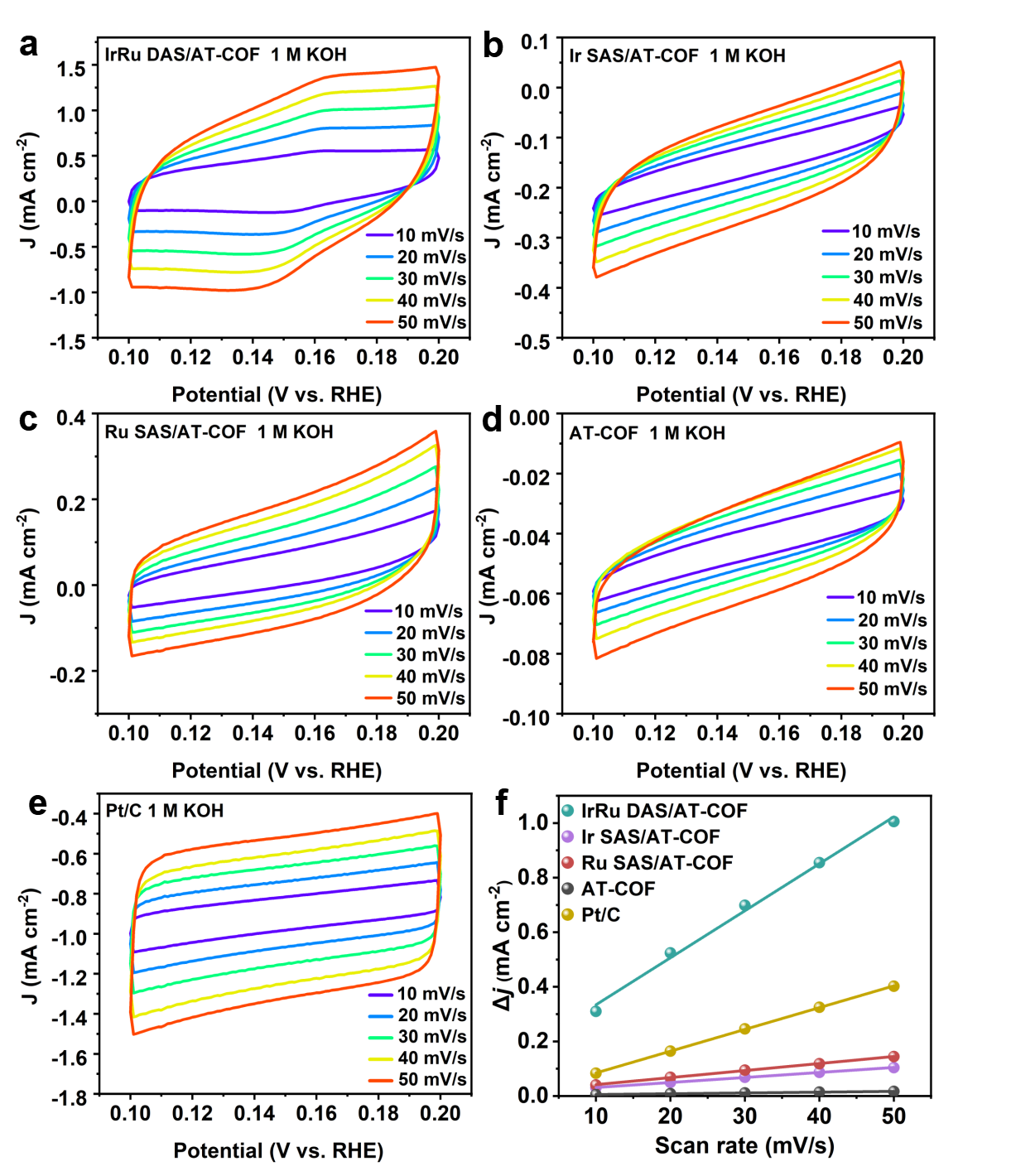


**Figure S33** (a-e) The CV curves at various scan rates and (f) C_dl_ plots of IrRu DAS/AT-COF, Ir SAS/AT-COF, Ru SAS/AT-COF, AT-COF, and commercial Pt/C in 1.0 M KOH.

**Note:** The geometric current density normalized by ECSA (j_ECSA_) to eliminate the effect of surface area can indicate the inherent HER activity (Figure S31). IrRu DAS/AT-COF shows a larger j_ECSA_ value than those of SAS catalysts, revealing its better intrinsic HER activity.


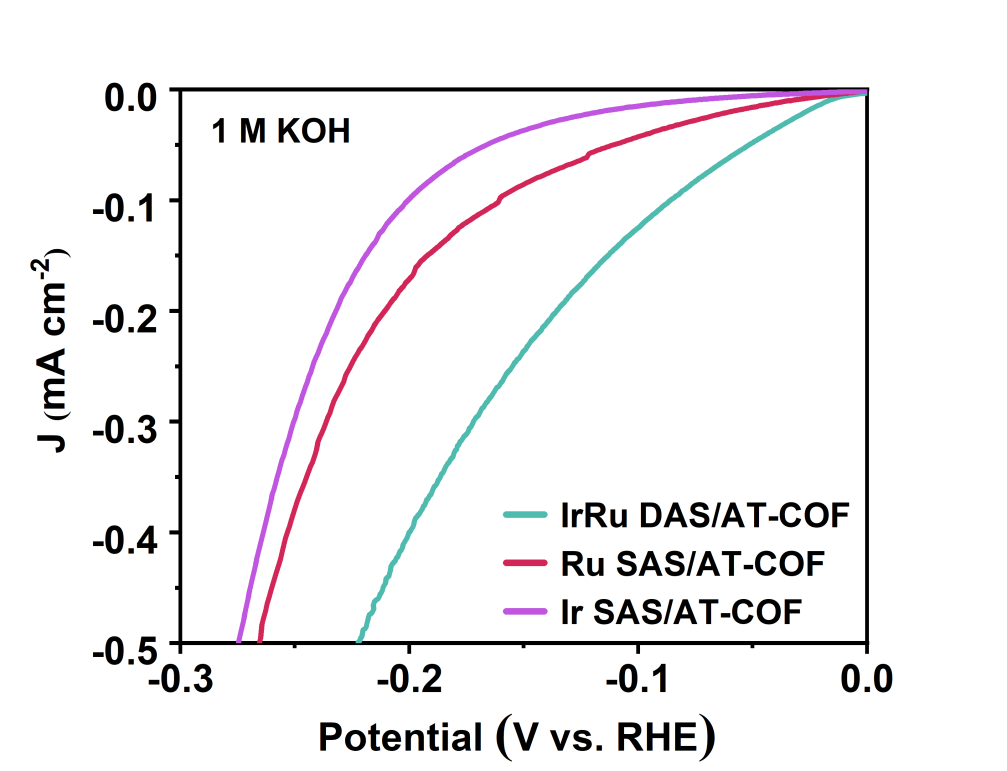


**Figure S34** The geometric current density was normalized by ECSA of IrRu DAS/AT-COF, Ir SAS/AT-COF and Ru SAS/AT-COF in 1.0 M KOH.


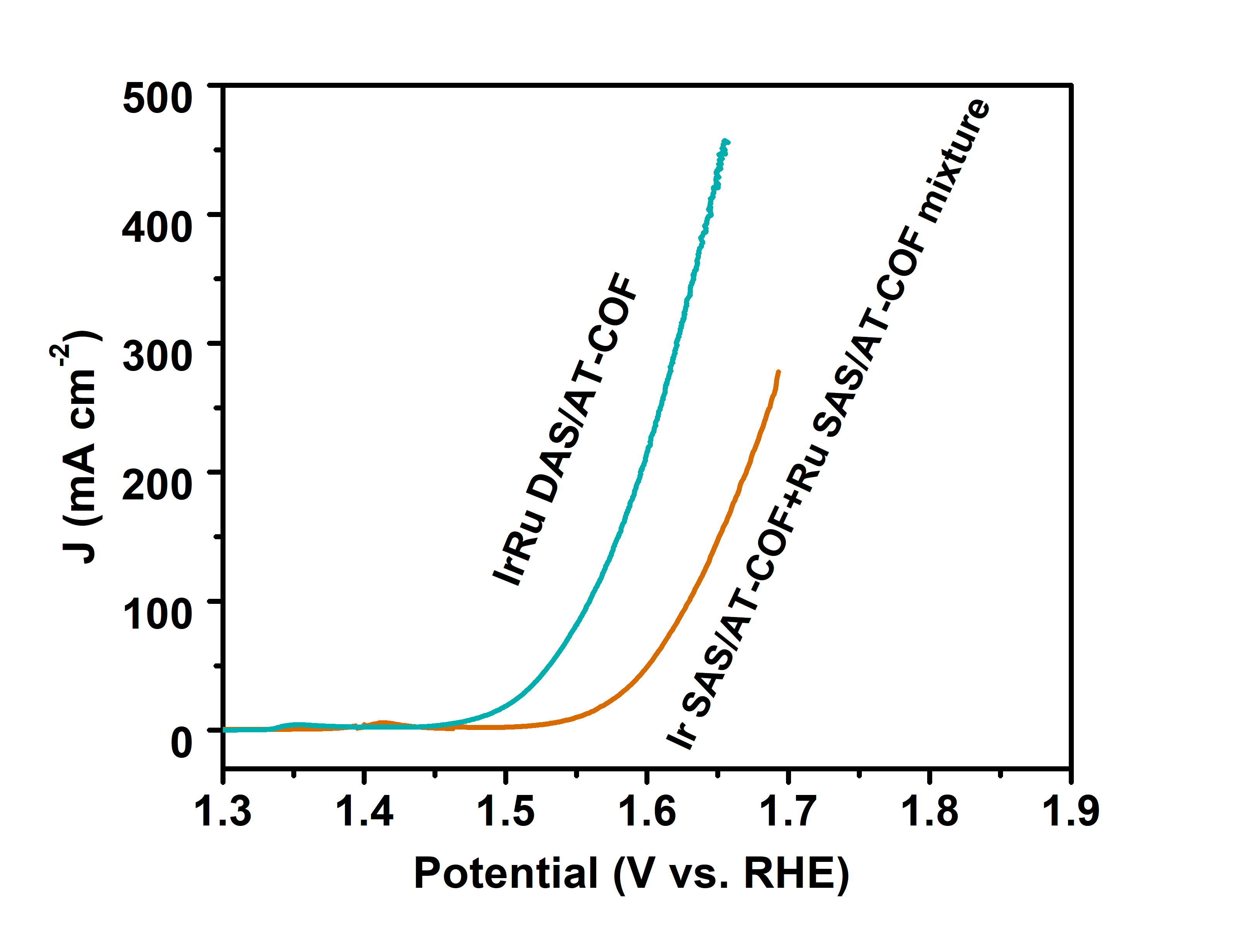


**Figure S35** The comparative LSV curves for OER of IrRu DAS/AT-COF and Ir-SAS/AT-COF+Ru-SAS/AT-COF mixture.


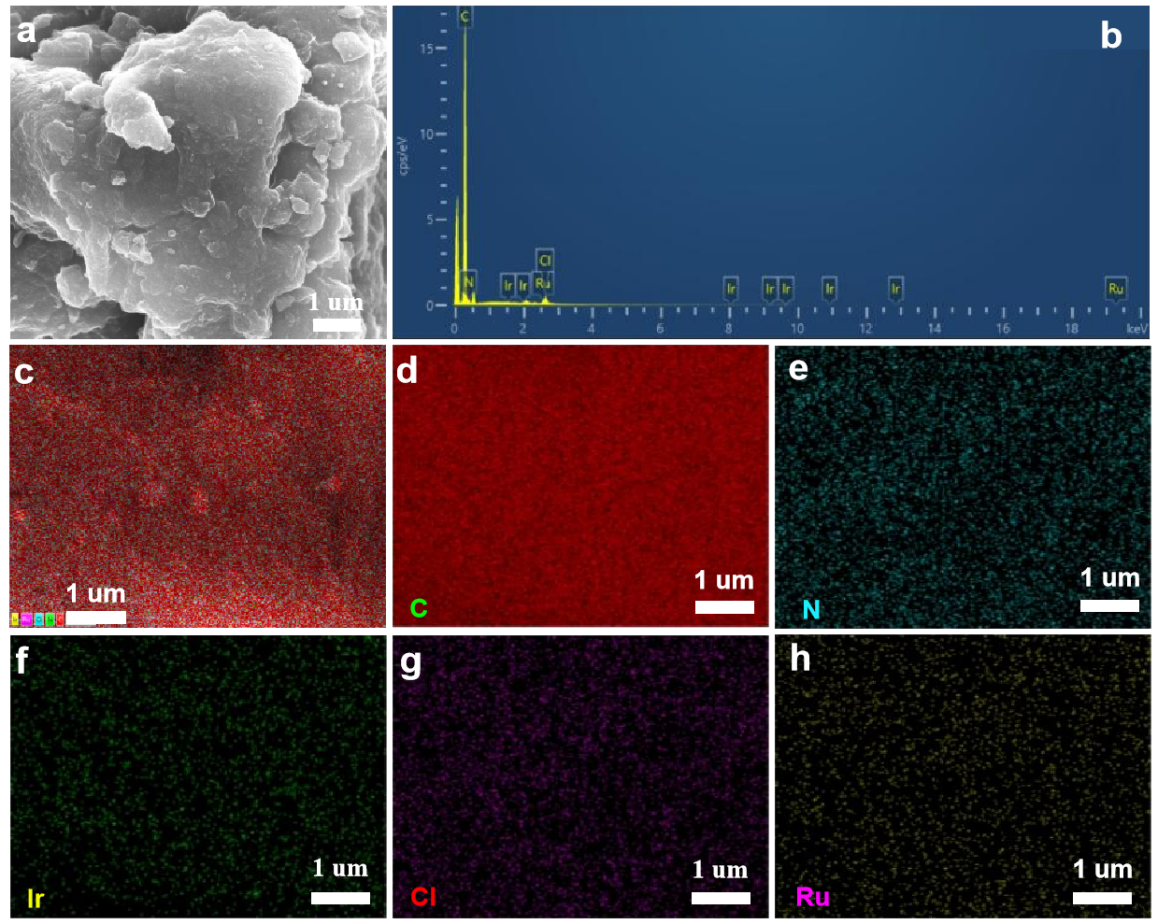


**Figure S36** (a) FESEM and (b-h) EDS mapping images of IrRu DAS/AT-COF after 100 h HER test.


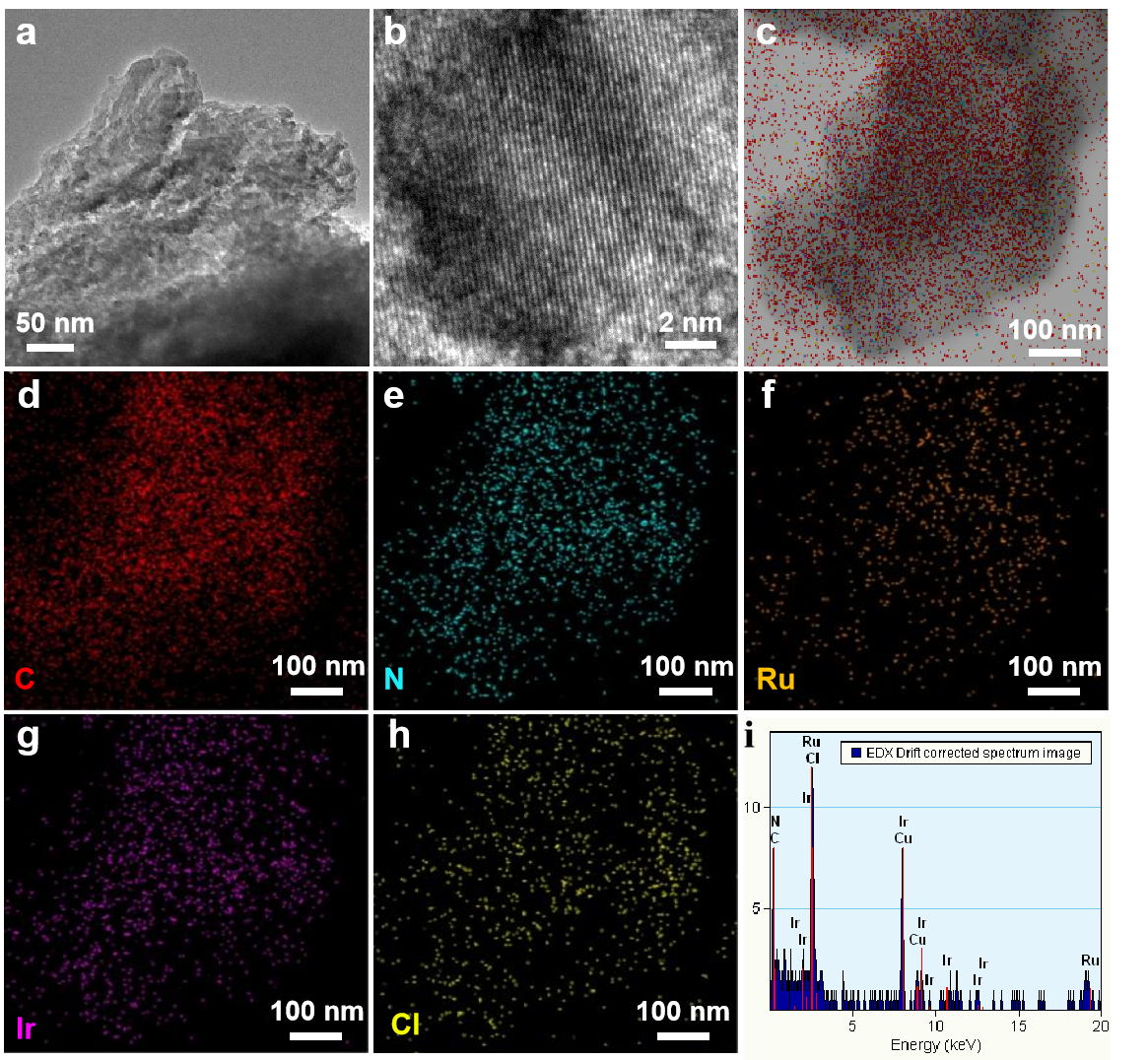


**Figure S37** (a) TEM, (b) HRTEM images, (c-h) element mappings and (i) EDS spectrum of IrRu DAS/AT-COF after 100 h HER stability test in 1M KOH.


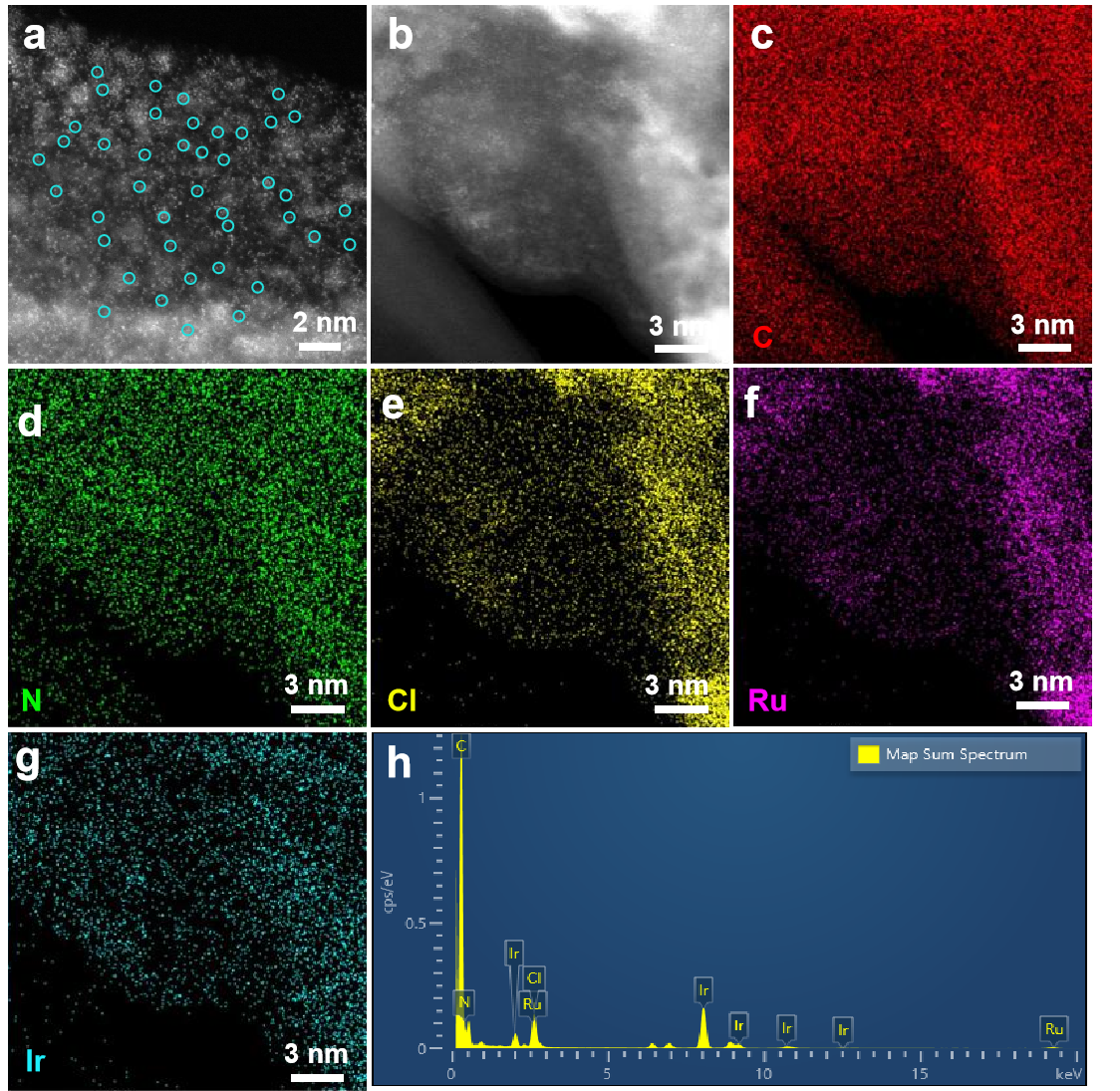


**Figure S38** (a) AC-HAADF-STEM image, (b-g) elements mapping and (h) EDS spectrum of IrRu DAS/AT-COF.


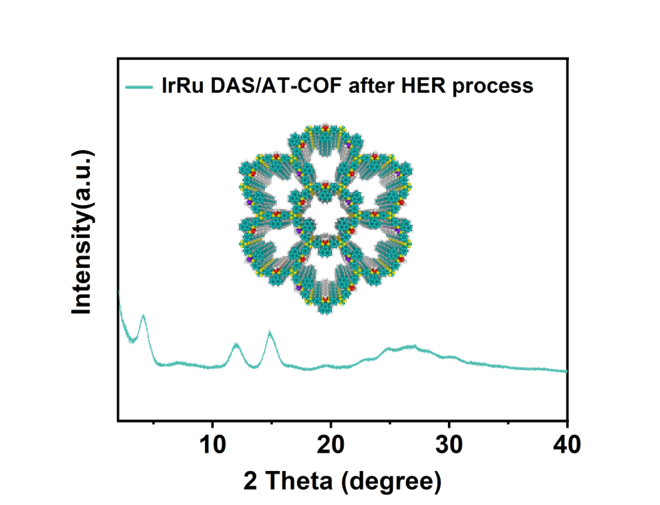


**Figure S39** The PXRD patterns of IrRu DAS/AT-COF after 100 h HER stability test.


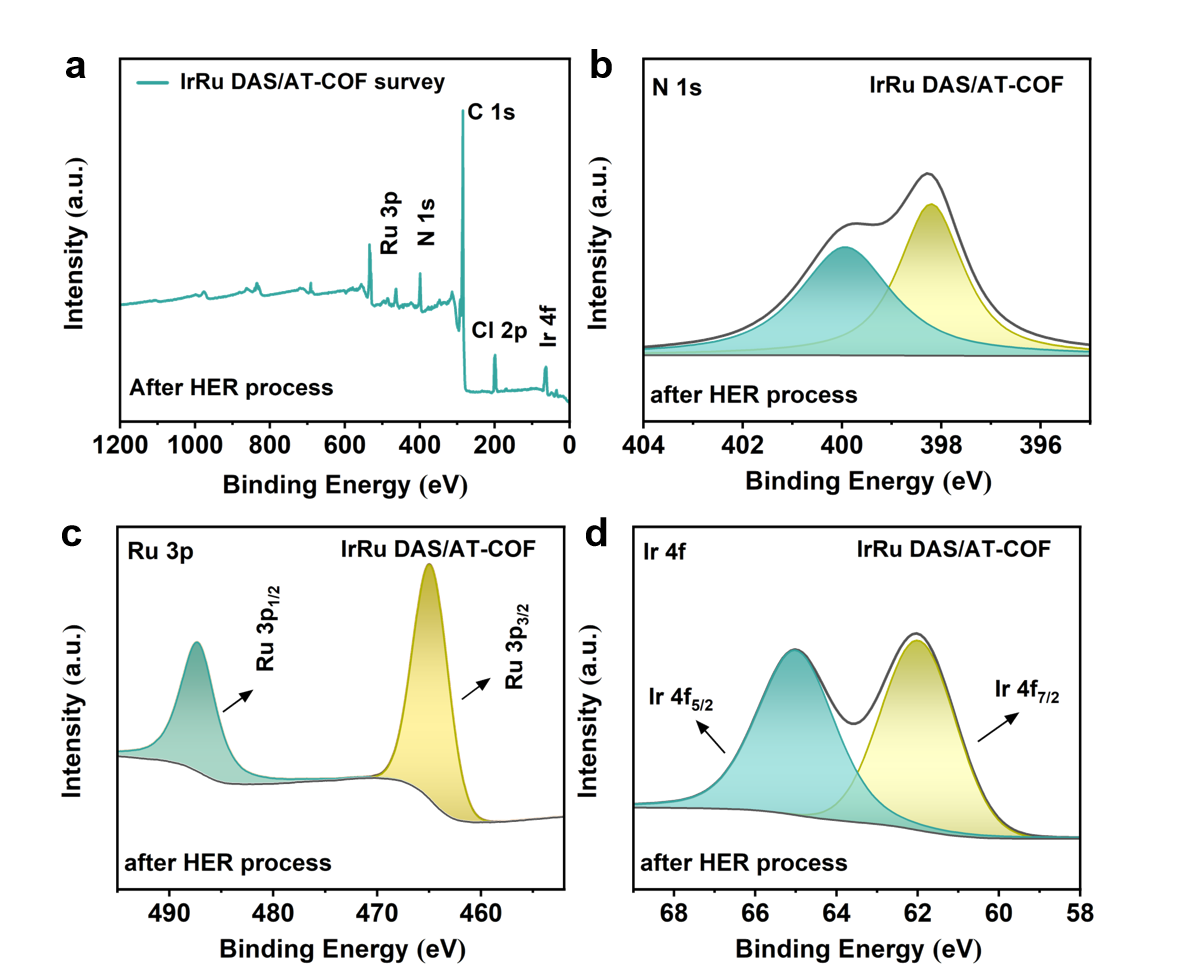


**Figure S40** (a) The full range survey, (b) high-resolution N 1s, (c) high-resolution Ru 3p and (d) high-resolution Ir 4f XPS spectra of IrRu DAS/AT-COF after 100 h HER stability test.


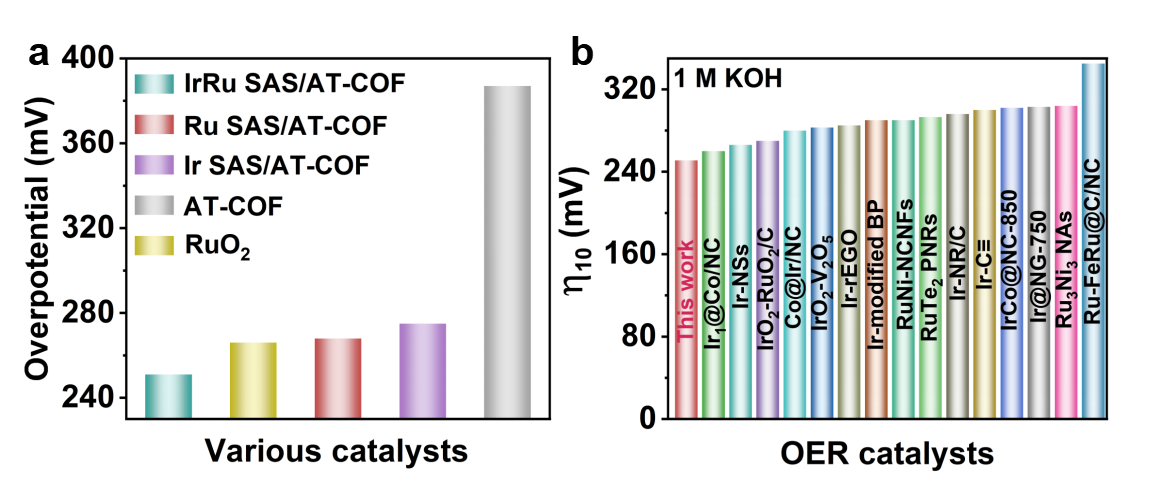


**Figure S41** (a) overpotentials curves and (b) the catalytic performance comparison of IrRu DAS/AT-COF with other noble-metal based OER catalysts.


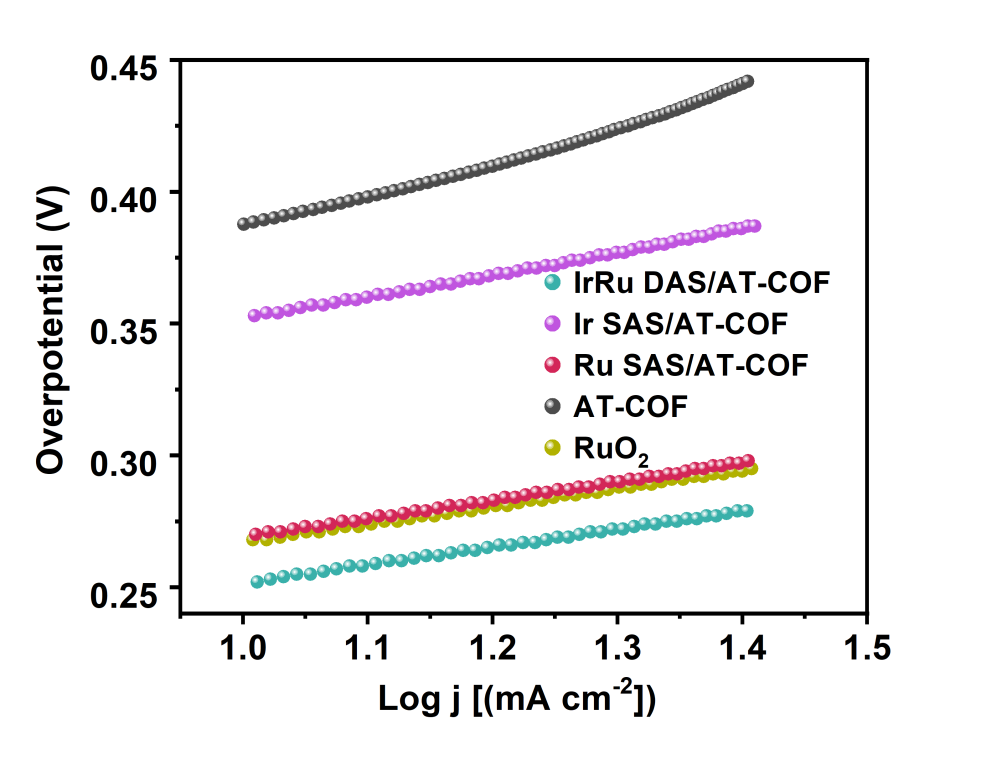


**Figure S42** The Tafel plots of IrRu DAS/AT-COF, Ir SAS/AT-COF, Ru SAS/AT-COF, AT-COF and RuO_2_ for OER in 1 M KOH.


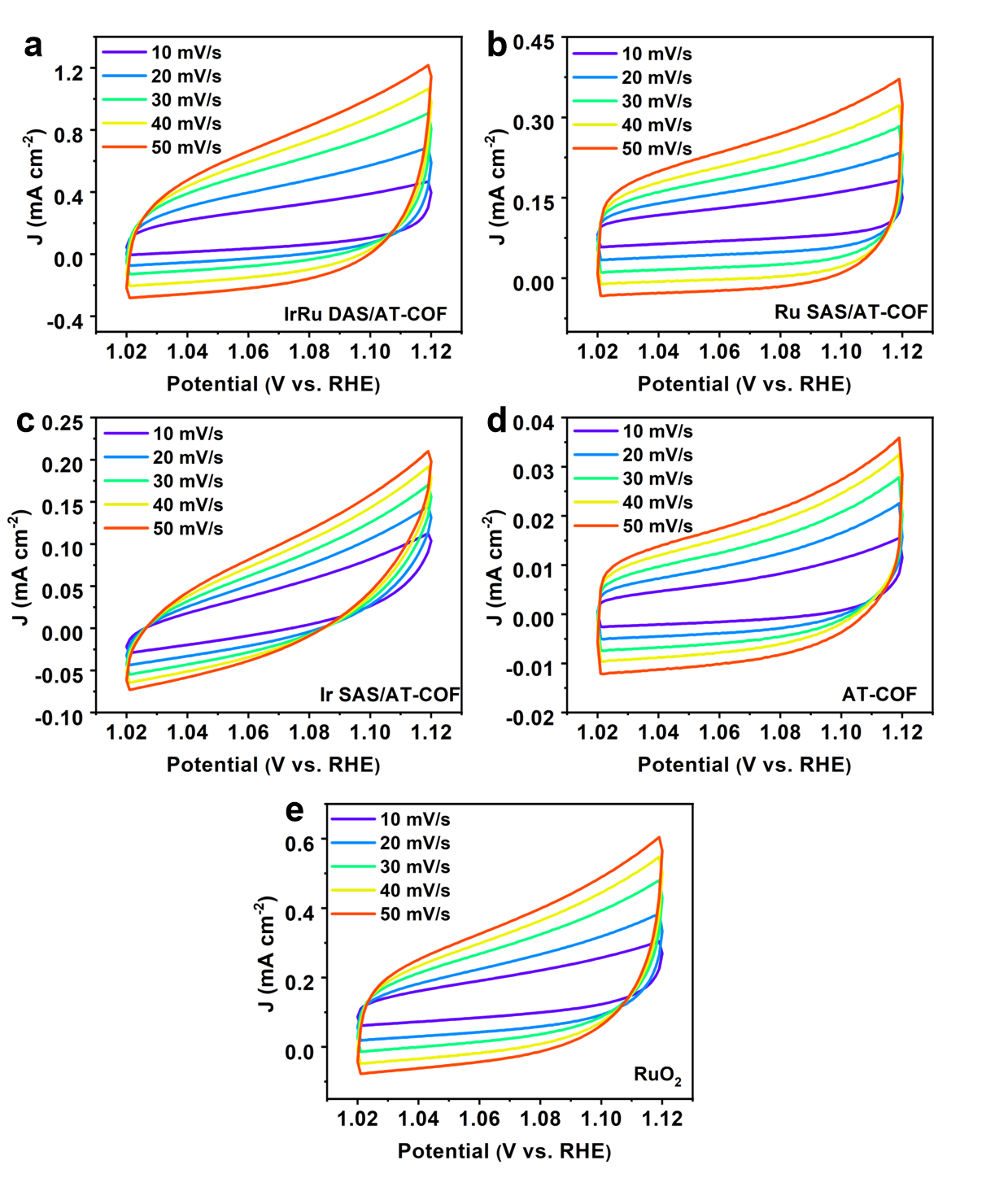


**Figure S43** The CV curves of (a) IrRu DAS/AT-COF, (b) Ru SAS/AT-COF, (c) Ir SAS/AT-COF, (d) AT-COF, and (e) commercial RuO_2_ in 1.0 M KOH.


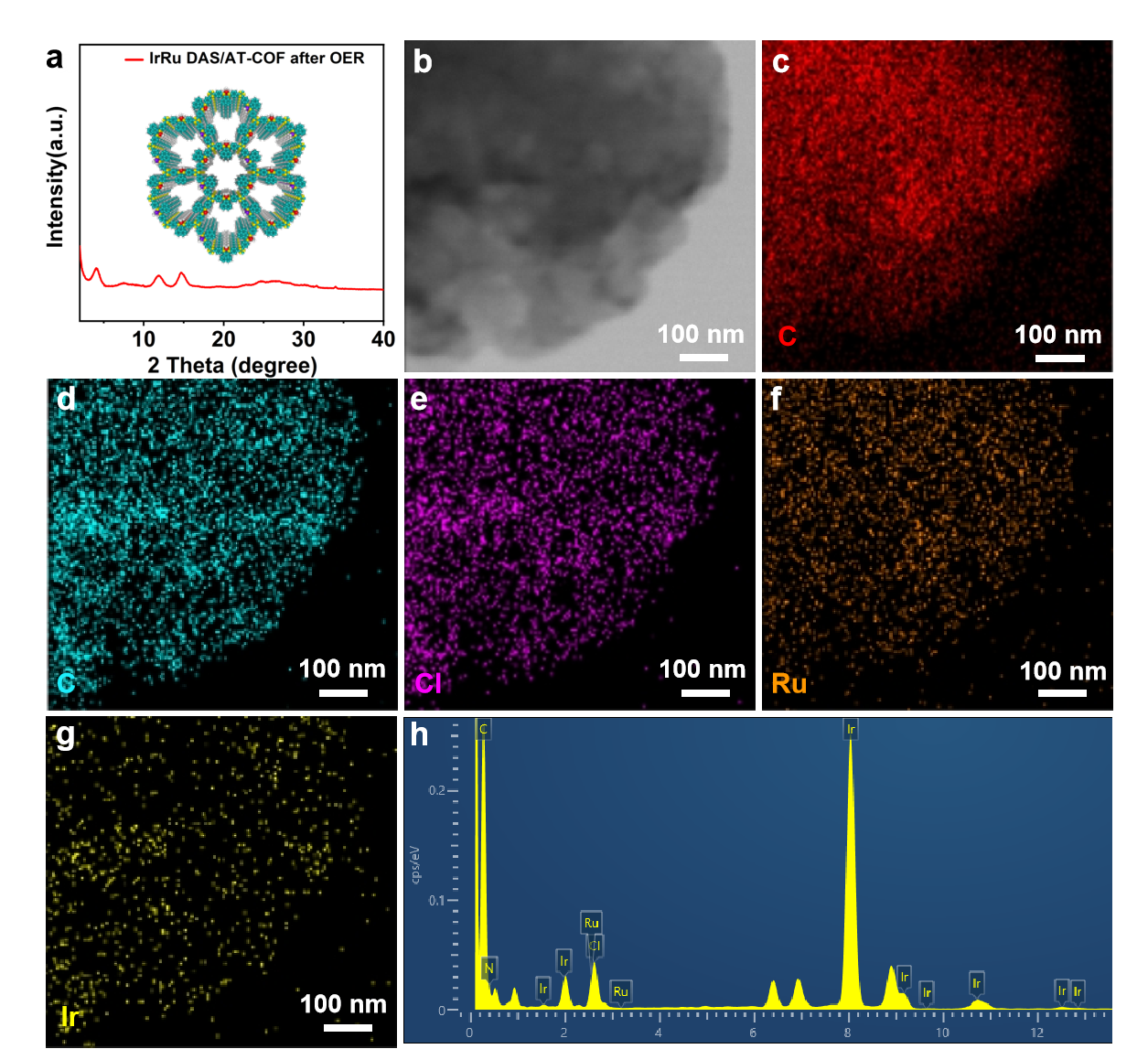


**Figure S44** (a) XRD pattern, (b) TEM image, (c-g) element mappings and (h) EDS spectrum of IrRu DAS/AT-COF after 100 h OER stability test in 1 M KOH.


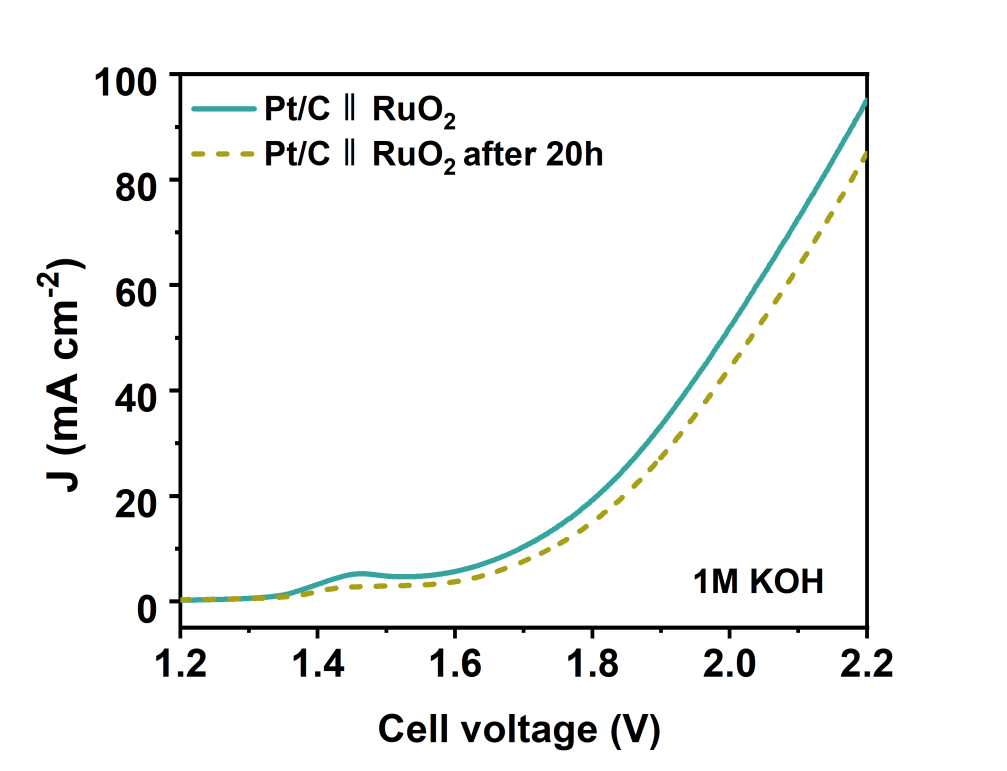


**Figure S45** The LSV curves of Pt/C||RuO_2_ system before and after 20 h overall water splitting test in 1M KOH.


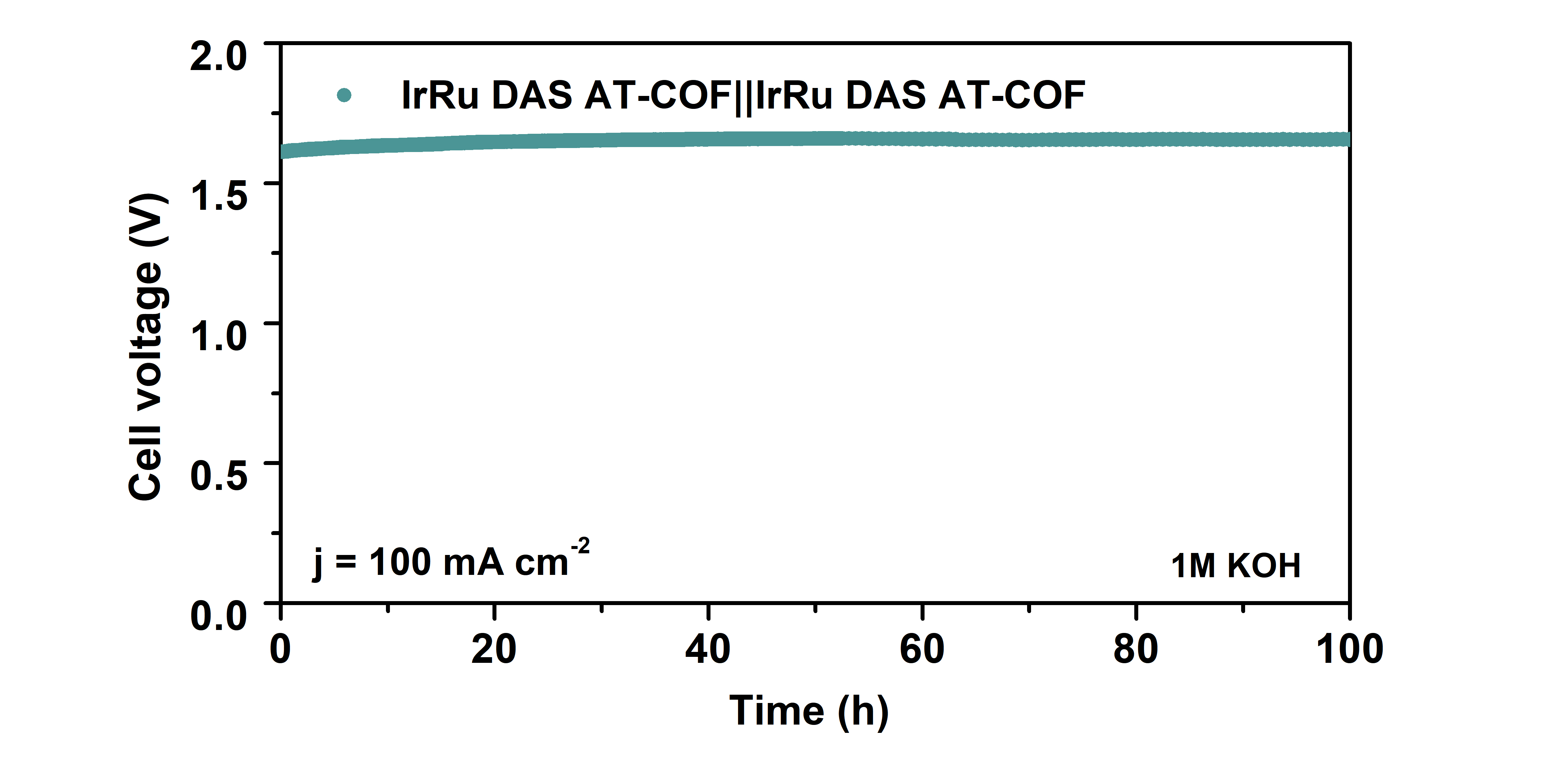


**Figure S46** The long-term plots of IrRu DAS/AT-COF‖IrRu DAS/AT-COF cell at 100 mA cm^-2^.


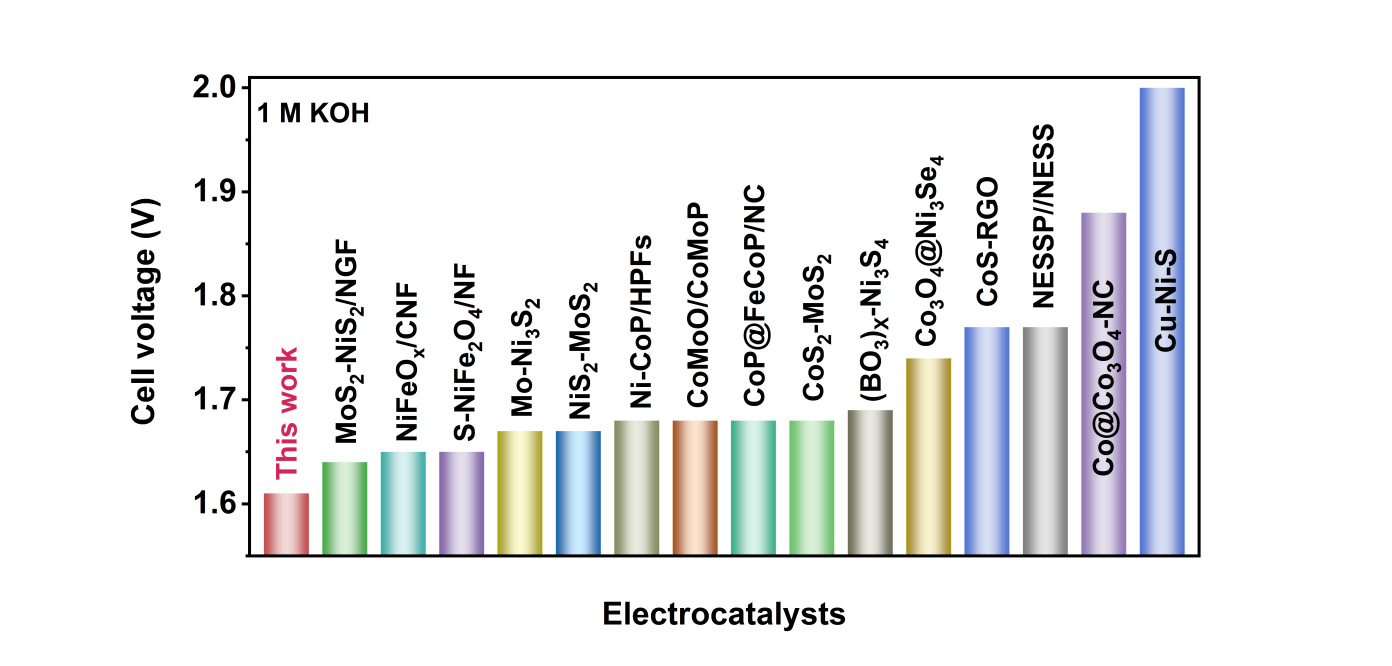


**Figure S47** The catalytic performance comparison of IrRu DAS/AT-COF with other catalysts for alkaline overall water splitting.


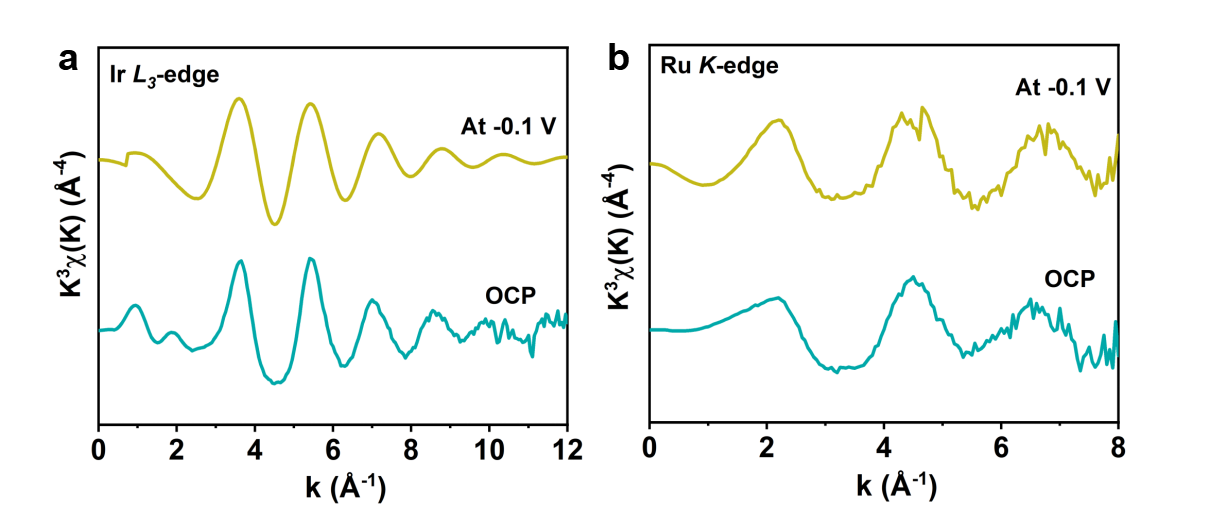


**Figure S48** The Re(k^3^χ(k)) oscillations of (a) Ir L_3_-edge operando EXAFS and (b) Ru *K*-edge operando EXAFS.


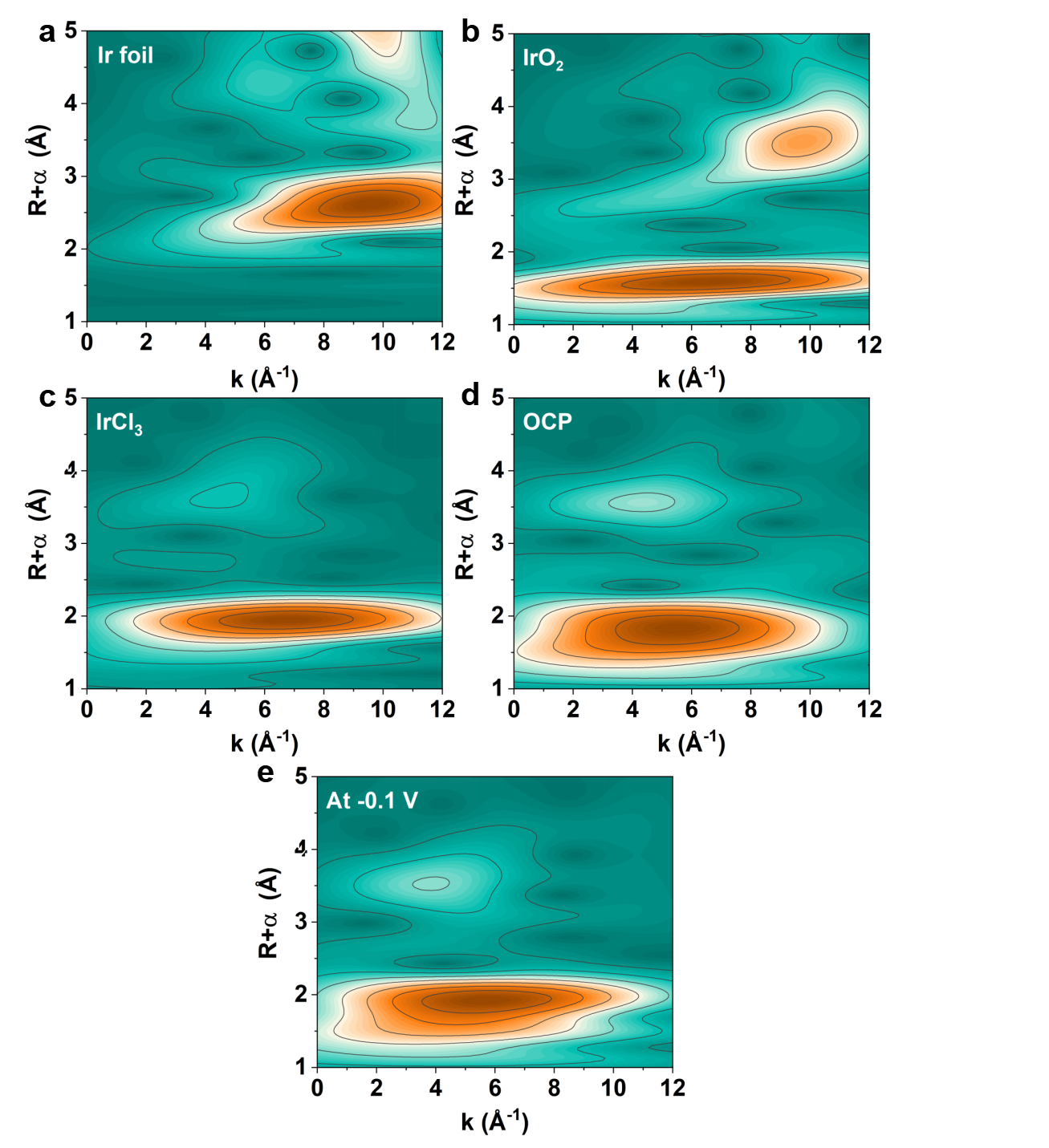


**Figure S49** The wavelet transform of k^3^-weighted in-situ EXAFS at Ir L_3_-edge of IrRu DAS/AT-COF at different applied potentials for alkaline HER.


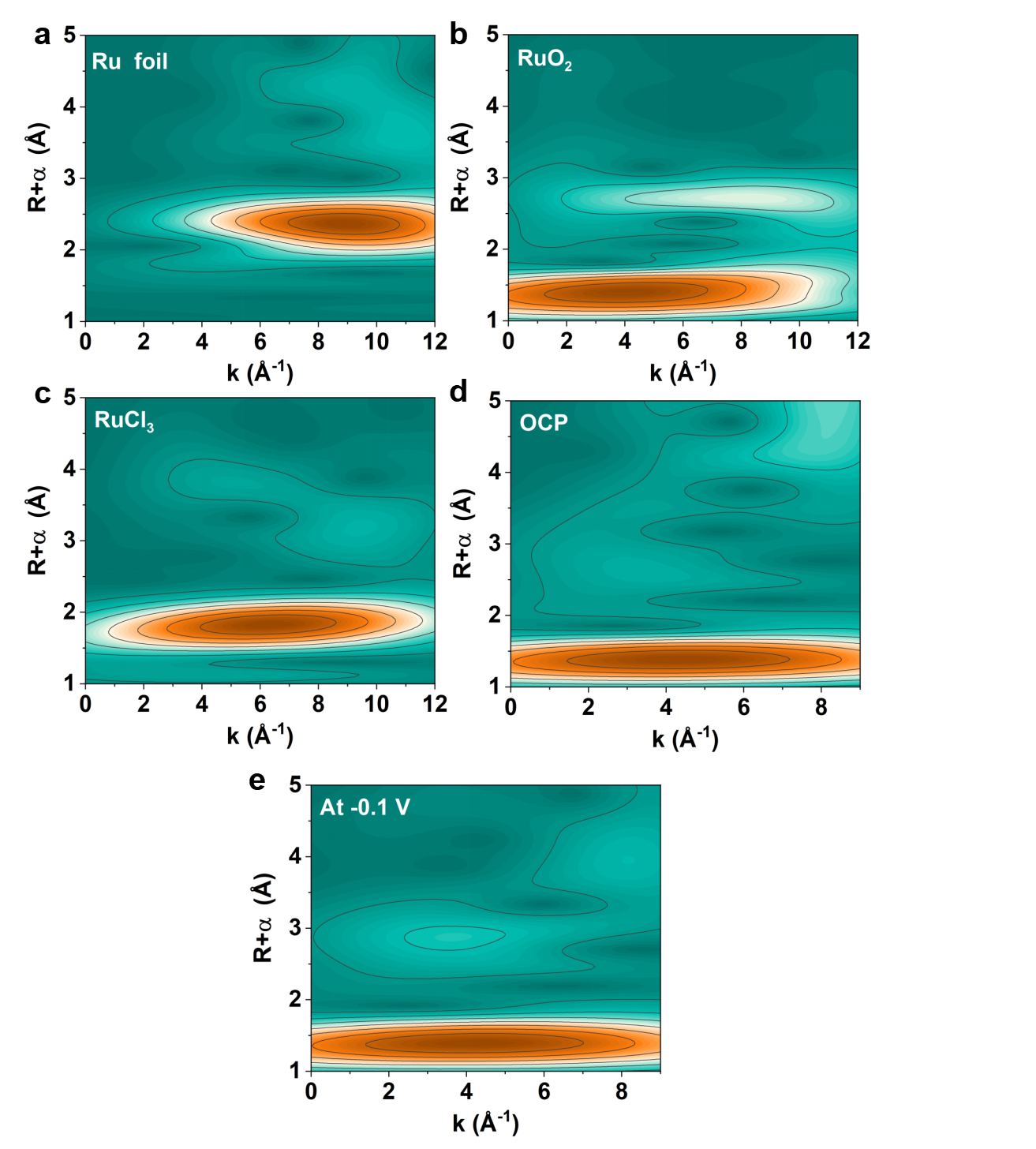


**Figure S50** The wavelet transform of k^3^-weighted in-situ EXAFS at Ru *K*-edge of IrRu DAS/AT-COF at different applied potentials for alkaline HER.


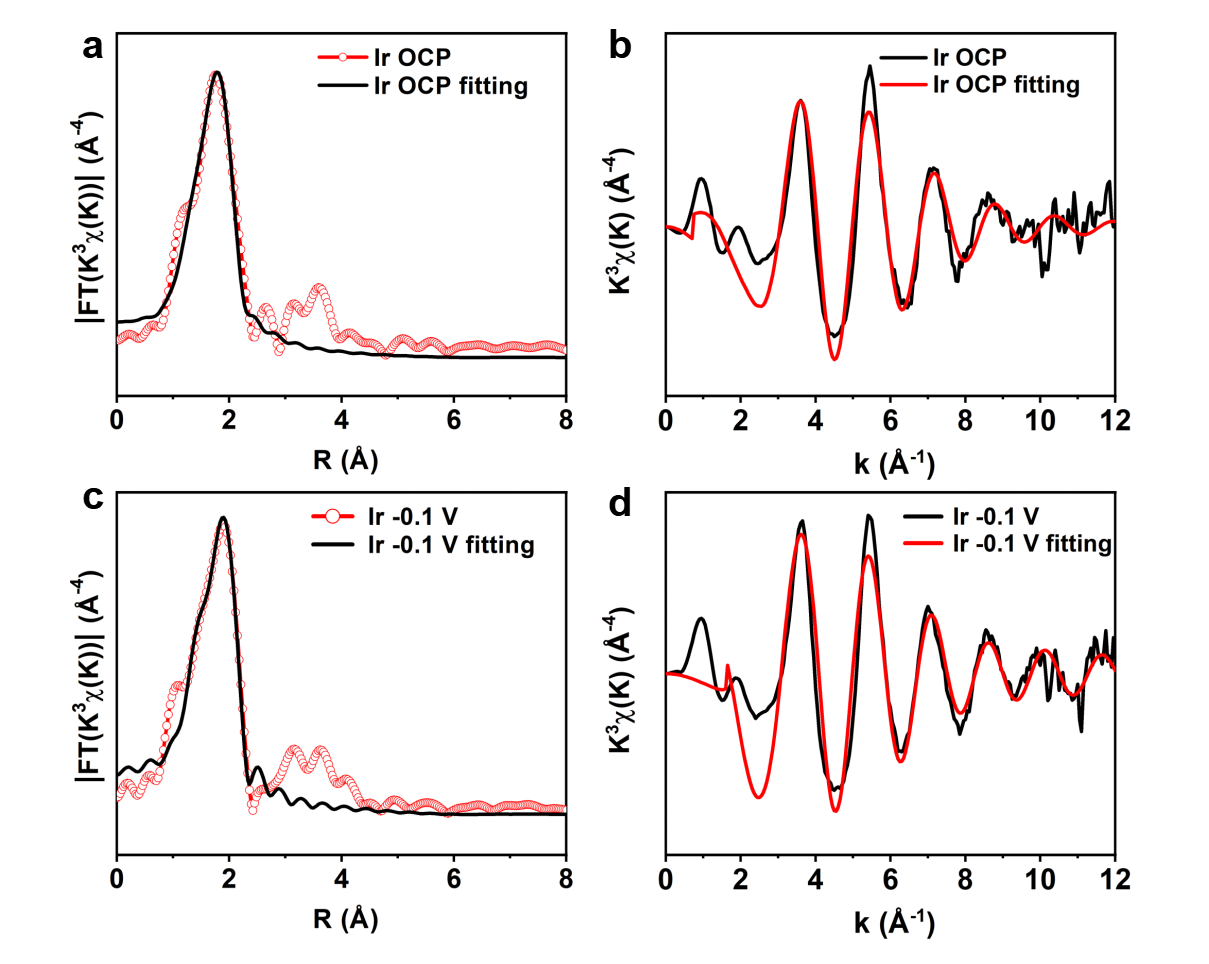


**Figure S51** The R-space curve-fitting of EXAFS spectra at the Ir L_3_-edge of the IrRu DAS/AT-COF (a) under open circuit condition and (c) at -0.1 V vs. RHE, respectively. The corresponding Re(k^3^χ(k)) oscillations of different fitting paths of the IrRu DAS/AT-COF (b) under open circuit condition and (d) at -0.1 V vs. RHE, respectively.


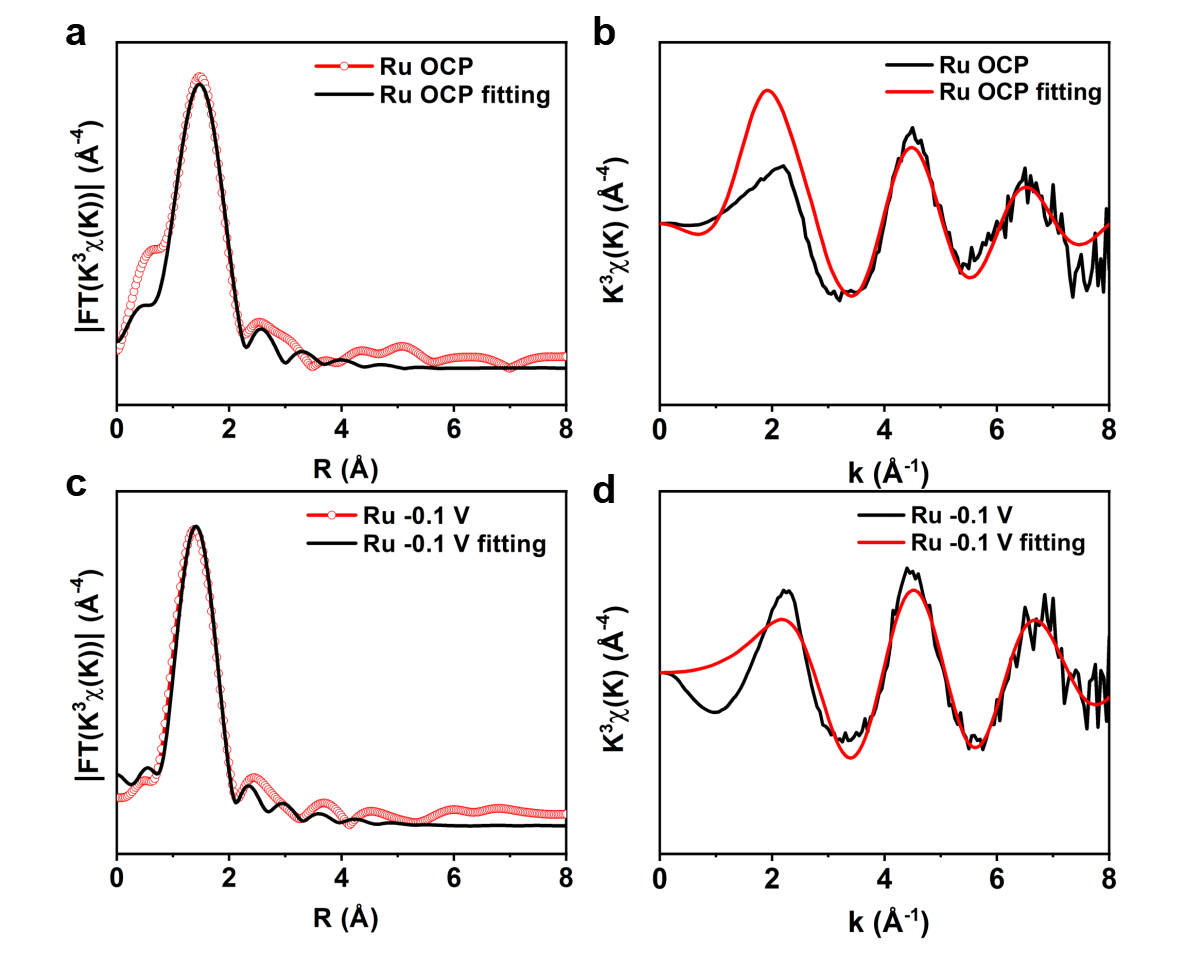


**Figure S52** The R-space curve-fitting of EXAFS spectra at the Ru K-edge of the IrRu DAS/AT-COF (a) under open circuit condition and (c) at -0.1 V vs. RHE, respectively. The corresponding Re(k^3^χ(k)) oscillations of different fitting paths of the IrRu DAS/AT-COF(b) under open circuit condition and (d) at -0.1 V vs. RHE, respectively.


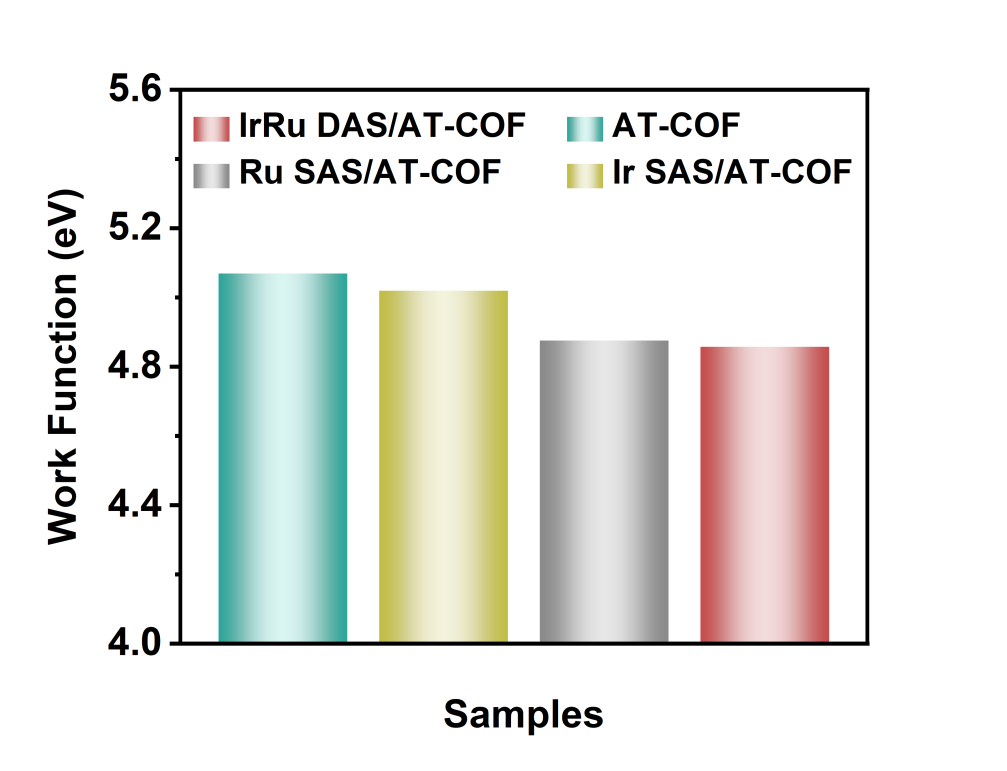


**Figure S53** The work function comparison of IrRu DAS/AT-COF, Ir SAS/AT-COF, Ru SAS/AT-COF and AT-COF.


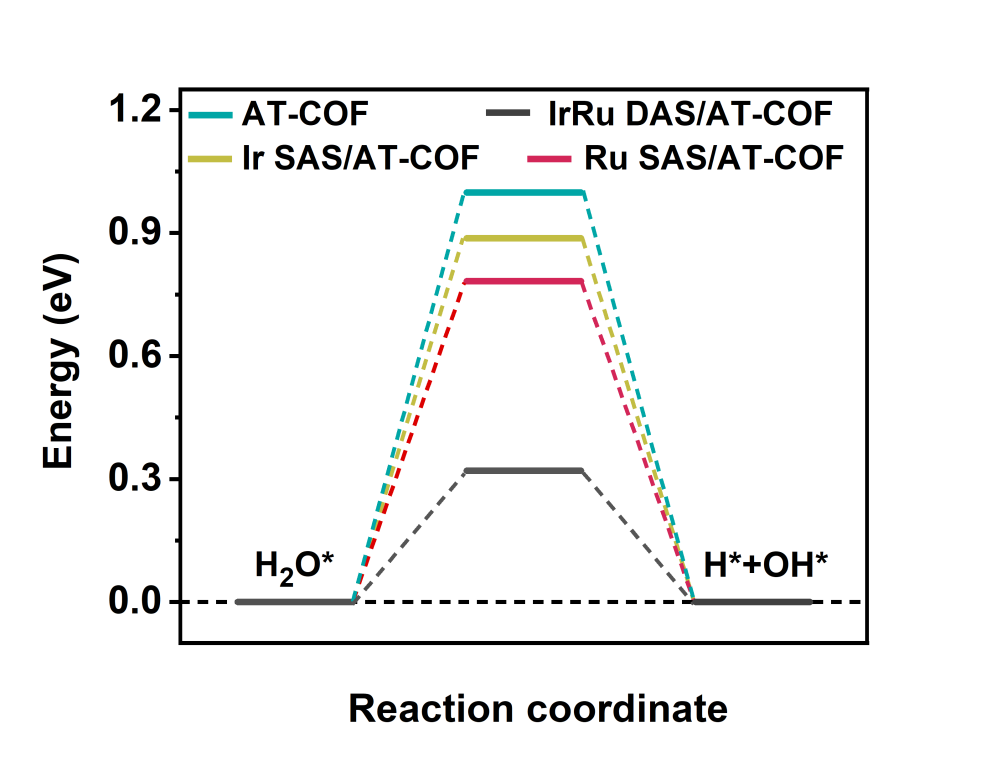


**Figure S54** The energy costs for water dissociation of IrRu DAS/AT-COF, Ir SAS/AT-COF, Ru SAS/AT-COF and AT-COF.


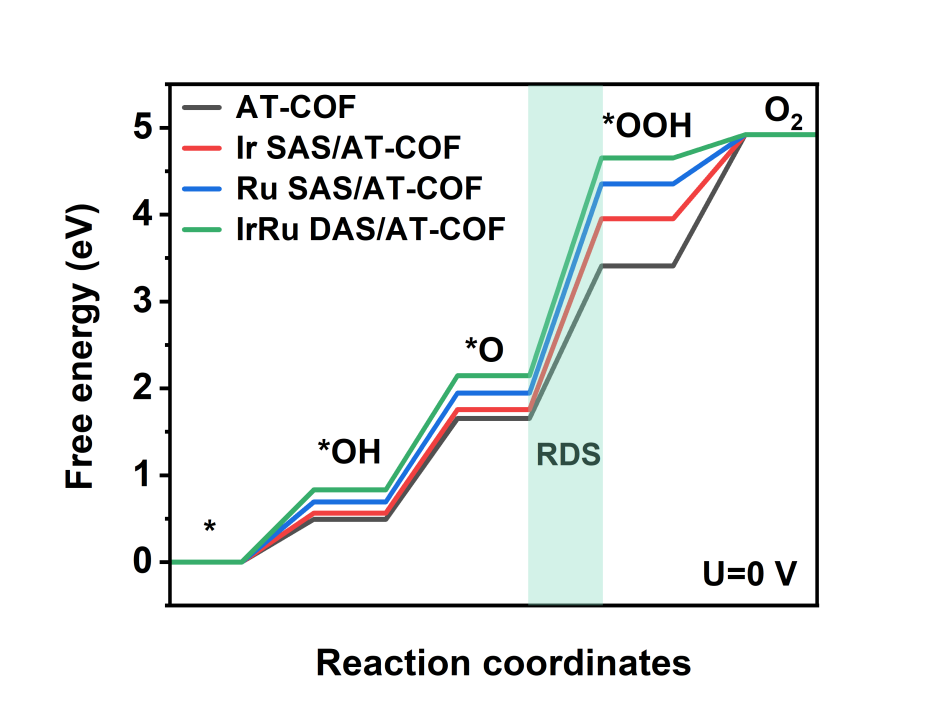


**Figure S55** Calculated free energy diagram for OER at potential U = 0 V versus RHE of IrRu DAS/AT-COF, Ir SAS/AT-COF, Ru SAS/AT-COF and AT-COF.

**Table S1** Fractional atomic coordinates for the unit cell of IrRu DAS/AT-COF catalyst.

| IrRu DAS/AT-COF Space group *P-6* (174) | | | |
| --- | --- | --- | --- |
| a = b = 28.7705 Å, c = 3.5503 Å; α = β=90°,γ=120° | | | |
| Atom | x (Å) | y (Å) | z (Å) |
| H1 | 0.67392 | 0.53846 | 0.5778 |
| H2 | 0.7537 | 0.61628 | 0.78798 |
| H3 | 0.75617 | 0.7017 | 0.88953 |
| H4 | 0.70383 | 0.75599 | 0.87837 |
| H5 | 0.61959 | 0.75497 | 0.76348 |
| H6 | 0.54145 | 0.67536 | 0.55393 |
| H7 | 0.55822 | 0.34482 | 0.69222 |
| H8 | 0.51725 | 0.403 | 0.73013 |
| H9 | 0.67129 | 0.45249 | 0.06614 |
| H10 | 0.62586 | 0.50718 | 0.07634 |
| H11 | 0.45548 | 0.67241 | 0.04846 |
| H12 | 0.50979 | 0.62668 | 0.06567 |
| H13 | 0.4017 | 0.51627 | 0.70118 |
| H14 | 0.344 | 0.55734 | 0.65445 |
| H15 | 0.46163 | 0.13596 | 0.53652 |
| H16 | 0.38457 | 0.13994 | 0.74191 |
| H17 | 0.29919 | 0.05805 | 0.85904 |
| H18 | 0.2449 | 0.95149 | 0.87799 |
| H19 | 0.24555 | 0.86733 | 0.7869 |
| H20 | 0.32422 | 0.86672 | 0.57912 |
| H21 | 0.65608 | 0.21235 | 0.64675 |
| H22 | 0.59862 | 0.11335 | 0.68771 |
| H23 | 0.54572 | 0.21657 | 0.03475 |
| H24 | 0.49161 | 0.11643 | 0.04814 |
| H25 | 0.32595 | 0.78079 | 0.06379 |
| H26 | 0.37094 | 0.88093 | 0.07483 |
| H27 | 0.48068 | 0.88556 | 0.72224 |
| H28 | 0.44012 | 0.78646 | 0.68335 |
| H29 | 0.86489 | 0.32624 | 0.56519 |
| H30 | 0.86313 | 0.24616 | 0.76938 |
| H31 | 0.94624 | 0.24379 | 0.86849 |
| H32 | 0.05261 | 0.29671 | 0.86516 |
| H33 | 0.1358 | 0.38161 | 0.75868 |
| H34 | 0.13421 | 0.45992 | 0.55387 |
| H35 | 0.78587 | 0.43944 | 0.69263 |
| H36 | 0.88515 | 0.48138 | 0.72838 |
| H37 | 0.78185 | 0.32863 | 0.05609 |
| H38 | 0.88211 | 0.37505 | 0.06539 |
| H39 | 0.21665 | 0.54463 | 0.04676 |
| H40 | 0.11643 | 0.49101 | 0.06091 |
| H41 | 0.11467 | 0.59758 | 0.70765 |
| H42 | 0.2138 | 0.65473 | 0.66514 |
| C43 | 0.28047 | 0.63634 | 0.34464 |
| C44 | 0.67304 | 0.5753 | 0.61714 |
| C45 | 0.71876 | 0.62016 | 0.7373 |
| C46 | 0.72039 | 0.66831 | 0.79388 |
| C47 | 0.67523 | 0.67422 | 0.73025 |
| C48 | 0.63024 | 0.62937 | 0.60812 |
| C49 | 0.62808 | 0.5795 | 0.55331 |
| C50 | 0.67026 | 0.72019 | 0.78401 |
| C51 | 0.62278 | 0.71941 | 0.71972 |
| C52 | 0.57776 | 0.67382 | 0.59981 |
| C53 | 0.58116 | 0.62817 | 0.54385 |
| C54 | 0.57301 | 0.54298 | 0.45634 |
| C55 | 0.56929 | 0.45864 | 0.40503 |
| C56 | 0.61244 | 0.47244 | 0.21684 |
| C57 | 0.61735 | 0.3951 | 0.37805 |
| C58 | 0.57339 | 0.38083 | 0.56014 |
| C59 | 0.5499 | 0.41288 | 0.57983 |
| C60 | 0.63732 | 0.44167 | 0.20904 |
| C61 | 0.71874 | 0.35547 | 0.36228 |
| C62 | 0.54392 | 0.57286 | 0.45133 |
| C63 | 0.45966 | 0.56929 | 0.3881 |
| C64 | 0.41256 | 0.54934 | 0.55283 |
| C65 | 0.39623 | 0.61771 | 0.3507 |
| C66 | 0.44371 | 0.63796 | 0.18926 |
| C67 | 0.47436 | 0.6129 | 0.20118 |
| C68 | 0.38073 | 0.5729 | 0.52819 |
| C69 | 0.36278 | 0.64346 | 0.34215 |
| C70 | 0.42478 | 0.09867 | 0.58314 |
| C71 | 0.38039 | 0.10069 | 0.70083 |
| C72 | 0.33229 | 0.05479 | 0.7662 |
| C73 | 0.32607 | 0.00319 | 0.7165 |
| C74 | 0.37041 | 0.00185 | 0.59611 |
| C75 | 0.42006 | 0.04896 | 0.52979 |
| C76 | 0.28029 | 0.95281 | 0.78374 |
| C77 | 0.28089 | 0.90561 | 0.73292 |
| C78 | 0.32599 | 0.90499 | 0.61418 |
| C79 | 0.37141 | 0.95342 | 0.54562 |
| C80 | 0.45624 | 0.02951 | 0.43904 |
| C81 | 0.54107 | 0.10898 | 0.37356 |
| C82 | 0.52679 | 0.13803 | 0.18537 |
| C83 | 0.60414 | 0.22074 | 0.34198 |
| C84 | 0.61964 | 0.19123 | 0.51825 |
| C85 | 0.58797 | 0.1358 | 0.53989 |
| C86 | 0.5573 | 0.19373 | 0.17548 |
| C87 | 0.64307 | 0.36169 | 0.36614 |
| C88 | 0.42611 | 0.97058 | 0.44742 |
| C89 | 0.42831 | 0.88907 | 0.39924 |
| C90 | 0.44793 | 0.8627 | 0.57261 |
| C91 | 0.3807 | 0.77759 | 0.37055 |
| C92 | 0.36017 | 0.80409 | 0.2049 |
| C93 | 0.38473 | 0.85969 | 0.21349 |
| C94 | 0.42469 | 0.80722 | 0.55273 |
| C95 | 0.35561 | 0.71858 | 0.35536 |
| C96 | 0.90267 | 0.32721 | 0.60383 |
| C97 | 0.90191 | 0.28131 | 0.72088 |
| C98 | 0.94848 | 0.27975 | 0.77617 |
| C99 | 0.99944 | 0.32526 | 0.71646 |
| C100 | 0.99952 | 0.37042 | 0.59724 |
| C101 | 0.95176 | 0.37236 | 0.54149 |
| C102 | 0.0504 | 0.33041 | 0.77252 |
| C103 | 0.09703 | 0.37826 | 0.71334 |
| C104 | 0.09634 | 0.42339 | 0.59589 |
| C105 | 0.04731 | 0.41987 | 0.53759 |
| C106 | 0.9701 | 0.42763 | 0.44807 |
| C107 | 0.88948 | 0.43065 | 0.39835 |
| C108 | 0.86048 | 0.38781 | 0.20773 |
| C109 | 0.77777 | 0.38141 | 0.373 |
| C110 | 0.80706 | 0.42508 | 0.55763 |
| C111 | 0.86259 | 0.44908 | 0.57598 |
| C112 | 0.80481 | 0.36236 | 0.20078 |
| C113 | 0.6369 | 0.27993 | 0.34223 |
| C114 | 0.02906 | 0.45703 | 0.44567 |
| C115 | 0.1096 | 0.54068 | 0.38737 |
| C116 | 0.13691 | 0.58705 | 0.55738 |
| C117 | 0.2214 | 0.60309 | 0.35504 |
| C118 | 0.19404 | 0.55623 | 0.18918 |
| C119 | 0.13832 | 0.52604 | 0.19889 |
| C120 | 0.19235 | 0.61846 | 0.53568 |
| N121 | 0.3091 | 0.61098 | 0.33636 |
| N122 | 0.54431 | 0.4906 | 0.41159 |
| N123 | 0.69704 | 0.38746 | 0.37485 |
| N124 | 0.49175 | 0.54472 | 0.40592 |
| N125 | 0.38817 | 0.69747 | 0.35055 |
| N126 | 0.50831 | 0.05245 | 0.39297 |
| N127 | 0.61096 | 0.30804 | 0.35003 |
| N128 | 0.45395 | 0.94599 | 0.40316 |
| N129 | 0.30166 | 0.68995 | 0.35279 |
| N130 | 0.94621 | 0.45601 | 0.40615 |
| N131 | 0.69059 | 0.30159 | 0.34421 |
| N132 | 0.05296 | 0.50924 | 0.40233 |
| Cl133 | 0.53159 | 0.01139 | 0.87572 |
| Cl134 | 0.00222 | 0.52558 | 0.886 |
| Cl135 | 0.93318 | 0.56155 | 0.33735 |
| Cl136 | 0.06302 | 0.62845 | 0.34733 |
| Cl137 | 0.43918 | 0.37233 | 0.32406 |
| Cl138 | 0.37204 | 0.43258 | 0.35739 |
| Cl139 | 0.62688 | 0.05783 | 0.34702 |
| Cl140 | 0.55803 | 0.93059 | 0.31311 |
| Cl141 | 0.46765 | 0.48009 | 0.88709 |
| Ru142 | 0.99909 | 0.53905 | 0.37534 |
| Ir143 | 0.46336 | 0.46156 | 0.37694 |
| Ir144 | 0.53521 | 0.99667 | 0.36614 |

**Table S2** Fractional atomic coordinates for the unit cell of AT-COF catalyst.

| AT-COF Space group *P-6* (174) | | | |
| --- | --- | --- | --- |
| a = b = 28.7705 Å, c = 3.5503 Å; α = β=90°,γ=120° | | | |
| Atom | x (Å) | y (Å) | z (Å) |
| H1 | 0.6683 | 0.52595 | 0.5 |
| H2 | 0.47405 | 0.14235 | 0.5 |
| H3 | 0.85765 | 0.3317 | 0.5 |
| H4 | 0.759 | 0.60921 | 0.5 |
| H5 | 0.39079 | 0.14979 | 0.5 |
| H6 | 0.85021 | 0.241 | 0.5 |
| H7 | 0.76928 | 0.70237 | 0.5 |
| H8 | 0.29763 | 0.06691 | 0.5 |
| H9 | 0.93309 | 0.23072 | 0.5 |
| H10 | 0.72175 | 0.75907 | 0.5 |
| H11 | 0.24093 | 0.96268 | 0.5 |
| H12 | 0.03732 | 0.27825 | 0.5 |
| H13 | 0.63522 | 0.75998 | 0.5 |
| H14 | 0.24002 | 0.87524 | 0.5 |
| H15 | 0.12476 | 0.36478 | 0.5 |
| H16 | 0.54353 | 0.67131 | 0.5 |
| H17 | 0.32869 | 0.87222 | 0.5 |
| H18 | 0.12778 | 0.45647 | 0.5 |
| H19 | 0.53409 | 0.31343 | 0.5 |
| H20 | 0.68657 | 0.22066 | 0.5 |
| H21 | 0.77934 | 0.46591 | 0.5 |
| H22 | 0.48305 | 0.36426 | 0.5 |
| H23 | 0.63574 | 0.11879 | 0.5 |
| H24 | 0.88121 | 0.51695 | 0.5 |
| H25 | 0.68617 | 0.46619 | 0.5 |
| H26 | 0.53381 | 0.21998 | 0.5 |
| H27 | 0.78002 | 0.31383 | 0.5 |
| H28 | 0.63525 | 0.51681 | 0.5 |
| H29 | 0.48319 | 0.11844 | 0.5 |
| H30 | 0.88156 | 0.36475 | 0.5 |
| H31 | 0.46591 | 0.68657 | 0.5 |
| H32 | 0.31343 | 0.77934 | 0.5 |
| H33 | 0.22066 | 0.53409 | 0.5 |
| H34 | 0.51695 | 0.63609 | 0.5 |
| H35 | 0.36391 | 0.88086 | 0.5 |
| H36 | 0.11914 | 0.48305 | 0.5 |
| H37 | 0.36475 | 0.48319 | 0.5 |
| H38 | 0.51681 | 0.88156 | 0.5 |
| H39 | 0.11844 | 0.63525 | 0.5 |
| H40 | 0.31383 | 0.53381 | 0.5 |
| H41 | 0.46619 | 0.78002 | 0.5 |
| H42 | 0.21998 | 0.68617 | 0.5 |
| C43 | 0.28158 | 0.64079 | 0.5 |
| C44 | 0.35921 | 0.64079 | 0.5 |
| C45 | 0.35921 | 0.71842 | 0.5 |
| C46 | 0.67133 | 0.56701 | 0.5 |
| C47 | 0.43299 | 0.10432 | 0.5 |
| C48 | 0.89568 | 0.32867 | 0.5 |
| C49 | 0.72189 | 0.61384 | 0.5 |
| C50 | 0.38616 | 0.10805 | 0.5 |
| C51 | 0.89195 | 0.27811 | 0.5 |
| C52 | 0.72759 | 0.66518 | 0.5 |
| C53 | 0.33482 | 0.06241 | 0.5 |
| C54 | 0.93759 | 0.27241 | 0.5 |
| C55 | 0.68193 | 0.6707 | 0.5 |
| C56 | 0.3293 | 0.01123 | 0.5 |
| C57 | 0.98877 | 0.31807 | 0.5 |
| C58 | 0.63274 | 0.6243 | 0.5 |
| C59 | 0.3757 | 0.00844 | 0.5 |
| C60 | 0.99156 | 0.36726 | 0.5 |
| C61 | 0.62593 | 0.57283 | 0.5 |
| C62 | 0.42717 | 0.0531 | 0.5 |
| C63 | 0.9469 | 0.37407 | 0.5 |
| C64 | 0.68211 | 0.71945 | 0.5 |
| C65 | 0.28055 | 0.96266 | 0.5 |
| C66 | 0.03734 | 0.31789 | 0.5 |
| C67 | 0.63339 | 0.71947 | 0.5 |
| C68 | 0.28053 | 0.91392 | 0.5 |
| C69 | 0.08608 | 0.36661 | 0.5 |
| C70 | 0.58333 | 0.67167 | 0.5 |
| C71 | 0.32833 | 0.91166 | 0.5 |
| C72 | 0.08834 | 0.41667 | 0.5 |
| C73 | 0.58377 | 0.62317 | 0.5 |
| C74 | 0.37683 | 0.9606 | 0.5 |
| C75 | 0.0394 | 0.41623 | 0.5 |
| C76 | 0.56915 | 0.53387 | 0.5 |
| C77 | 0.46613 | 0.03528 | 0.5 |
| C78 | 0.96472 | 0.43085 | 0.5 |
| C79 | 0.55641 | 0.44326 | 0.5 |
| C80 | 0.55674 | 0.11315 | 0.5 |
| C81 | 0.88685 | 0.44359 | 0.5 |
| C82 | 0.61226 | 0.47105 | 0.5 |
| C83 | 0.52895 | 0.14121 | 0.5 |
| C84 | 0.85879 | 0.38774 | 0.5 |
| C85 | 0.61267 | 0.38726 | 0.5 |
| C86 | 0.61274 | 0.22541 | 0.5 |
| C87 | 0.77459 | 0.38733 | 0.5 |
| C88 | 0.55684 | 0.35919 | 0.5 |
| C89 | 0.64081 | 0.19765 | 0.5 |
| C90 | 0.80235 | 0.44316 | 0.5 |
| C91 | 0.52881 | 0.38716 | 0.5 |
| C92 | 0.61284 | 0.14165 | 0.5 |
| C93 | 0.85835 | 0.47119 | 0.5 |
| C94 | 0.64041 | 0.44319 | 0.5 |
| C95 | 0.55681 | 0.19722 | 0.5 |
| C96 | 0.80278 | 0.35959 | 0.5 |
| C97 | 0.71842 | 0.35921 | 0.5 |
| C98 | 0.64079 | 0.35921 | 0.5 |
| C99 | 0.64079 | 0.28158 | 0.5 |
| C100 | 0.5407 | 0.56788 | 0.5 |
| C101 | 0.43212 | 0.97282 | 0.5 |
| C102 | 0.02718 | 0.4593 | 0.5 |
| C103 | 0.44359 | 0.55674 | 0.5 |
| C104 | 0.44326 | 0.88685 | 0.5 |
| C105 | 0.11315 | 0.55641 | 0.5 |
| C106 | 0.38774 | 0.52895 | 0.5 |
| C107 | 0.47105 | 0.85879 | 0.5 |
| C108 | 0.14121 | 0.61226 | 0.5 |
| C109 | 0.38733 | 0.61274 | 0.5 |
| C110 | 0.38726 | 0.77459 | 0.5 |
| C111 | 0.22541 | 0.61267 | 0.5 |
| C112 | 0.44316 | 0.64081 | 0.5 |
| C113 | 0.35919 | 0.80235 | 0.5 |
| C114 | 0.19765 | 0.55684 | 0.5 |
| C115 | 0.47119 | 0.61284 | 0.5 |
| C116 | 0.38716 | 0.85835 | 0.5 |
| C117 | 0.14165 | 0.52881 | 0.5 |
| C118 | 0.35959 | 0.55681 | 0.5 |
| C119 | 0.44319 | 0.80278 | 0.5 |
| C120 | 0.19722 | 0.64041 | 0.5 |
| N121 | 0.30581 | 0.61162 | 0.5 |
| N122 | 0.38838 | 0.69419 | 0.5 |
| N123 | 0.30581 | 0.69419 | 0.5 |
| N124 | 0.52838 | 0.47118 | 0.5 |
| N125 | 0.52882 | 0.0572 | 0.5 |
| N126 | 0.9428 | 0.47162 | 0.5 |
| N127 | 0.69419 | 0.38838 | 0.5 |
| N128 | 0.61162 | 0.30581 | 0.5 |
| N129 | 0.69419 | 0.30581 | 0.5 |
| N130 | 0.47162 | 0.52882 | 0.5 |
| N131 | 0.47118 | 0.9428 | 0.5 |
| N132 | 0.0572 | 0.52838 | 0.5 |

**Table S3** ICP-AES analysis results of different IrRu DAS/AT-COF catalysts.

| Catalysts | Metal amount | Metal amount |
| --- | --- | --- |
| IrRu DAS/AT-COF | Ru 0.56wt% | Ir 0.52wt% |

**Table S4** Textural parameters of IrRu DAS/AT-COF and AT-COF.

| Catalysts | BET surface area  (m^2^ g^−1^) | Pore diameter (nm) | Pore volume  (cm^3^ g^-1^) |
| --- | --- | --- | --- |
| IrRu DAS/AT-COF | 534.23 | 0.99 | 0.32 |
| AT-COF | 946.32 | 0.99 | 0.26 |

**Table S5** Structural parameters of the catalysts obtained from EXAFS fitting.

| Catalysts | Bond type | N | R  (Å) | ΔE_0_  (eV) | σ^2^×10^3^ (Å^2^) | R-factor |
| --- | --- | --- | --- | --- | --- | --- |
| Ir L_3_-edge | Ir-N | 2.0 | 2.12 | -6.8±0.4 | 7.7±7.2 | 0.02 |
|  | Ir-Cl | 3.0 | 2.38 |  | 9.2±0.8 |  |
| Ru K-edge | Ru-N | 2.0 | 2.09 | -4.5±7.6 | 9.7±1.1 | 0.02 |
|  | Ru-Cl | 3.0 | 2.35 |  | 9.4±8.9 |  |

N, coordination number; R, distance between absorber and backscatter atoms; ΔE_0_, inner potential correction to account for the difference in the inner potential between the catalyst and the reference compound. σ^2^, Debye-Waller factor.

**Table S6** HER performance comparison of the IrRu DAS/AT-COF with other state-of-the-art noble-metal-based electrocatalysts reported previously in 1 M KOH.

| Catalysts | η_10_  (mV) | Noble metal loading | Electrolyte | Ref. |
| --- | --- | --- | --- | --- |
| IrRu DAS/AT-COF | 39.3 | Ru 0.56wt%  Ir 0.52wt% | 1 M KOH | **This work** |
| Ru@2H-MoS_2_ | 51 | Ru 10.73 wt% | 1 M KOH | 1 7 |
| Ru SAs-Ni_2_P | 57 | Ru 2.20 wt% | 1 M KOH | 2 8 |
| CoRu-MoS_2_ | 52 | Ru 20 at% | 1 M KOH | 3 9 |
| Ru@MoS_2_@CNTs | 50 | Ru 5 at% | 1 M KOH | 4 10 |
| SA-Ru-MoS_2_ | 76 | Ru 5 wt% | 1 M KOH | 5 11 |
| Ni_3_N/Ru/NCAC composites | 42 | Ru 0.3 wt% | 1 M KOH | 6 12 |
| Ru SAs/N-Mo_2_C NSs | 43 | Ru 2.39 wt% | 1 M KOH | 7 13 |
| Pt@PCM | 139 | Pt 0.53 wt% | 1 M KOH | 8 14 |
| Ru/C_3_N_4_/C | 79 | Ru 20 wt% | 1 M KOH | 9 15 |
| Ru/GDY | 44 | Ru 0.96 wt% | 1 M KOH | 10 16 |
| Ru/S–Ni_2_P | 49 | / | 1 M KOH | 11 17 |
| Ru-ZIF-900 | 51.6 | Ru 0.18 wt% | 1 M KOH | 12 18 |
| Ru–NiSe_2_/NF | 59 | Ru 2.76 wt% | 1 M KOH | 13 19 |
| Ru_SA_-N-S-Ti_3_C_2_T_x_ | 99 | Ru 1.2 wt% | 1 M KOH | 14 20 |
| S-RuP@NPSC | 92 | Ru 2.76 wt% | 1 M KOH | 15 21 |

**Table S7** OER performance comparison of the IrRu DAS/AT-COF with other state-of-the-art noble-metal-based electrocatalysts reported previously in 1 M KOH.

| Catalysts | η_10_  (mV) | Noble metal loading | Electrolyte | Ref. |
| --- | --- | --- | --- | --- |
| IrRu DAS/AT-COF | 251.2 | Ru 0.56wt%  Ir 0.52wt% | 1 M KOH | **This work** |
| Ir-rEGO | 293 | Ir 2.1 wt% | 1 M KOH | 16 22 |
| Ru-FeRu@C/NC | 345 | Ru 23.13 wt% | 1 M KOH | 17 23 |
| RuTe_2_ PNRs | 285 | Ru 33.3 at% | 1 M KOH | 18 24 |
| Ir-NSs | 266 | / | 1 M KOH | 19 25 |
| Ir_1_@Co/NC | 260 | Ir 2.28 wt % | 1 M KOH | 20 26 |
| Ir-modified BP | 290 | Ir 36.41 at% | 1 M KOH | 21 27 |
| Ru_3_Ni_3_ NAs | 304 | Ru 20 wt % | 1 M KOH | 22 28 |
| RuNi-NCNFs | 290 | Ru 28.2 wt% | 1 M KOH | 23 29 |
| IrO_2_/V_2_O_5_ | 283 | Ir 19.7 wt% | 1 M KOH | 24 30 |
| Ir-NR/C | 296 | Ir 4.95 wt% | 1 M KOH | 25 31 |
| Ir-C≡ | 300 | Ir 20 wt% | 1 M KOH | 26 32 |
| Ir@NG-750 | 303 | Ir 3.21 wt% | 1 M KOH | 27 33 |
| Co@Ir/NC | 280 | Ir 7.46 wt% | 1 M KOH | 28 34 |
| IrO_2_-RuO_2_/C | 270 | Ru 41.28 wt%  Ir 10.06 wt% | 1 M KOH | 29 34 |
| IrCo@NC-850 | 302 | Ir 1.47 wt% | 1 M KOH | 30 36 |

**Table S8** Performance comparison of the IrRu DAS/AT-COF‖IrRu DAS/AT-COF with other previously reported state-of-the-art catalysts for overall water splitting in 1M KOH.

| Catalysts | η_10_/mV | Substrate | Electrolyte | Ref. |
| --- | --- | --- | --- | --- |
| IrRu DAS/AT-COF\|\|IrRu DAS/AT-COF | 1.61 | Nickel foam | 1 M KOH | **This work** |
| CoP@FeCoP/NC \|\| CoP@FeCoP/NC | 1.68 | Carbon cloth | 1 M KOH | 37 |
| Ni-CoP/HPFs \|\| Ni-CoP/HPFs | 1.68 | Glassy carbon | 1 M KOH | 38 |
| Co_3_O_4_@Ni_3_Se_4_ \|\| Co_3_O_4_@Ni_3_Se_4_ | 1.74 | Nickel foam | 1 M KOH | 39 |
| Co@Co_3_O_4_-NC \|\| Co@Co_3_O_4_-NC | 2.00 | Glassy carbon | 1 M KOH | 40 |
| CoS_2_-MoS_2_ \|\| CoS_2_-MoS_2_ | 1.68 | Nickel foam | 1 M KOH | 41 |
| CoP/CNFs \|\| CoP/CNFs | 1.65 | Glassy carbon | 1 M KOH | 42 |
| NESSP//NESS \|\| NESSP//NESS | 1.77 | Stainless steel | 1 M KOH | 43 |
| Mo-Ni_3_S_2_ \|\| Mo-Ni_3_S_2_ | 1.67 | Nickel foam | 1 M KOH | 44 |
| (BO_3_)_X_-Ni_3_S_4_ \|\| (BO_3_)_X_-Ni_3_S_4_ | 1.69 | Glassy carbon | 1 M KOH | 45 |
| Cu-Ni-S \|\| Cu-Ni-S | 1.88 | Carbon cloth | 1 M KOH | 46 |
| NiCo_2_S_4_/ReS_2_ \|\| NiCo_2_S_4_/ReS_2_ | 1.63 | Carbon cloth | 1 M KOH | 47 |
| NiSe \|\| NiSe | 1.63 | Nickel foam | 1 M KOH | 48 |
| CoFeZrO \|\| CoFeZrO | 1.63 | Nickel foam | 1 M KOH | 49 |
| CoP/NCNHP \|\| CoP/NCNHP | 1.64 | Carbon paper | 1 M KOH | 50 |
| CoSe \|\| CoSe | 1.65 | Ti mesh | 1 M KOH | 51 |
| NiFeO_x_/CNF \|\| NiFeO_x_/CNF | 1.65 | Carbon fiber | 1 M KOH | 52 |
| S-NiFe_2_O_4_/NF \|\| S-NiFe_2_O_4_/NF | 1.65 | Nickel foam | 1 M KOH | 53 |
| NiS_2_-MoS_2_ \|\| NiS_2_-MoS_2_ | 1.67 | Nickel foam | 1 M KOH | 54 |
| CoMoO/CoMoP \|\| CoMoO/CoMoP | 1.68 | Nickel foam | 1 M KOH | 55 |
| CoS-RGO \|\| CoS-RGO | 1.77 | RGO | 1 M KOH | 56 |
| CoSe-Co(OH)_2_ \|\| CoSe-Co(OH)_2_ | 1.65 | / | 1 M KOH | 57 |
| MoS_2_-NiS_2_/NGF \|\| MoS_2_-NiS_2_/NGF | 1.64 | Graphene foam | 1 M KOH | 58 |
| Fe-MoS_2_/CoMo_2_S_4_ \|\| Fe-MoS_2_/CoMo_2_S_4_ | 1.62 | Glassy carbon | 1 M KOH | 59 |

**Table S9** Structural parameters extracted from the quantitative EXAFS curve-fitting under operando conditions.

| Catalysts | Metal | Bond type | N | R  (Å) | σ^2^×10^3^ (Å^2^) | ΔE_0_  (eV) | R-factor |
| --- | --- | --- | --- | --- | --- | --- | --- |
| Ex-situ | Ir L-edge | Ir-N | 2.0 | 2.12 | 7.7±7.2 | -6.8±0.4 | 0.02 |
|  |  | Ir-Cl | 3.0 | 2.38 | 9.2±0.8 |  |  |
|  | Ru K-edge | Ru-N | 2.0 | 2.09 | 9.7±1.1 | -4.5±7.6 | 0.02 |
|  |  | Ru-Cl | 3.0 | 2.35 | 9.4±8.9 |  |  |
| At open circuit | Ir L-edge | Ir-N | 1.1 | 2.04 | 7.9±6.9 | 1.9±8.0 | 0.02 |
|  |  | Ir-Cl | 2.9 | 2.35 | 3.9±1.6 |  |  |
|  | Ru K-edge | Ru-N | 1.1 | 1.94 | 3.9±1.1 | 7.5±1.3 | 0.02 |
|  |  | Ru-O | 1.1 |  |  |  |  |
|  |  | Ru-Cl | 2.9 | 2.22 | 8.2±2.3 |  |  |
| At -0.1 V | Ir L-edge | Ir-N | 1.0 | 1.96 | 5.6±4.3 | 5.1±7.6 | 0.02 |
|  |  | Ir-Cl | 2.9 | 2.28 | 9.0±6.3 |  |  |
|  | Ru K-edge | Ru-N | 1.0 | 1.96 | 3.1±0.9 | 5.9±3.1 | 0.02 |
|  |  | Ru-O | 1.0 |  |  |  |  |
|  |  | Ru-Cl | 2.9 | 2.24 | 9.1±3.4 |  |  |

N, coordination number; R, distance between absorber and backscatter atoms; ΔE_0_, inner potential correction to account for the difference in the inner potential between the catalyst and the reference compound. σ^2^, Debye-Waller factor.

**Supplemental References**

1. L. Ran, Z. W. Li, B. Ran, J. Q. Cao, Y. Zhao, T. Shao, Y. R. Song, M. K. H. Leung, L. C. Sun, J. A. Hou, *J. Am. Chem. Soc.* **2022,** *144*, 17097-17109.
2. S. J. Clark, M. D. Segall, C. J. Pickard, P. J. Hasnip, M. J. Probert, K. Refson, M. C. Payne, *Z. Kristallogr.* **2005,** *220*, 567-570.
3. J. P. Perdew, K. Burke, M. Ernzerhof, *Phys. Rev. Lett.* **1996,** *77*, 3865-3868.
4. P. J. Hasnip, C. J. Pickard, *Comput. Phys. Commun.* **2006,***174*, 24-29.
5. J. P. Perdew, J. A. Chevary, S. H. Vosko, K. A. Jackson, M. R. Pederson, D. J. Singh, C. Fiolhais, *Phys. Rev. B* **1992,** *46*, 6671-6687.
6. J. D. Head, M. C. Zerner, *Chem. Phys. Lett.* **1985,** *122*, 264-270.
7. J. Wang, W. H. Fang, Y. Hu, Y. H. Zhang, J. Q. Dang, Y. Wu, B. Z. Chen, H. Zhao, Z. X. Li, *Appl. Catal., B* **2021,** 298, 120490.
8. K. L. Wu, K. A. Sun, S. J. Liu, W. C. Cheong, Z. Chen, C. Zhang, Y. Pan, Y. S. Cheng, Z. W. Zhuang, X. W. Wei, Y. Wang, L. R. Zheng, Q. H. Zhang, D. S. Wang, Q. Peng, C. Chen, Y. D. Li, *Nano Energy* **2021,** 80*,* 105467.
9. H. B. Zhang, P. F. An, W. Zhou, B. Y. Guan, P. Zhang, J. C. Dong, X. W. Lou, *Sci. Adv.* **2018,** 4*, eaao6657*.
10. I. S. Kwon, T. T. Debela, I. H. Kwak, Y. C. Park, J. Seo, J. Y. Shim, S. J. Yoo, J. G. Kim, J. Park, H. S. Kang, *Small* **2020,** 16*,* 2000081.
11. X. Zhang, F. Zhou, S. Zhang, Y. Y. Liang, R. H. Wang, *Adv. Sci.* **2019,** 6, 1900090.
12. J. M. Zhang, X. P. Xu, L. Yang, D. J. Cheng, D. P. Cao, *Small Methods* **2019,** 3, 1900653.
13. Z. L. Chen, R. B. Wu, Y. Liu, Y. Ha, Y. H. Guo, D. L. Sun, M. Liu, F. Fang, *Adv. Mater.* **2018,** 30, 1802011.
14. X. Zhang, X. L. Yu, L. J. Zhang, F. Zhou, Y. Y. Liang, R. H. Wang, *Adv. Funct. Mater.* **2018,** 28, 1706523.
15. Y. Zheng, Y. Jiao, Y. H. Zhu, L. H. Li, Y. Han, Y. Chen, M. Jaroniec, S. Z. Qiao, *J. Am. Chem. Soc.* **2016,** 138, 16174-16181.
16. Y. Gu, A. P. Wu, Y. Q. Jiao, H. R. Zheng, X. Q. Wang, Y. Xie, L. Wang, C. G. Tian, H. G. Fu, *Angew. Chem., Int. Ed.* **2021,** 60, 6673-6681.
17. Y. Huang, X. N. Song, J. Deng, C. Y. Zha, W. J. Huang, Y. L. Wu, Y. G. Li, *Appl. Catal., B* **2019,** 245, 656-661.
18. S. F. Wu, M. Y. Chen, W. W. Wang, J. B. Zhou, X. R. Tang, D. L. Zhou, C. Liu, *Carbon* **2021,** 171, 385-394.
19. X. Y. Yan, J. Biemolt, K. Zhao, Y. Zhao, X. J. Cao, Y. Yang, X. Y. Wu, G. Rothenberg, N. Yan, *Nat. Commun.* **2021,** 12, 4143.
20. V. Ramalingam, P. Varadhan, H. C. Fu, H. Kim, D. L. Zhang, S. M. Chen, L. Song, D. Ma, Y. Wang, H. N. Alshareef, J. H. He, *Adv. Mater.* **2019,** 31, 1903841.
21. F. S. Farahani, M. S. Rahmanifar, A. Noori, M. F. El-Kady, N. Hassani, M. Neek-Amal, R. B. Kaner, M. F. Mousavi, *J. Am. Chem. Soc.* **2022,** 144, 3411-3428.
22. G. Z. Li, T. Sun, H. J. Niu, Y. Yan, T. Liu, S. S. Jiang, Q. L. Yang, W. Zhou, L. Guo, *Adv. Funct. Mater.* **2023,** 33, 2212514.
23. W. Q. Li, H. Zhang, K. Zhang, W. X. Hu, Z. Z. Cheng, H. P. Chen, X. Feng, T. Peng, Z. K. Kou, *Appl. Catal., B* **2022,** 306*,* 121095.
24. G. Meng, H. Tian, L. X. Peng, Z. H. Ma, Y. F. Chen, C. Chen, Z. W. Chang, X. Z. Cui, J. L. Shi, *Nano Energy* **2021,** 80, 105531.
25. H. Li, M. T. Zhang, L. C. Yi, K. Chen, P. Shao, Z. H. Wen, *Appl. Catal., B* **2021,** 280, 119412.
26. Y. Liu, X. Li, Q. H. Zhang, W. D. Li, Y. Xie, H. Y. Liu, L. Shang, Z. Y. Liu, Z. M. Chen, L. Gu, Z. Y. Tang, T. R. Zhang, S. Y. Lu, *Angew. Chem., Int. Ed.* **2020,** 59, 1718-1726.
27. H. G. Liu, Z. Hu, Q. L. Liu, P. Sun, Y. F. Wang, S. L. Chou, Z. Z. Hu, Z. Q. Zhang, *J. Mater. Chem. A* **2020,** 8, 24710-24717.
28. J. Y. Yu, G. X. Li, H. Liu, L. L. Zhao, A. Z. Wang, Z. Liu, H. D. Li, H. Liu, Y. Y. Hu, W. J. Zhou, *Adv. Funct. Mater.* **2019,** 29, 1901154.
29. J. Wang, Z. Z. Wei, S. J. Mao, H. R. Li, Y. Wang, *Energy Environ. Sci.* **2018,** 11, 800-806.
30. W. J. Kang, Y. Feng, Z. Li, W. Q. Yang, C. Q. Cheng, Z. Z. Shi, P. F. Yin, G. R. Shen, J. Yang, C. K. Dong, H. Liu, F. X. Ye, X. W. Du, *Adv. Funct. Mater.* **2022,** *32*, 2112367.
31. F. Luo, L. Guo, Y. H. Xie, J. X. Xu, K. G. Qu, Z. H. Yang, *Appl. Catal., B* **2020,** 279, 119394.
32. L. H. Fu, X. Hu, Y. B. Li, G. Z. Cheng, W. Luo, *Nanoscale* **2019,** 11, 8898-8905.
33. Z. L. Chen, H. L. Qing, R. R. Wang, R. B. Wu, *Energy Environ. Sci.* **2021,** 14, 3160-3173.
34. S. J. Shen, Z. P. Wang, Z. P. Lin, K. Song, Q. H. Zhang, F. Q. Meng, L. Gu, W. W. Zhong, *Adv. Mater.* **2022,** 34, 2110631.
35. Y. T. Li, F. Q. Chu, Y. F. Bu, Y. Kong, Y. X. Tao, X. Zhou, H. R. Yu, J. J. Yu, L. Tang, Y. Qin, *Chem. Commun.* **2019,** 55, 7828-7831.
36. J. Yang, Y. Shen, Y. M. Sun, J. H. Xian, Y. J. Long, G. Q. Li, *Angew. Chem., Int. Ed.* **2023,** 62, e202302220.
37. J. H. Shi, F. Qiu, W. B. Yuan, M. M. Guo, Z. H. Lu, *Chem. Eng. J.* **2021,** 403, 126312.
38. Y. Pan, K. A. Sun, Y. Lin, X. Cao, Y. S. Cheng, S. J. Liu, L. Y. Zeng, W. C. Cheong, D. Zhao, K. L. Wu, Z. Liu, Y. Q. Liu, D. S. Wang, Q. Peng, C. Chen, Y. D. Li, *Nano Energy* **2019,** 56, 411-419.
39. R. H. Que, G. Ji, D. S. Liu, M. L. Li, S. Liu, *Chemnanomat* **2019,** 5, 814-819.
40. C. D. Bai, S. S. Wei, D. R. Deng, X. D. Lin, M. S. Zheng, Q. F. Dong, *J. Mater. Chem. A* **2017,** 5, 9533-9536.
41. Y. Wang, Y. L. Zhu, S. Afshar, M. W. Woo, J. Tang, T. Williams, B. Kong, D. Y. Zhao, H. T. Wang, C. Selomulya, *Nanoscale* **2019,** 11, 3500-3505.
42. X. Q. Xie, J. P. Liu, C. N. Gu, J. J. Li, Y. Zhao, C. S. Liu, *J. Energ. Chem.* **2022,** 64, 503-510.
43. M. S. Balogun, W. T. Qiu, Y. C. Huang, H. Yang, R. M. Xu, W. X. Zhao, G. R. Li, H. B. Ji, Y. X. Tong, *Adv. Mater.* **2017,** 29, 1702095.
44. C. R. Wu, B. T. Liu, J. Wang, Y. Y. Su, H. Q. Yan, C. T. Ng, C. Li, J. M. Wei, *Appl. Surf. Sci.* **2018,** 441, 1024-1033.
45. Z. Zhang, T. R. Zhang, J. Y. Lee, *Chemelectrochem* **2019,** 6, 1443-1449.
46. D. Chinnadurai, S. J. Lee, Y. Yu, S. Y. Nam, M. Y. Choi, *Fuel* **2022,** 320, 123915.
47. C. G. Pei, M. C. Kim, Y. K. Li, C. K. Xia, J. Kim, W. So, X. Yu, H. S. Park, J. K. Kim, *Adv. Funct. Mater.* **2023,** 33, 2210072.
48. Y. Y. Zhong, B. Chang, Y. L. Shao, C. W. Xu, Y. Z. Wu, X. P. Hao, *Chemsuschem* **2019,** 12, 2008-2014.
49. L. L. Huang, D. W. Chen, G. Luo, Y. R. Lu, C. Chen, Y. C. Zou, C. L. Dong, Y. F. Li, S. Y. Wang, *Adv. Mater.* **2019,** 31, 1901439.
50. Y. Pan, K. A. Sun, S. J. Liu, X. Cao, K. L. Wu, W. C. Cheong, Z. Chen, Y. Wang, Y. Li, Y. Q. Liu, D. S. Wang, Q. Peng, C. Chen, Y. D. Li, *J. Am. Chem. Soc.* **2018,** 140, 2610-2618.
51. T. Liu, Q. Liu, A. M. Asiri, Y. Luo, X. Sun, *Chem. Commun.* **2015,** 51, 16683-16686.
52. H. Wang, H.-W. Lee, Y. Deng, Z. Lu, P.-C. Hsu, Y. Liu, D. Lin, Y. Cui, *Nat. Commun.* **2015,** 6, 7261.
53. J. L. Liu, D. D. Zhu, T. Ling, A. Vasileff, S. Z. Qiao, *Nano Energy* **2017,** 40, 264-273.
54. Z. C. Li, Y. Xu, X. X. Ren, W. P. Wang, *J. Mater. Sci.* **2020,** 55, 13892-13904.
55. Y. Zhang, Q. Shao, S. Long, X. Q. Huang, *Nano Energy* **2018,** 45, 448-455.
56. Y. N. Chen, S. M. Xu, S. Z. Zhu, Y. J. Li, J. Q. Dai, F. J. Chen, H. Xie, B. Y. Liu, Y. G. Yao, L. G. Salamanca-Riba, M. R. Zachariah, T. Li, L. B. Hu, *Nano Res.* **2019,** 12, 2259-2267.
57. C. Gong, W. X. Li, Y. N. Lei, X. He, H. Chen, X. Du, W. Fang, D. H. Wang, L. Zhao, *Composites, Part B* **2022,** **236**, 109823.
58. P. Y. Kuang, M. He, H. Y. Zou, J. G. Yu, K. Fan, *Appl. Catal., B* **2019,** 254, 15-25.
59. Y. N. Guo, J. Tang, J. Henzie, B. Jiang, W. Xia, T. Chen, Y. Bando, Y. M. Kang, S. A. Hossain, Y. Sugahara, Y. Yamauchi, *ACS Nano* **2020,** 14, 4141-4152.
